# MycoNews 2021: President’s message, IMA statutes, news, reports, awards, personalia, and book news

**DOI:** 10.1186/s43008-021-00085-9

**Published:** 2021-12-31

**Authors:** David L. Hawksworth

**Affiliations:** 1 Comparative Fungal Biology, Royal Botanic Gardens, Kew, TW9 3DS Surrey UK; 2 Department of Life Sciences, The Natural History Museum, Cromwell Road, London, SW7 5BD UK; 3 Jilin Agricultural University, Changchun, 130118 Jilin Province China

**Keywords:** Birthday tributes, Book reviews, Funga, International Mycological Congress, Meeting reports, Obituaries

## Abstract

This third annual edition of *MycoNews* starts with a message from IMA President Wieland Meyer regarding the adoption of new statutes for the IMA, the postponement of IMC12 to 2024, and announcing Marc Stadler as President-elect. The new statutes are included in full. News is provided on the launch of a World Fungus Day, acceptance of the term Funga as an equivalent to Fauna and Flora by the IUCN Species Survival Commission, new arrangements and dates for IMC12 now to be held in Maastricht in July 2024, and revised arrangements for the publication of proposals to change any rules governing the nomenclature of fungi. Reports are provided for IAL9, the symposium of the International Association for Lichenology in Brazil mainly conducted virtually, MycoRise Up! in Poland, and the centenary of the German Mycological Society (DGFM). Birthday greetings from IMA go to David Farr, Marie-Agnés Letrouit-Galinou, Maria Olech, Angela Restrepo, Carol Shearer, James Trappe, and Shun-ichi Udagawa. Tributes are also paid to the passing of the distinguished mycologists Heinz Butin, Karl Esser, Grégoire Hennebert, Jack Rogers, Kálman Vánky, and Bodo Wanke. The contribution concludes with news of seven new mycological books published in 2020–2021, and another forthcoming in 2022.

## PRESIDENT'S MESSAGE 2021

(Fig. 1)

**Fig. 1:**
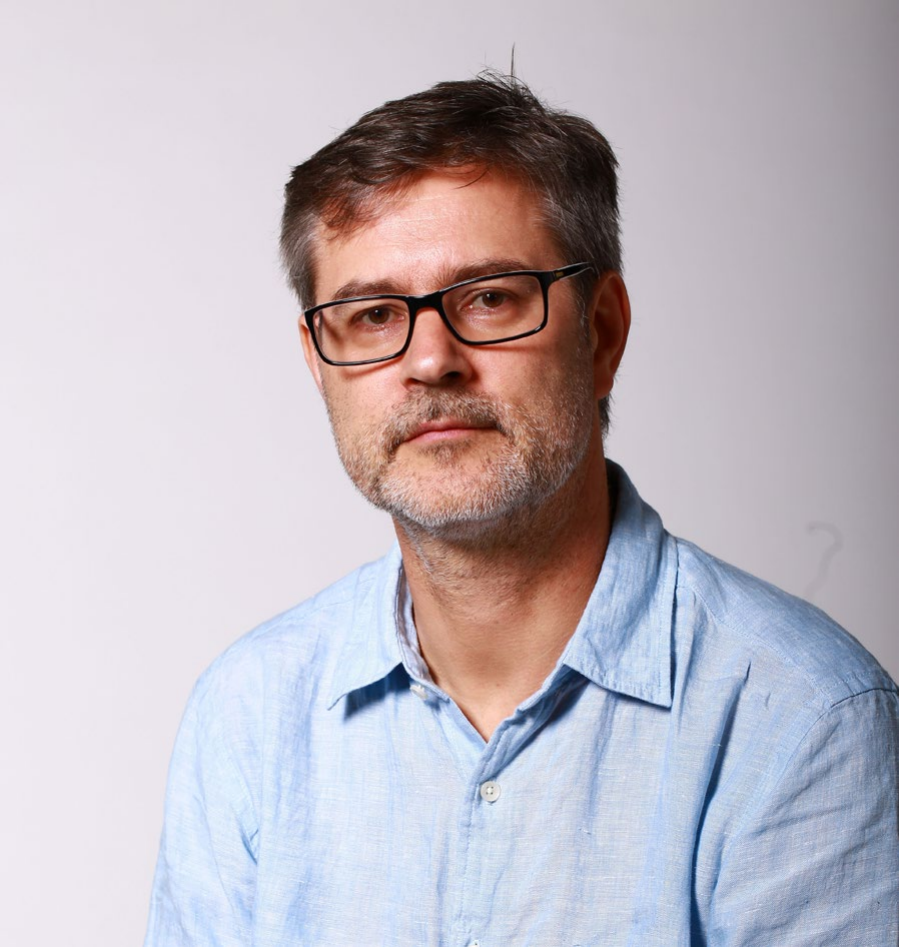
Wieland Meyer

Fellow Mycologists, another challenging year has passed, and the world is slowly starting to work out how to live with COVID-19 by striving to vaccinate its population to enable a return to some kind of normality. We saw scientific meetings postponed, moved to a hybrid format of in person and online, or even totally online. This has broad new challenges but also in many cases allowed us to talk more often, through the various online portals, with our collaborators overseas or interstate.

The IMA Executive, and especially its officers have used this new way of communication with the appointed Swiss law firm, Ludwig & Partners in Basel, to advance the course of its incorporation in Switzerland. Through many meetings we have successfully completed the new statutes of IMA, which have undergone an extensive way of consultation and finally been accepted by the IMA Executive members. They are reproduced following this Message (pp. 4–14).

The major changes in the IMA Statutes are:

1. Membership is open to all individuals and legal entities with an interest in mycology. IMA now has ordinary members, encompassing individual and student members; collective members, including regional member mycological organizations (RMMOs), sustaining member mycological organizations (SMMOs) and member mycological organizations (MMOs); and honorary members.
2. Individual membership fees can now be renewed directly without attending an International Mycological Congress (IMC) in addition to the normal road of payment as part of the registration fee of an IMC.
3. The major governing bodies of IMA are now: The general Assembly, The Council, The Executive Committee and The Auditors which will be appointed according to the Statutes.
4. The newly instated IMA Council will replace the original officers and will now consist of: The President, President-elect, Past-President, General Secretary, Treasurer, Chair of the previous IMC, and Chair of the next IMC.

The position of President-elect was newly created to enable continuity within the IMC Council. It allows the incoming President to gain knowledge on how IMA is run on a daily basis. The IMA organised an open call for nominations for the post of President-elect in March 2021, where two nominations for candidates for the office were received, Sharon A. Cantrell-Rodríquez (Department of Natural Sciences and Technology, Universidad Ana G Méndez, Gurabo, Puerto Rico) and Marc Stadler (Department Microbial Drugs, Helmholtz Centre for Infection Research, Braunschweig, Germany). Both are longstanding members of the IMA Executive Committee with a huge body of scientific research in mycology. Following the nominations, the independent company Woods & Watlen invited the 31 members of the IMC Executive, including its officers, to vote from 4–9. October 2021. I thank both candidates for standing for election, and congratulate Marc Stadler on being elected as President-elect.

The next step of the incorporation of IMA in Switzerland is a founding meeting of the IMA under Swiss law. This will take place virtually by the end of December 2021 with the participation of all current IMA Officers, Executive Members, and representatives of the RMMOs, SMMOs and MMOs. The newly incorporated IMA, its governing bodies, and the new Statutes will be formally ratified at the next General Assembly of IMA during the next IMC in 2024. I would like to thank all IMC Executive Members for their support during this sometimes complicated process, especially Orlando Petrini (our link in Switzerland to our lawyers at Ludwig & Partners), Jennifer Luangsa-ard (IMA General Secretary) and Keith Seifert (IMC Past-President) for their tireless work on shaping the new IMA Statutes during the past two years, and further Karen Hansen for her continuing support as acting Treasurer who with Andrey Yurkov handled the IMA finances.

I would like to thank the following IMA Executive members who left the Executive due to changeovers in the leadership of their respective national or regional mycological societies for their work for IMA: Leona Campbell (former President of the Australasian Mycological Society), XingZhong Liu (President of the Asian RMMO), Eduardo Alvarez Duarte (former President of the Latin American Mycological Association), Kentaro Hosaka (Representative of the Mycological Society of Japan), and Joey Spatafora (Representative of the Mycological Society of America and the North American RMMO).

I wish to welcome the following new members of the IMA Executive Committee, representing changes of leadership in national or regional mycological societies: Tracey Steinrucken (President of the Australasian Mycological Society), Mary Catherine Aime (Representative of the Mycological Society of America and the North American RMMO), Saisamorn Lumyong (President of the Asian Mycological Association), Yuuri Hirooka (Representative of the Mycological Society of Japan), and Luis C. Mejia (President of the Latin American Mycological Association).

During 2021, IMA and the organizing committee of the next IMC took the hard decision to postpone IMC12 from July 2022 to July 2024 because of the COVID-19 pandemic and the resulting travel restrictions, as we all thought that the IMC should remain the major platform where mycologists from all over the world can meet in person to discuss scientific results, matters of mycology, and especially make new personal connections with each other. We also decided to move IMC12 from Amsterdam to Maastricht, offering a new venue in the centre of Europe, easily accessible from all parts of the world. The IMA Council will visit Maastricht in April 2022 in association with its next Council/Executive meeting to be held in connection with the forthcoming Westerdijk “Rise of Fungi” symposium. We are all looking forward to an exciting scientific program at IMC12, which is currently being developed under the Chair of the scientific organizing committee, Teun Boekhout. More information on IMC12 is provided on pp. 15–18.

In 2021, two fungi in particular put mycology on the world stage, making the front page of news outlets globally for their devastating impact on human health. The “first”, the yeast *Candida auris,* which was first isolated a few years ago from an ear-infections in a Japanese patient, is causing major hospital outbreak globally, showing five major genetic subpopulations. It seemingly appearing out of nowhere until its first environmental niche was found earlier this year in seaweed off the cost of the Andaman Islands in the Indian Ocean. The second disease is mucormycosis causing large numbers of invasive fungal infections in COVID-19 patients with many deaths, especially in India and South America. As such, the unique initiative by Lynne Boddy (Cardiff School of Biosciences, UK), to establish a World Fungus Day to promote, raise awareness and draw attention to fungi globally is very timely. The First World Fungal Day took place on 2 October 2021, with a very successful virtual symposium including speakers from all parts of the globe. Further information is provided below. The IMA is the logical major international organization to support the World Fungus Day initiative, and the IMA Council will work together with Lynne Boddy to make this a major global annual mycology event.

As President of IMA, I am looking forward to working with all members of the IMA Council and the IMA Executive for its membership, and especially on preparing an exciting IMC12 programme to promote mycology globally.

I wish you all a safe upcoming festive season and a great start to the New Year.

**Wieland Meyer**

*President, IMA*

(wieland.meyer@sydney.edu.au)

## REVISED STATUTES OF THE IMA

**Fig. 2:**
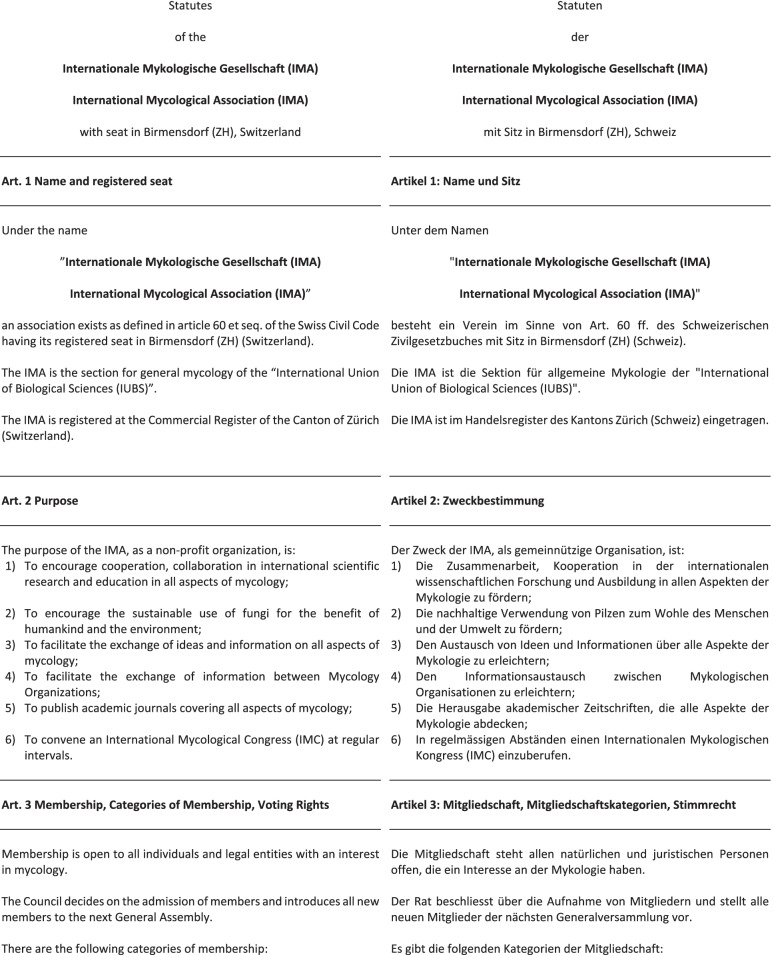

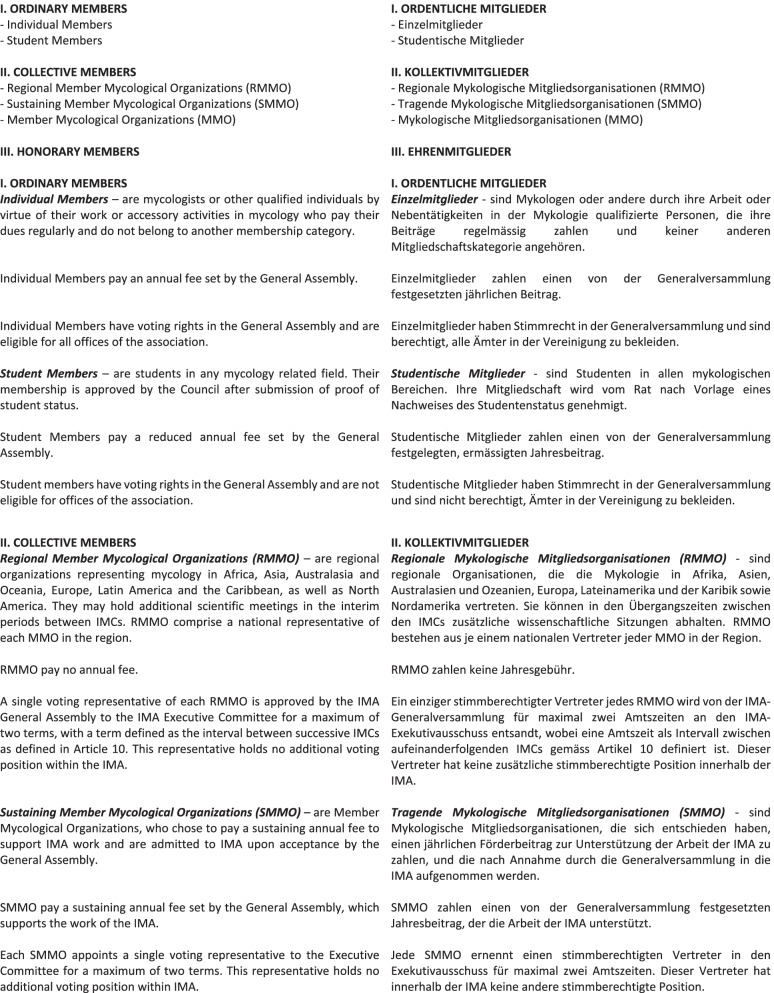

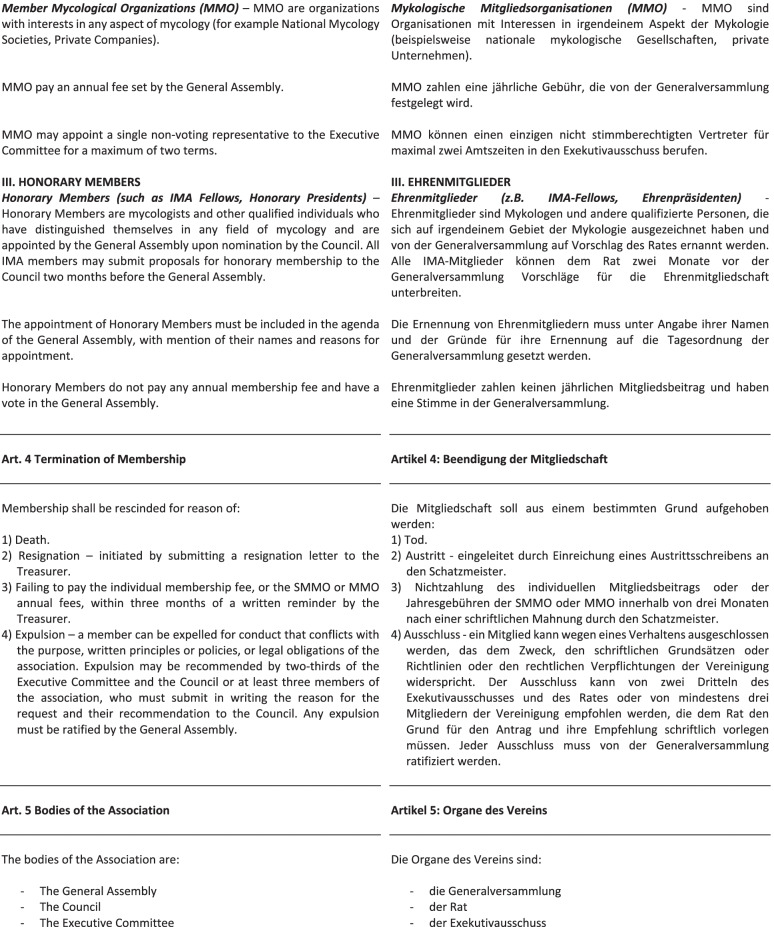

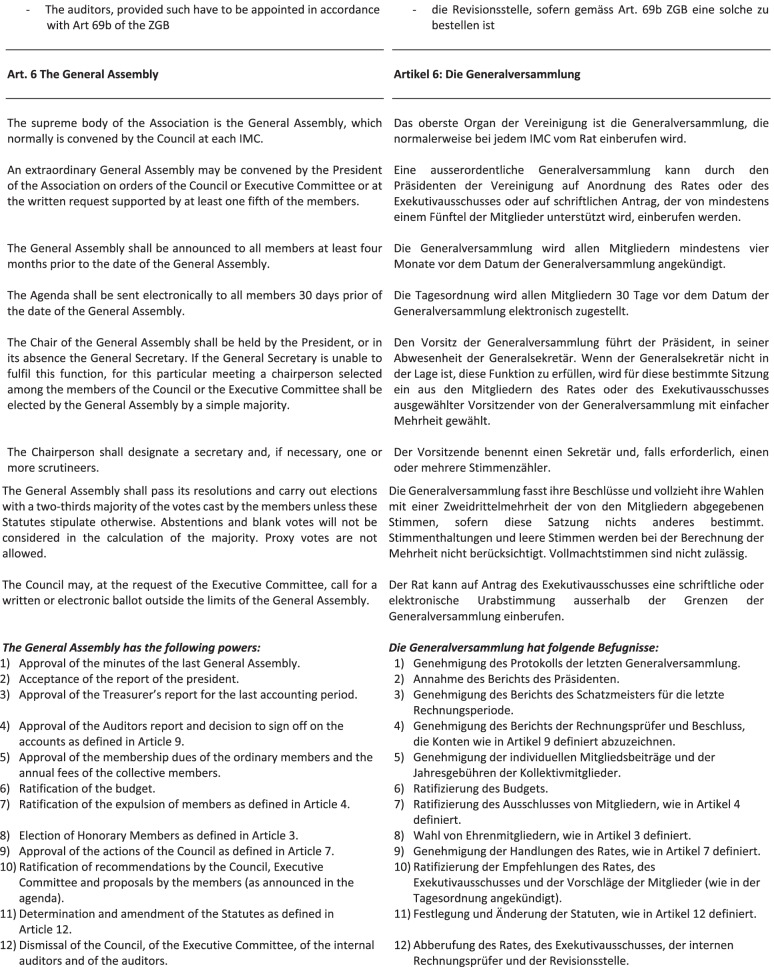

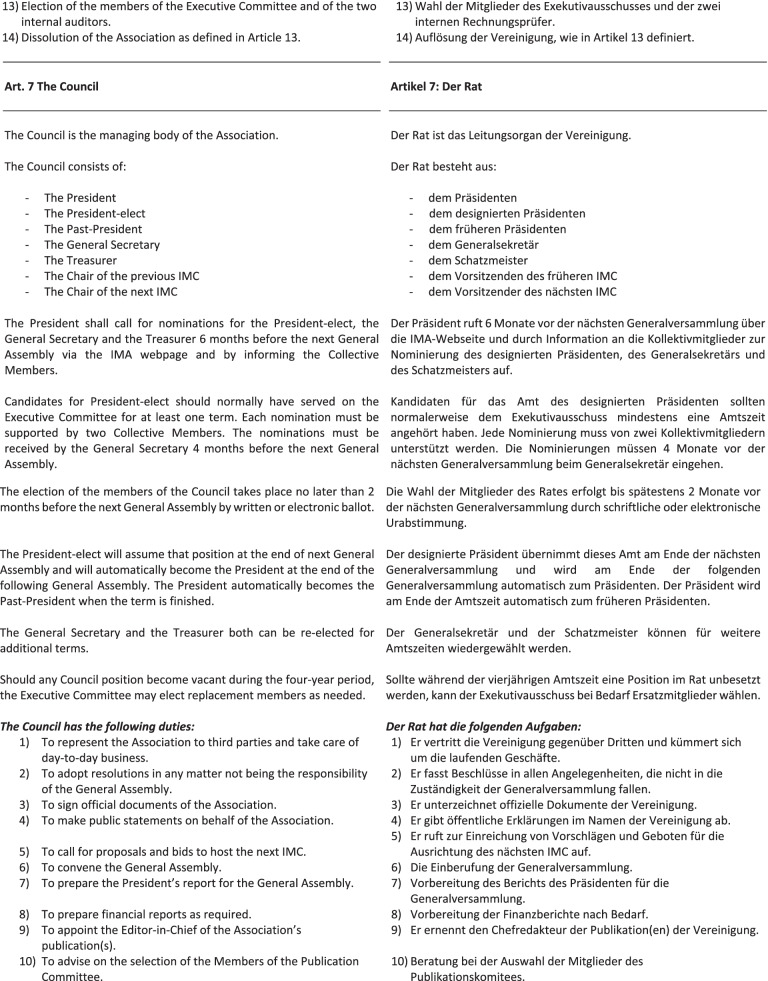

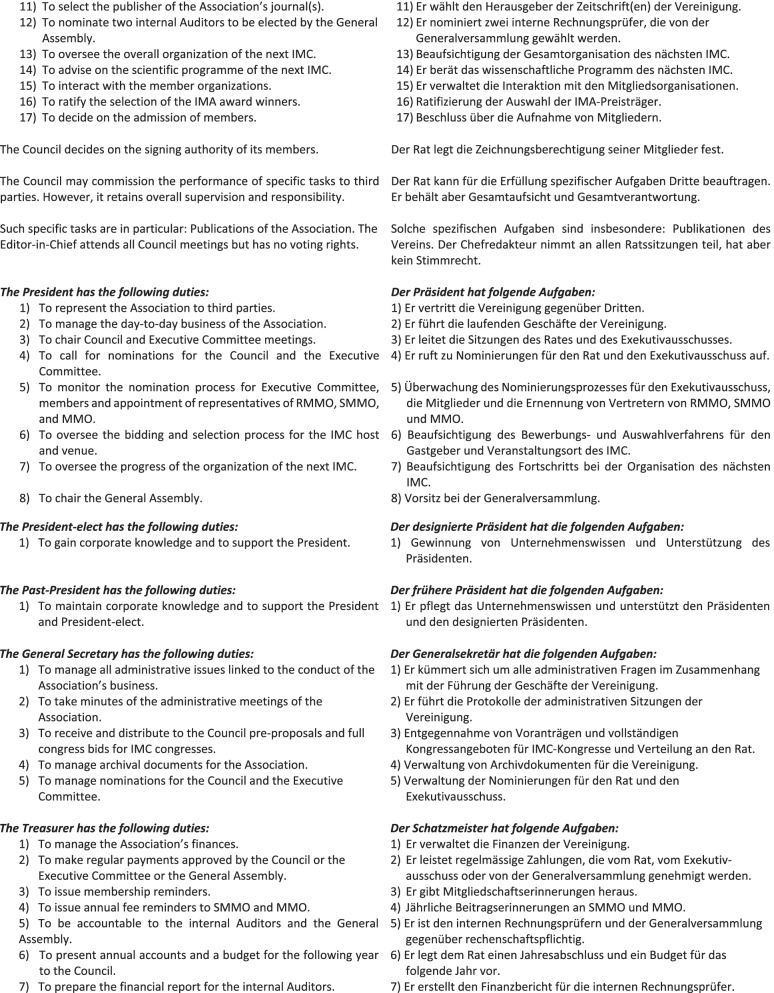

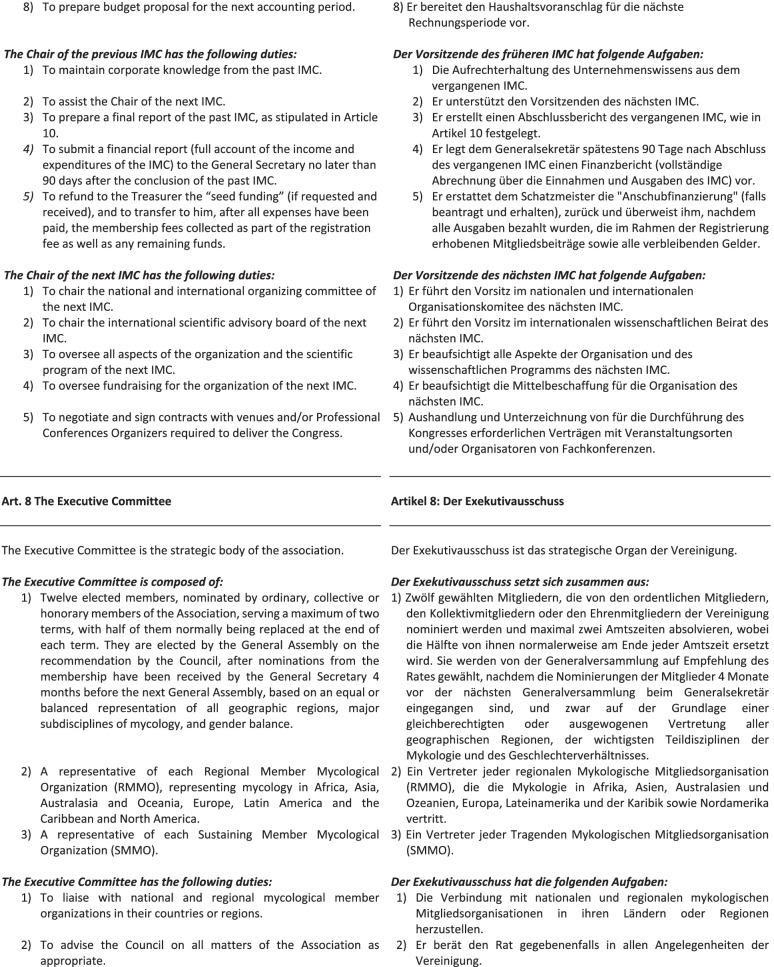

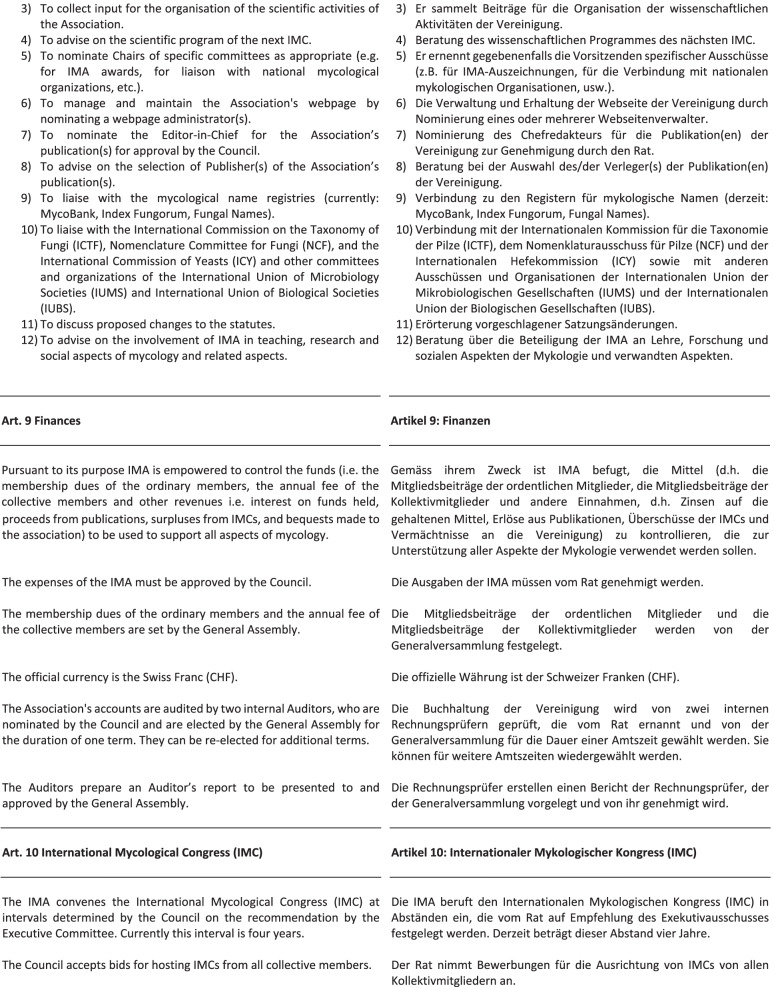

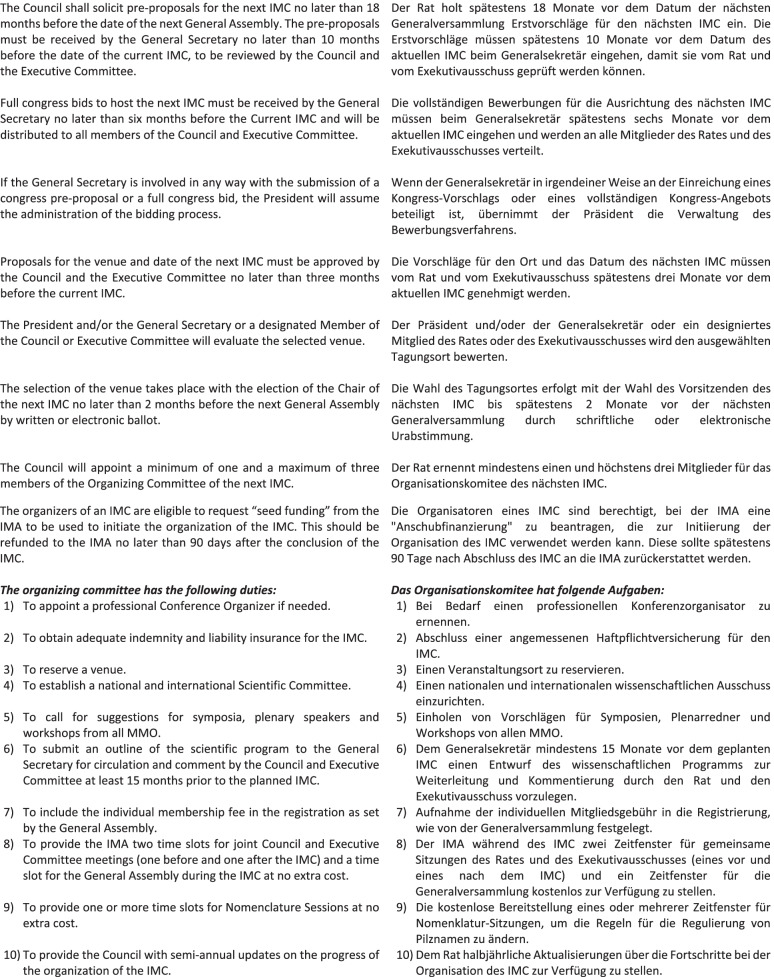

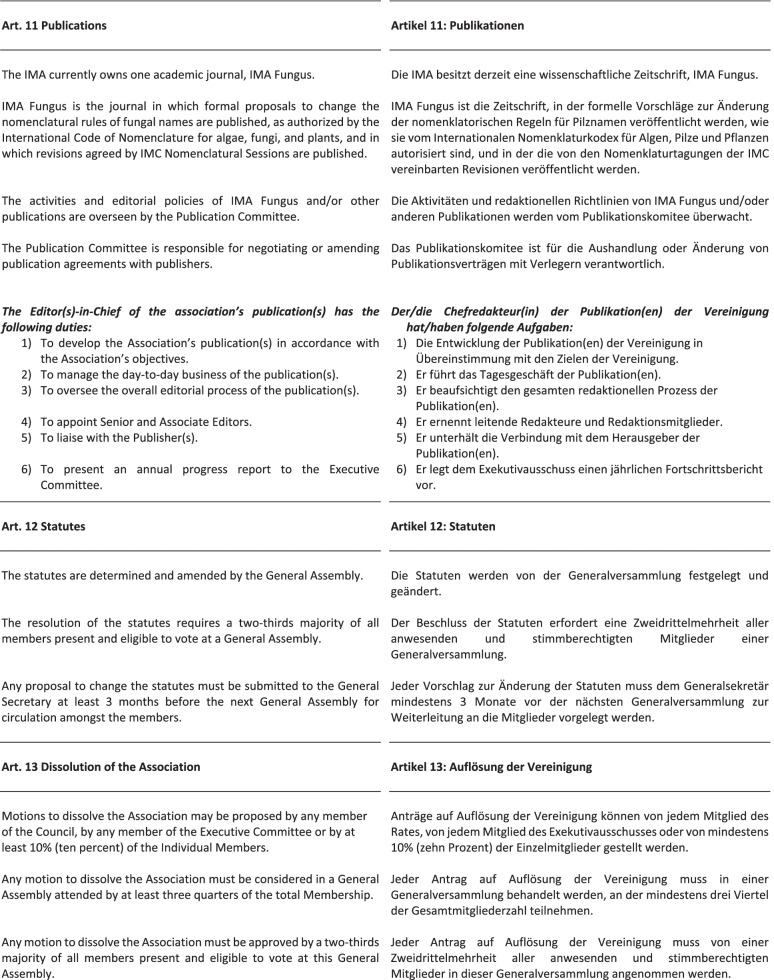

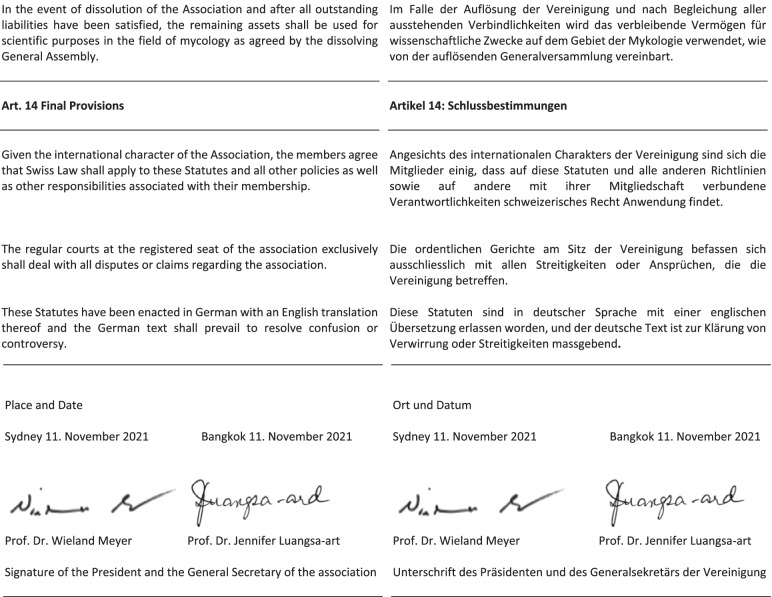
New statutes of the International Mycological Association

These are reproduced in full here (Fig. 2), pp. 4–14.

## WORLD FUNGUS DAY LAUNCHED

(Fig. 3)

**Fig. 3:**
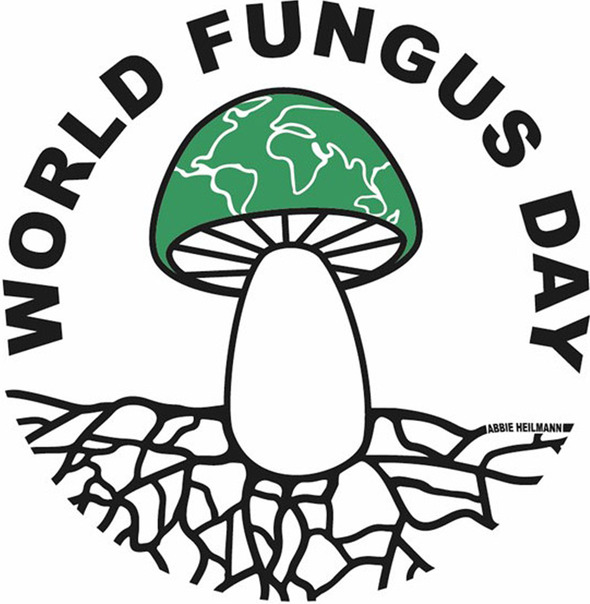
The World Fungus Day twitter (@FungusDay) icon designed by 17 year-old Abbie Heilmann

Although we all know that fungi are crucial to our very existence, most lay people, and even many biologists, do not realise this. They do not know that our planet’s ecosystems would not function without fungi—there would be no fertile soil, no plant life, no herbivores, no carnivores and no humans. This matters from many perspectives. For example, when people understand and care about fungi, there will be a much greater chance that they will protect fungi, and even invest in trying to find out more about them.

In the UK, as in many other places, we have for a long time put on events to introduce the general public to fungi. If you attended IMC9 in 2010 you may have visited the “Amazing Fungi” exhibition at the Royal Botanic Garden, Edinburgh. In 2011 it moved to the National Botanic Garden of Wales, where I was privileged to open it. After the opening, we had a brainstorming session to come up with ideas on how to broaden awareness of fungi. Someone asked whether there was a world fungus day. The answer at the time was sadly no, so we decided to aim to have one. We started on a small scale and organised Wales Fungus Day in 2012. The next year we extended it to UK Fungus Day, and in 2013, there were 50 events across the country. UK Fungus Day has gone on from strength to strength, though in 2020 it had to be entirely on-line because of Covid (Porritt 2020), and this year was a hybrid of on-line and face to face events.

Usually we have indoor events, outdoor events, and combinations of both, including talks, displays, films, fungal inspired fashion, arts and crafts, growing edible fungi, making fungus models, fungal microscopy, spore printing, quizzes etc. etc., and, of course, forays and organised fungus walks. Some of these are illustrated in the special editions of the BMS newsletters reporting on the annual event (https://www.britmycolsoc.org.uk/society/publications) and on the UK Fungus Day website (http://www.ukfungusday.co.uk/).

A World Fungus Day, would have far greater impact than national fungus days or more disparate fungal events. It would be unrealistic to have a huge World Fungus Day instantly, and cynics say that we could never get a day that all Mycological Societies would agree upon, but if one does not try to start something then it will never happen. So, this year, to start the ball rolling, we organised an online 1-day international symposium with talks from or about all continents, and hosted by the UK Arboricultural Society. Talks covered Kauri dieback in New Zealand, mangroves in India, fungi in Africa, truffles in Europe, wildfires in North America, fungi attacking historic structures in the Arctic and Antarctic, marine fungi, global food security, ‘intelligence’ of fungi, battles between fungi, and “Funga: the F word we all need to say” (see below) (https://www.trees.org.uk/Training-Events/World-Fungi-Day). It was highly successful, and in future years we must aim for events that are even more diverse and more widely attendable.

I was rather late in contacting mycological societies about the idea of a World Fungus Day this year. Nonetheless, some groups were able to mobilise rapidly. For example, the Mycological Society of America were highly supportive putting forward the idea in ‘Inoculum’ and on their website, as well as rebadging events that occurred on or around this day. The Mycological Society of India ran two week-long events (for example https://youtu.be/IbviwU_qEa0), which include talks, competitions and much more.

I am hoping that national and regional mycological societies will take this idea on board and start to think about events that could be put on in their localities for a wider launch of the 2nd World Fungus Day in October 2022. It is unlikely to be possible nor desirable to try to co-ordinate activities from a central site, but it would be good to aim for a World Fungus Day website that has links to the national/regional websites.

So I am appealing to all mycologists to start thinking about events and logistics for World Fungus Day 2022 and contacting me.

**Lynne Boddy**

(BoddyL@cardiff.ac.uk)

## IUCN SSC ACCEPTANCE OF FAUNA–FLORA–FUNGA

(Fig. 4)

**Fig. 4:**
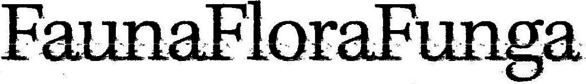
Fauna-Flora-Fungo logo

The issue of whether the term “Funga” should be adopted as an equivalent to Fauna and Flora has been a subject of debate since the word was apparently first coined by Gravesen (2000) reporting on a conference on indoor air held in Edinburgh in 1999. The desirability of this term has been discussed in several subsequent publications (e.g. Hawksworth 2001a; b), and was soon taken up in several regional fungal works (e.g. Knudsen and Vesterholt 2008). It was most strongly advocated by Kuhar et al. (2018) who discuss the origins and use. Arguments for and against were elaborated upon only last year by Zmitrovich (2020).

**Fig. 5:**
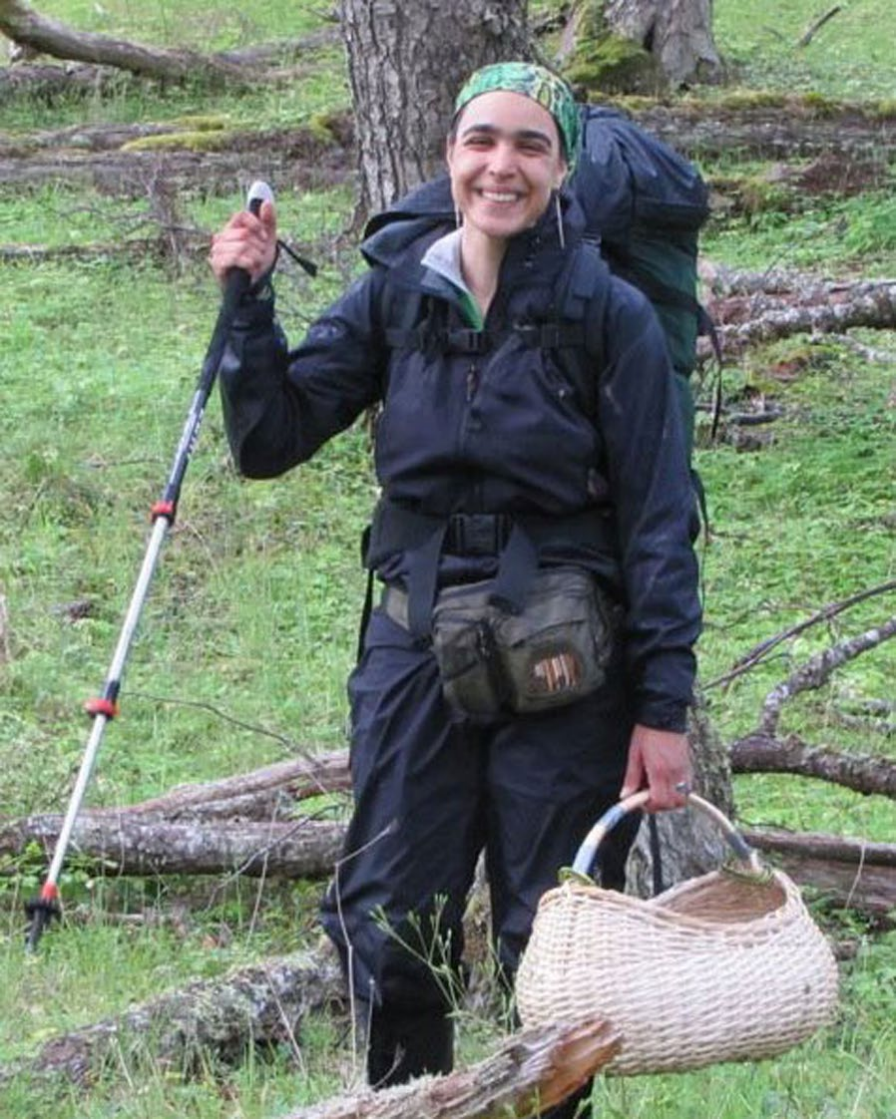
Giuliana Furci, collecting mushrooms in Tierra del Fuego in 2014

Giuliana Furci (Fig. 5), Merlin Sheldrake, and César Rodgríguez-Garavito, supported by numerous other mycologists around the world, presented a call for change to the delayed IUCN (International Union for the Conservation of Nature) World Conservation Congress 2020 in Montpellier, France, in September 2021: “FaunaFloraFunga: let’s recognise fungi within conservation frameworks”.

Thanks to the leadership of the Chilean Fungi Foundation, both Re:wild and the IUCN Species Survival Commission (SSC), in advance of the start of the Congress, announced on 3 August 2021 their commitment to use “mycologically inclusive” language in their internal and public-facing communications (“fauna, flora and funga” and “animals, fungi and plants”) and to incorporate fungi in conservation strategies with rare and endangered plants and animals. The IUCN Species Survival Commission called for the due recognition of fungi as major components of biodiversity in legislation and policy. It fully endorsed the Fauna Flora Funga Initiative and asks that the phrases animals and plants, and fauna and flora, be replaced with animals, fungi, and plants.

It will now be for mycologists worldwide to actively promote this usage of Funga wherever appropriate to reinforce the distinctness and importance of fungi as a component of biodiversity and of fundamental ecological and ecosystem processes.

For further information contact: Gregory Mueller (Chair, IUCN SSC Fungal Conservation Committee) and the associated press release: https://www.iucn.org/commissions/species-survival-commission/about/ssc-committees/fungal-conservation-committee.

## IMC12: FUNGAL BIOLOGY AND APPLICATIONS

(Fig. 6)

**Fig. 6:**
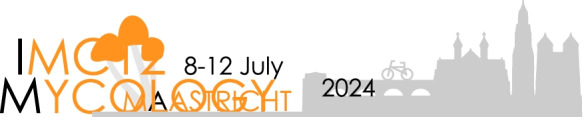
IMC12 logo

As you are probably aware, the International Mycological Congress (IMC12), which was originally scheduled for July 2022 in Amsterdam, was postponed to 2024 due to the COVID-19 pandemic. However, we are happy to announce that we have a new date and venue, namely 8–12 July 2024, in the MECC Conference Centre in Maastricht (in the South of The Netherlands). Maastricht is located in the heart of the Euregion, the area where The Netherlands, Germany and Belgium meet, home to four million people living in these three countries and where four different languages are spoken.

Because we wanted to retain the July timeslot, we had to relocate to another congress venue and city in The Netherlands. However, the MECC Conference Centre has the space to accommodate the exhibits and keynote lectures, as well as breakout rooms to facilitate all secondary meetings that usually get planned during an IMC congress. Furthermore, because of this new location, the mycological societies from nearby European countries will also be able to make a more significant input to the final programme. The MECC in Maastricht is also easily accessible by train via several major airports, or directly by plane to the Maastricht-Aachen Airport (10 km from MECC Maastricht and only 15 min away by car, taxi or bus).

The Dutch Mycological Society, in collaboration with the Westerdijk Fungal Biodiversity Institute, is therefore pleased to invite you to attend the 12th International Mycological Congress in Maastricht. We are excited for the return of this incredible mycological experience to Europe, and to The Netherlands specifically.

In January 2023, there will be a call for **Symposia**, and **Workshops**. We will keep you posted!

The Scientific Themes have been established, and the chairs appointed. Each theme also has its own Scientific Committee.

**General chair**: Pedro Crous

**Scientific Chair**: Teun Boekhout

**Theme1: Cell biology, biochemistry and physiology**

Chair 1, Ida van der Klei, The Netherlands

Chair 2, Gregory Jedd, Singapore

Chair 3, Marcio Rodriques, Brazil

**Theme 2: Environment, ecology and interactions**

Chair 1, Lynne Body, UK,

Chair 2, Duur Aanen, NL,

Chair 3, Yu Fukasawa, Japan,

**Theme 3: Evolution, biodiversity and systematics**

Chair 1, Jos Houbraken, NL

Chair 2, Ester Gaya, UK

Chair 3, Lei Cai, China

*Keynote lectures*: *Toni Gabaldón* (Spain), and *Jolanta Maria Miadlikowska* (USA)

**Theme 4: Fungal pathogenesis and disease control**

Chair 1, Dee Carter, Australia

Chair 2, Martijn Rep, The Netherlands

Chair 3, Juan McEwen, Colombia

**Theme 5: Genomics, genetics, and molecular biology**

Chair 1, Miia Mäkelä, Finland

Chair 2, Ronald de Vries, NL

Chair 3: Brenda Wingfield, South Africa

**Theme 6: Nomenclature**

Chair 1, Tom May, Australia

Chair 2, Konstanze Bensch, Germany

Chair 3, Bevan Weir, New Zealand

*Keynote lectures*: *M. Catherine Aime* (USA), and *Robert Lücking* (Germany)

**Theme 7: Applied mycology**

Chair 1, Lene Jesperson, Denmark

Chair 2, Richard Bélanger, Canada

Chair 3, Nancy Keller, USA

### Further keynotes announced!

Details of the keynote speakers who had already agreed to participate were included in MycoNews 2020 (*IMA Fungus* **11**(28): 4–5, 2020) and are noted above. Each keynote session will have two speakers and the following additional ones can now be announced:

### Cell biology, biochemistry and physiology

***Kaustuv Sanyal***, India

(Fig. 7)

**Fig. 7:**
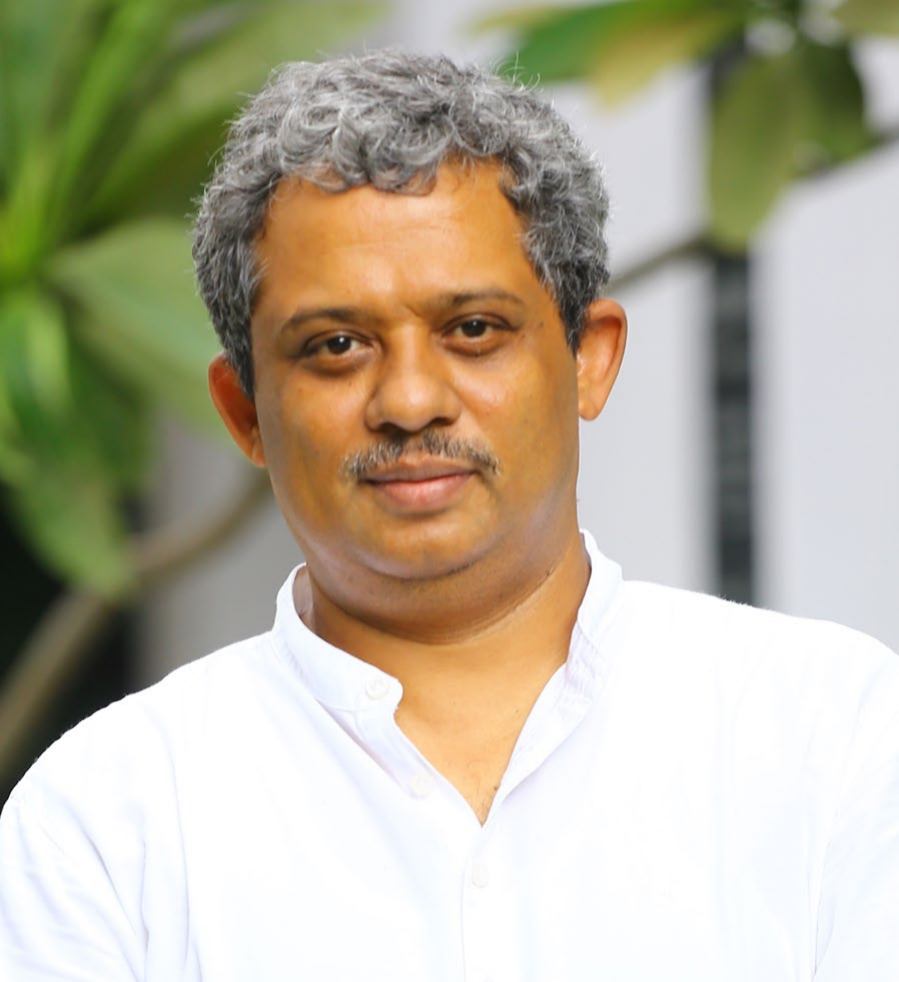
Kaustuv Sanyal

Kaustuv is an elected fellow of the American Academy of Microbiology (ASM, USA), JC Bose National Fellow and Professor of Molecular Mycology at the JN Centre for Advanced Scientific Research, Bangalore. The major focus of his research is to understand the mechanism of chromosome segregation using various fungi, both pathogenic and non-pathogenic, as model systems. He is also interested in the mechanism of genome indexing in unicellular organisms by histone variants. He has been awarded the prestigious Tata Innovation Fellowship and National Bioscience Award by the Department of Biotechnology (India), is an elected fellow the Indian National Science Academy (New Delhi), Indian Academy of Sciences (Bangalore, India), the National Academy of Sciences (Allahabad, India) and a nominated member of the Faculty of 1000 (F1000Prime), UK. He is a visiting professor at the Osaka University, Japan. Currently, he leads a vibrant group of graduate students and postdoctoral researchers, and actively collaborates with many research groups across the world.

***Meritxell Riquelme***, Mexico

(Fig. 8)

**Fig. 8:**
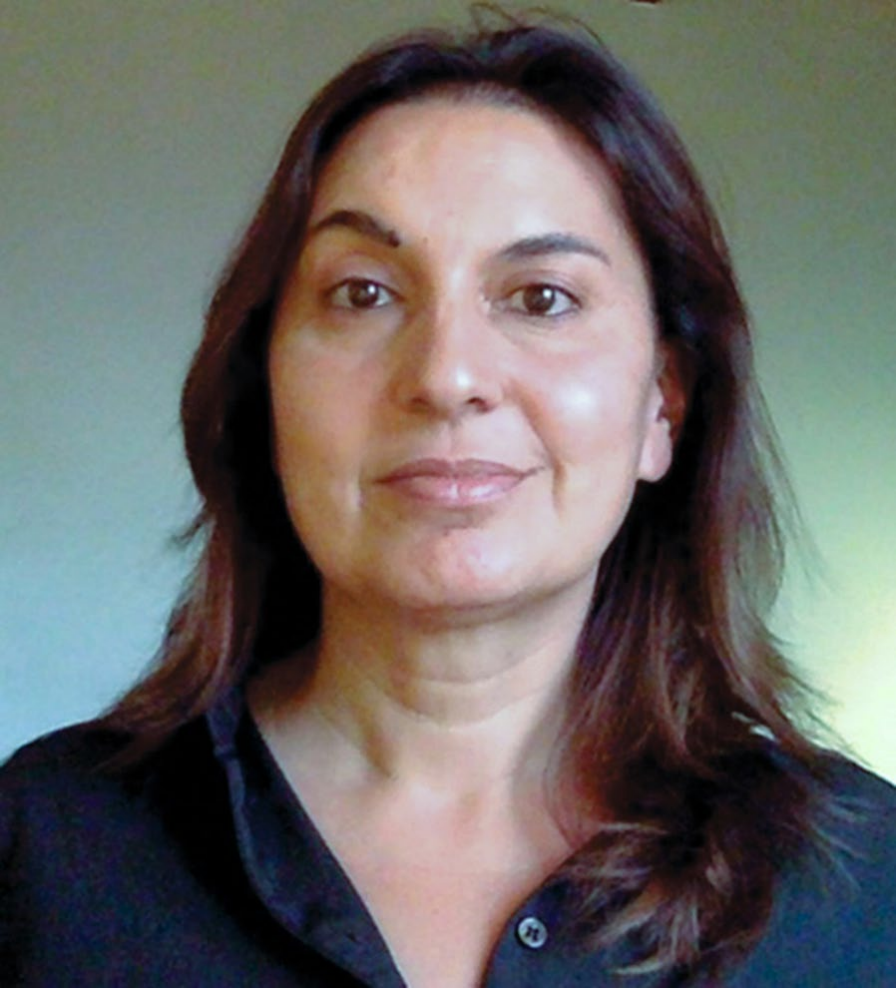
Meritxell Riquelme

Meritxell is a Research Professor and Chair of the Department of Microbiology at the Center for Scientific Research and Higher Education of Ensenada (CICESE), Baja California, Mexico, where she has been on the faculty since 2004. She obtained a BA degree in Biology at the University of Barcelona, Spain. She received a MSc degree in Plant Pathology and a PhD in Microbiology from the University of California, Riverside. During her PhD thesis in S. Bartnicki-Garcia’s laboratory, she investigated the role of the Spitzenkörper in hyphal morphology and growth. She was a postdoctoral fellow at the University of Oxford, UK, in the laboratory of Lorna Casselton, where she worked on the receptor and pheromone mating type genes of *Coprinopsis cinerea*. Her current research focuses on understanding basic aspects of hyphal morphogenesis in fungi. By combining advanced microscopy and molecular biology she investigates the biochemical role and secretory routes of vesicles involved in the polar apical growth of hyphae of *Neurospora crassa*. Additional research interests have led her to study the ecological distribution of the human pathogen *Coccidioides* spp., a fungus that causes coccidiodomycosis or Valley Fever in the semi-arid regions of Baja California. More recently she has studied the fungal diversity of deep-sea sediments of the Gulf of Mexico. She is a member of the Mexican Academy of Sciences. In 2018 she received the B. O. Dodge award for her contributions to the *Neurospora* research community and in 2019 she was elected Fellow of the Mycological Society of America. She is editor of the journals *Fungal Genetics and Biology, The Cell Surface, Communications Biology*, and *Frontiers in Cell and Developmental Biology*. Previously she was editor of Fungal Biology. She has served in the Mycological Society of America (MSA) as member of the Karling Lecture Committee (2005–2008, chair 2007–2008), of the Genetics and Cell Biology Committee (2008–2010, chair 2009–2010), and as councilor for Cell Biology/Physiology (2014–2016). She served as member of the Neurospora Policy Committee (2008–2012), the Fungal Genetics Policy Committee (2013–2019), the International Fungal Biology Conference Steering Committee (since 2014), and the Executive Committee of the International Mycological Association (since 2014).

### Environment, ecology and interactions

***Håvard Kauserud***, Norway

(Fig. 9)

**Fig. 9:**
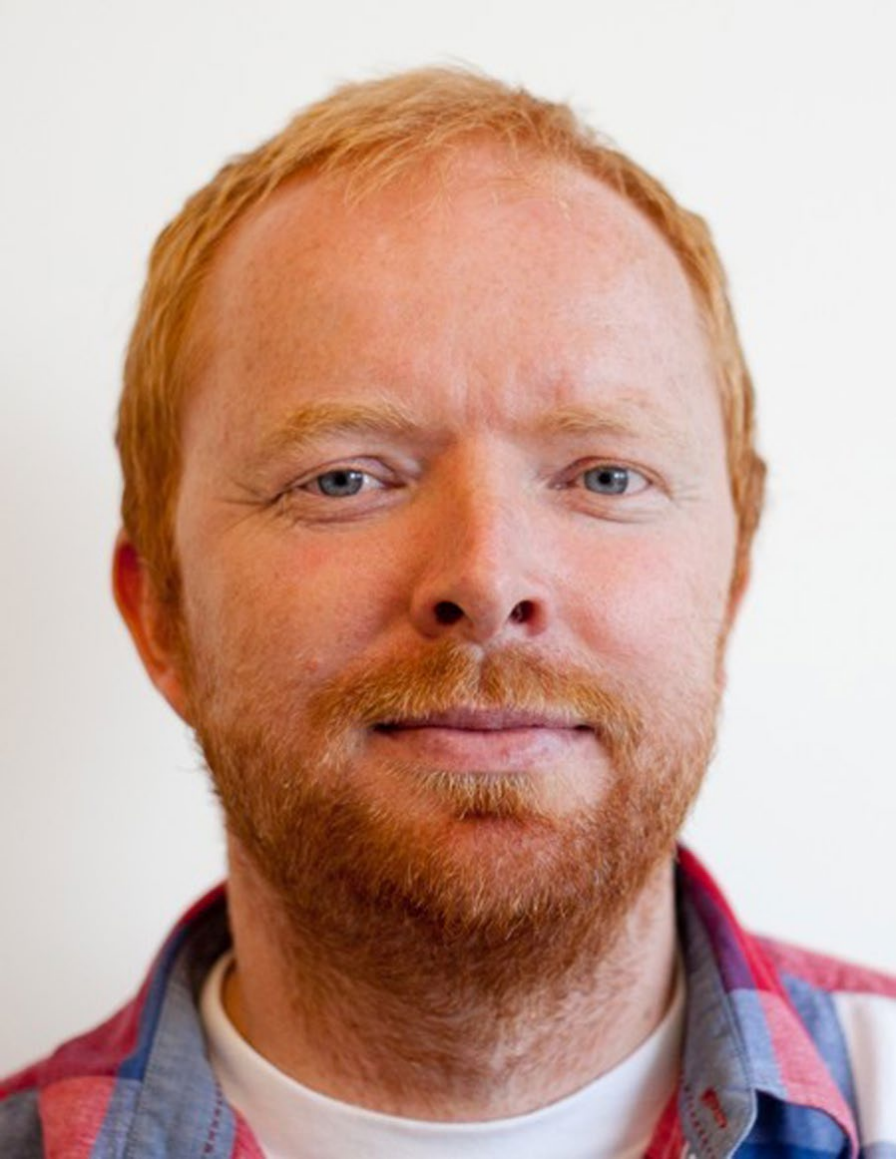
Håvard Kauserud

Håvard is Professor in Biology at the University of Oslo, and has a broad focus on fungal ecology and biology. One main research avenue has been to assess how fungal communities are affected and structured by environmental variability and change, and how fungal traits influence on community assembly. In most studies, fungal communities have been surveyed by DNA metabarcoding analyses, but he has also used historic sporocarp observations to assess how fungal communities and fungal life-history events changes through time.

***Maiko Kagami***, Japan

(Fig. 10)

**Fig. 10:**
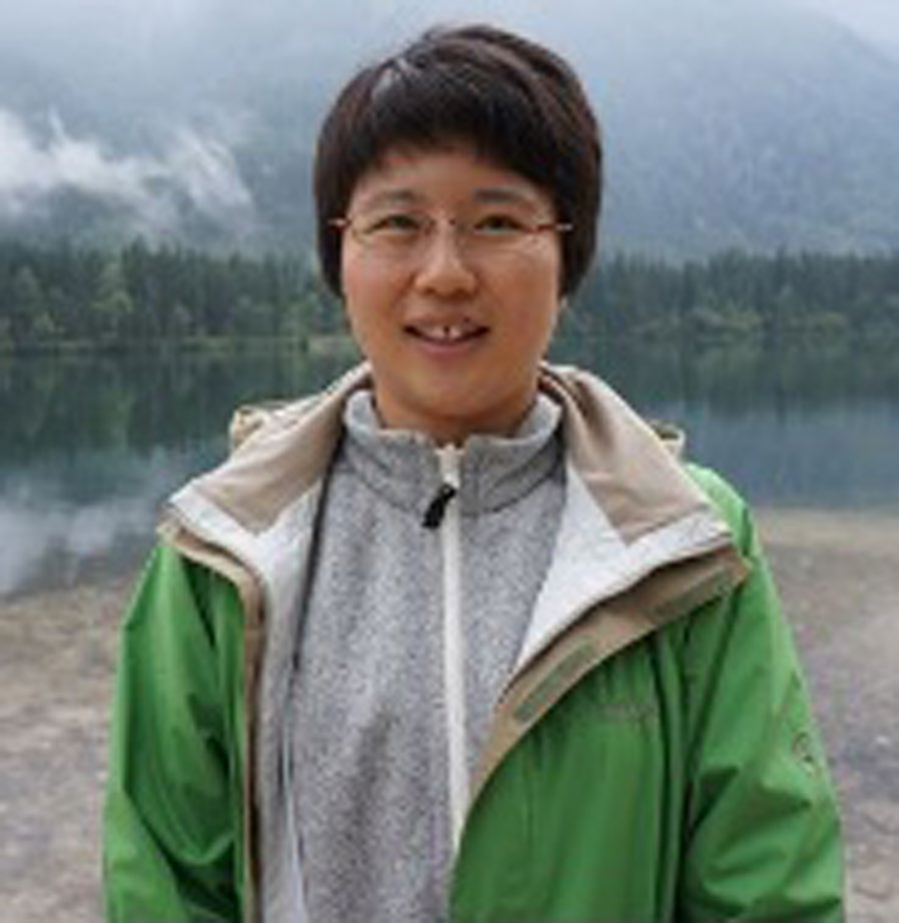
Maiko Kagami

Maiko is a Professor in Graduate school of Environment and Information Sciences, Yokohama National University, Kanagawa, Japan, since April 2018. Her research interests lie in aquatic ecology, examining the dynamics and roles of plankton and microbes in biogeochemical cycling. Maiko completed her PhD at Kyoto University, Japan in 2002. During her PhD, she examined plankton dynamics in Lake Biwa, and found the importance of parasitic fungi infecting phytoplankton. From 2002 to 2006, she worked as a JSPS research fellow (postdoc) in The Netherlands Institute of Ecology (NIOO-KNAW), Center for Limnology. She discovered material flow via parasitic fungi in aquatic food webs, which was named as Mycoloop after Mycology and her name (Maiko). From September 2006, she became a faculty member, lecturer (2006–2012) and associate professor (2012–2018) in the Department of Environmental Science, Faculty of Science, Toho University, Chiba, Japan. She continued her research in aquatic ecology. Maiko has actively collaborated with oversea researchers, particularly Germany and The Netherlands. She has been PIs of several Grants-in-Aid (KAKENHI), including those promoting international collaborations. From 2016 to 2018, she has been a PI of the Fund for the Promotion of Joint International Research (Fostering Joint International Research) and stayed for 1.5 years as a guest researcher at Leibniz-Institute of Freshwater Ecology and Inland Fisheries (IGB-Berlin) in Germany and at The Netherlands Institute of Ecology (NIOO-KNAW) in The Netherlands.

For the latest information on IMC12, please go to www.imc12.org.

**Pedro Crous**

(p.crous@wi.knaw.nl)

## FUNGAL NOMENCLATURE PROPOSALS

**New deadline for proposals to amend Chapter F of the International Code of Nomenclature for algae, fungi, and plants to be considered at IMC 12, 2024**

*Chapter F* of the *International Code of Nomenclature for algae, fungi and plants* (*Code*) contains provisions of the *Code* that are specific to fungi (May et al. 2019). Proposals to amend *Chapter F* are considered at the Fungal Nomenclature Session of an International Mycological Congress (IMC). Proposals to amend the *Code* as a whole, excluding *Chapter F*, are considered at the Nomenclature Section of an International Botanical Congress (IBC). Instructions for submitting proposals to amend *Chapter F* were published in *IMA Fungus* (May 2020).

The 20th IBC has been rescheduled from 2023 to 21–24 July 2024 in Madrid, and the Nomenclature Section of that Congress will meet on 15–19 July. The 12th IMC has now been rescheduled from 2022 to 18–12 July 2024 in Maastricht (*see above*) so any mycologists wishing to do so will be able to attend both congresses. Consequently, the deadline for submission of proposals to amend *Chapter F* is now **31 December 2023**, i.e. two years later than originally scheduled (May 2020). Late submissions received by the end of January 2024 may be accepted if no reviewing or major editing is necessary. The “Synopsis of proposals” will appear in March 2024 and the pre-Congress guiding vote will be held in May 2024 (with results disseminated in June 2024). See May (2020) for details of how to prepare and submit proposals.

For those wishing to amend or introduce provisions of the *Code* that are not specific to fungi, the deadline for proposals to be considered at the Nomenclature Section of the 20th IBC has been rescheduled to 31 March 2023. See Turland and Wiersema (2019) for details of how to prepare and submit such proposals.

**Tom W. May**

*Secretary, Fungal Nomenclature Bureau*

(tom.may@rbg.vic.gov.au)

## IAL9: INTERNATIONAL ASSOCIATION FOR LICHENOLOGY

(Fig. 11)

**Fig. 11:**
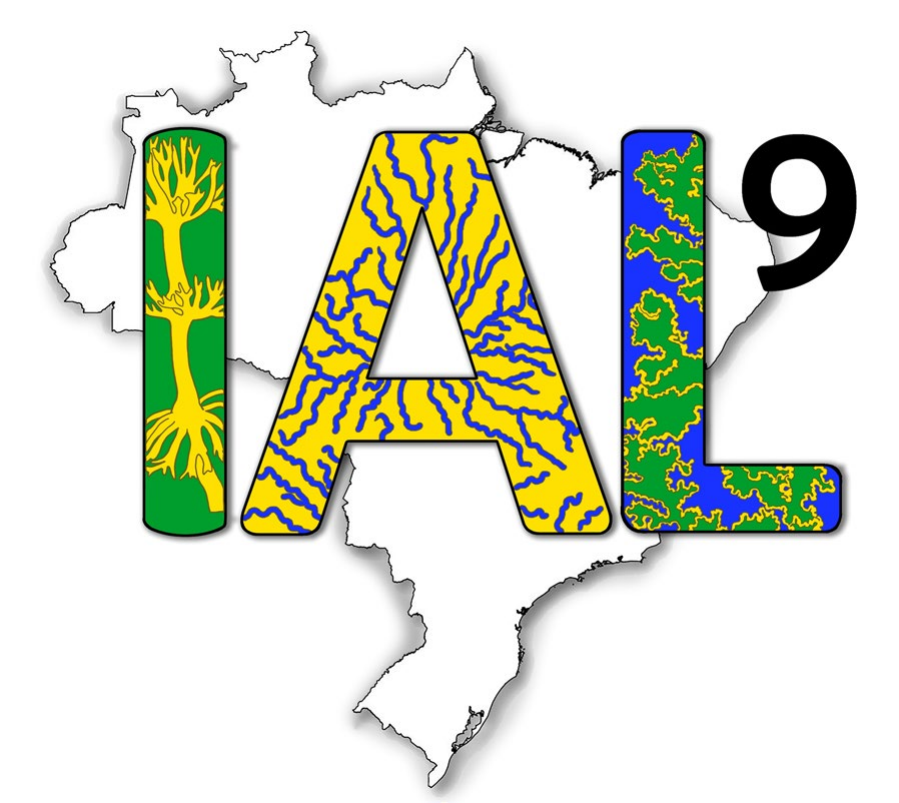
IAL9 logo

Up to 2016, there had been eight symposia of the International Association for Lichenology (IAL): six in Europe, one in North America, and one in Asia. The 2020 IAL meeting was the first scheduled to be held in South America, specifically, in Bonito, MS, Brazil. Then, in early 2020, with COVID-19 advancing, it became clear that IAL9 could not happen as planned. First, we thought that postponing the meeting one year would be enough to hold an in-person IAL9 in Brazil, but soon we realized that the only way would be to have an online conference. This was very disappointing, but on the positive side, this enabled many people to attend it that might not otherwise been able: there were 375 registered participants from 46 countries – a record in the history of IAL Symposia! We strived to have a more inclusive, diverse, and gender balanced meeting as possible, and there were a much greater number of female keynote speakers than in the earlier IALs. There were 12 symposia with a total of 60 talks: (1) Eco-evolutionary dynamics of symbiotic interactions; (2) Lichens and climate change: a multi-scale challenge; (3) Lichen-associated microorganisms; (4) Biodiversity of Neotropical lichens; (5) New approaches to harness genetic data from herbarium specimens; (6) Ecology, evolution and diversity of lichen algae; (7) Genomics and bioinformatic; (8) Macroevolution of lichens; (9) Lichens as invaders of rocks and control strategies; (10) New developments in the taxonomy of the *Lecanoraceae*; (11) Progress in classical and genomic aspects of lichen secondary chemistry; and (12) Paleotropical lichens.

**Fig. 12:**
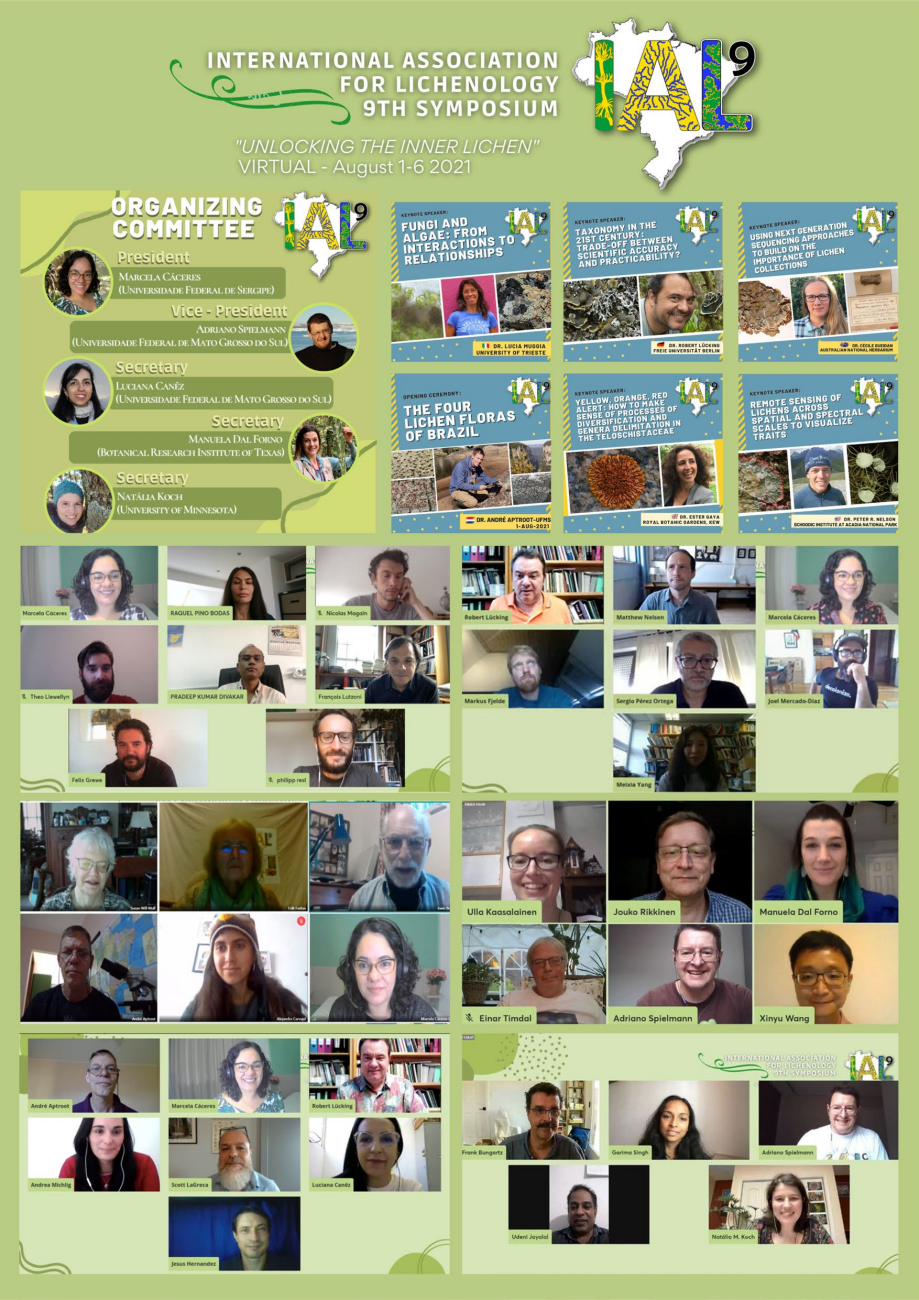
A flavour of IAL9

In addition, there were several topical sessions, which included 72 oral presentations, including lightning-talks, giving the opportunity to many young lichenologists to present their research for the first time in an international event. Additionally, we also had 141 posters presented and five fantastic workshops held by leading lichenologists in a variety of subjects. Figure 12 provides a flavour of this exciting first for the IAL!

We would like to express our most sincere gratitude to all who made the effort to collaborate and be part of the IAL9 online symposium. We also thank the IAL Council (2016–2021) and the Social Media and Scientific Committees for their invaluable services to our event and community. For more details, please https://doity.com.br/ial9/.

**Marcela E. S. Cáceres, Adriano Spielmann, Manuela Dal Forno, Luciana Canêz, and Natália Koch**

*IAL9 Organizing Committee*

(mscaceres@hotmail.com)

## MYCORISE UP!

(Fig. 13)

**Fig. 13:**
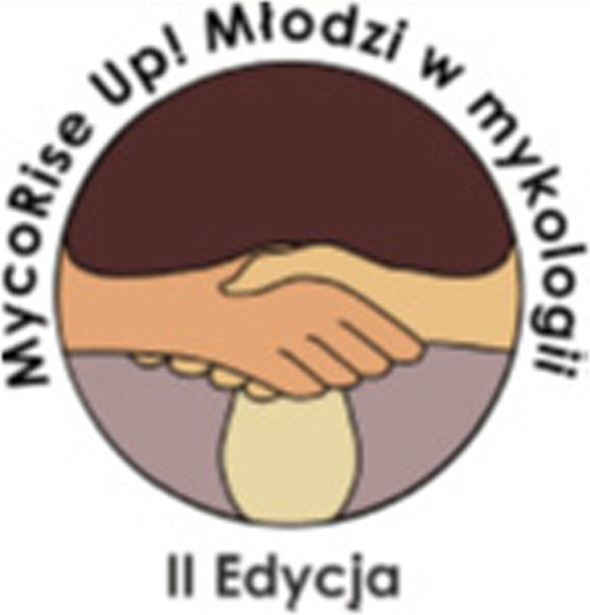
MycoRise Up! logo

The second MycoRise Up! meeting of young mycologists was held from 23–25 April 2021 in Poland. The event was co-organized by the Polish Mycological Society and the Institute of Plant Genetics of the Polish Academy of Sciences in Poznań (Poland), under the auspices of the Committee of Organismal Biology of the Polish Academy of Sciences, the Biodiversitatis Foundation, and the Complex of Landscape Parks of the Lodz Region. The conference was organized online on the ClickMeeting platform (technically operated by the Cyfrowy Dialog Association). The seven-person Organizing Committee was headed by Monika Urbaniak (Institute of Plant Genetics, PAS, Poznań), and the six-person Scientific Committee by Anna Biedunkiewicz (University of Warmia and Mazury in Olsztyn).

The meeting attracted 70 students from 22 research centers in Poland, one from India, and one from Peru. The plenary speech "Fascinating world of aeromycology" was delivered by Małgorzata Jędryczka (Institute of Plant Genetics, Polish Academy of Sciences in Poznań). The 49 students’ presentations and 13 multimedia posters were presented in six thematic sessions: (1) Fungal biology and ecology; (2) Medical mycology; (3) Fungal interactions; (4) Pathogenic fung; (5) Fungal genetics and genomics; and (6) Fungi in biotechnology. All participants were able to speak and ask questions via the "chat" option and, in addition, participants had special "virtual rooms" for backstage discussions open after each session. On the last day of the conference, Anna Biedunkiewicz presented a lecture "A guide for a young scientist – how to prepare for a conference speech". Finally, the Scientific Committee and the Organizing Committee awarded prizes for the best presentations and posters.

The book of abstracts and information on this and the preceding MycoRiseUp! conference are available at https://mycoriseup.wixsite.com/konferencja and the website of the Polish Mycological Society (http://www.ptmyk.pl/wp-content/uploads/2021/06/Mycoriseup2021_ksiazka_abstraktow.pdf). During the meeting, it was agreed that the next MycoRise Up! meeting in 2022 would be co-organized by students from the University of Warsaw. We look forward to seeing you there!

**Julia Pawłowska and Małgorzata Ruszkiewicz-Michalska**

(julia.z.pawlowska@uw.edu.pl)

## GERMAN MYCOLOGICAL SOCIETY (DGFM) CENTENARY

The German Mycological Society (DGfM) celebrated its centenary in Blaubeuren this autumn. Around 200 amateur mycologists and citizen scientists joined about 100 professional researchers from more than 20 countries in their biannual meeting. The meeting featured field trips, workshops, and scientific talks on various topics from ecology to genomics. The surroundings of the meeting venue offered several diverse habitats on limestone and claystone, featuring several rare fungi, and more than 500 different species were recorded.

**Fig. 14:**
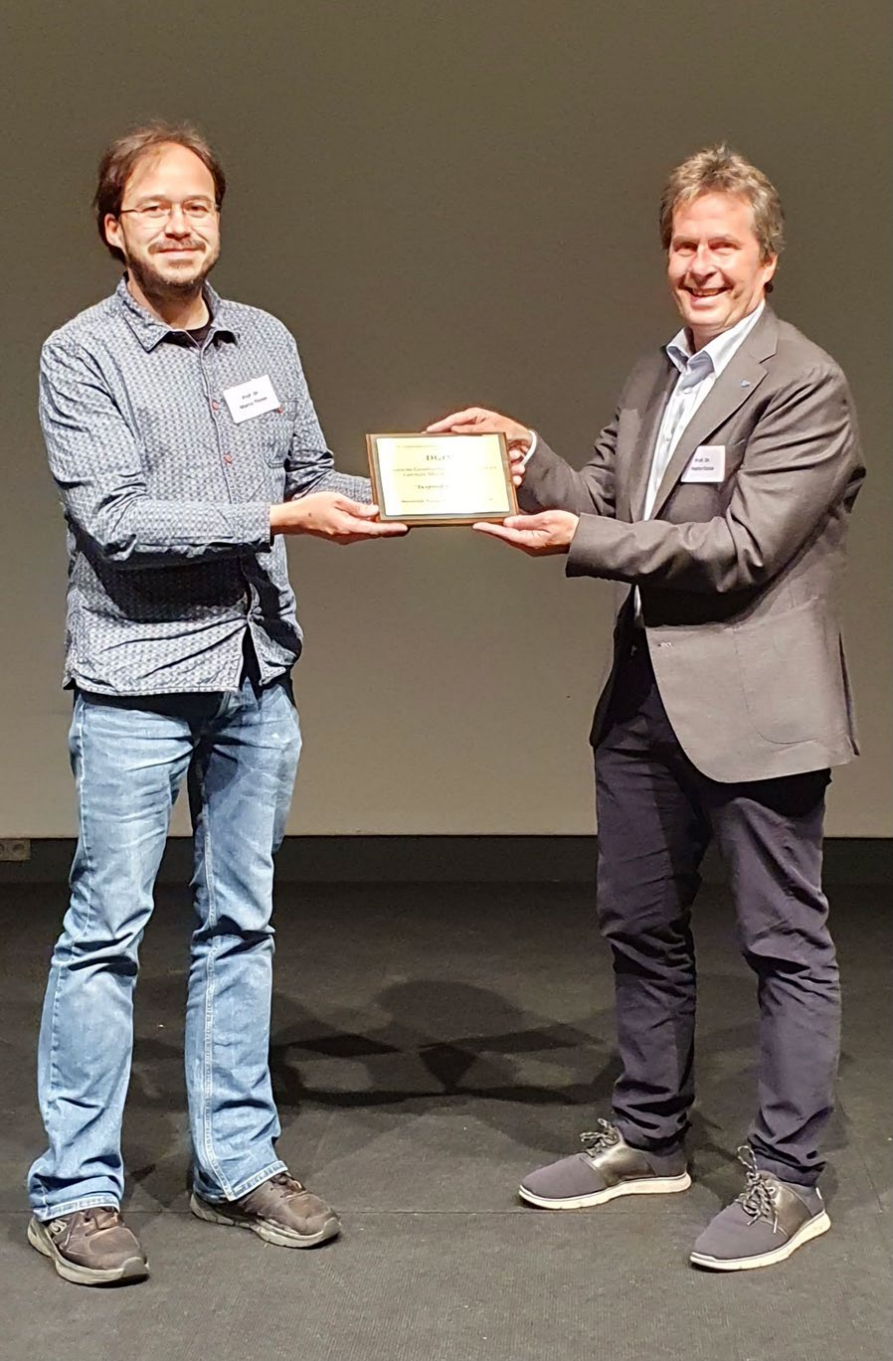
Marco Thines (*left*), President of the German Mycological Society, receiving a commemorative plate from the Westerdijk Biodiversity Institute presented by Pedro Crous (*right*)

Marco Thines, President of the German Mycological Society, received a commemorative plate from the Westerdijk Biodiversity Institute to mark the occasion (Fig. 14).

## BIRTHDAY GREETINGS

### David F. Farr – Initiator of major fungal databases

(Fig. 15)

**Fig. 15:**
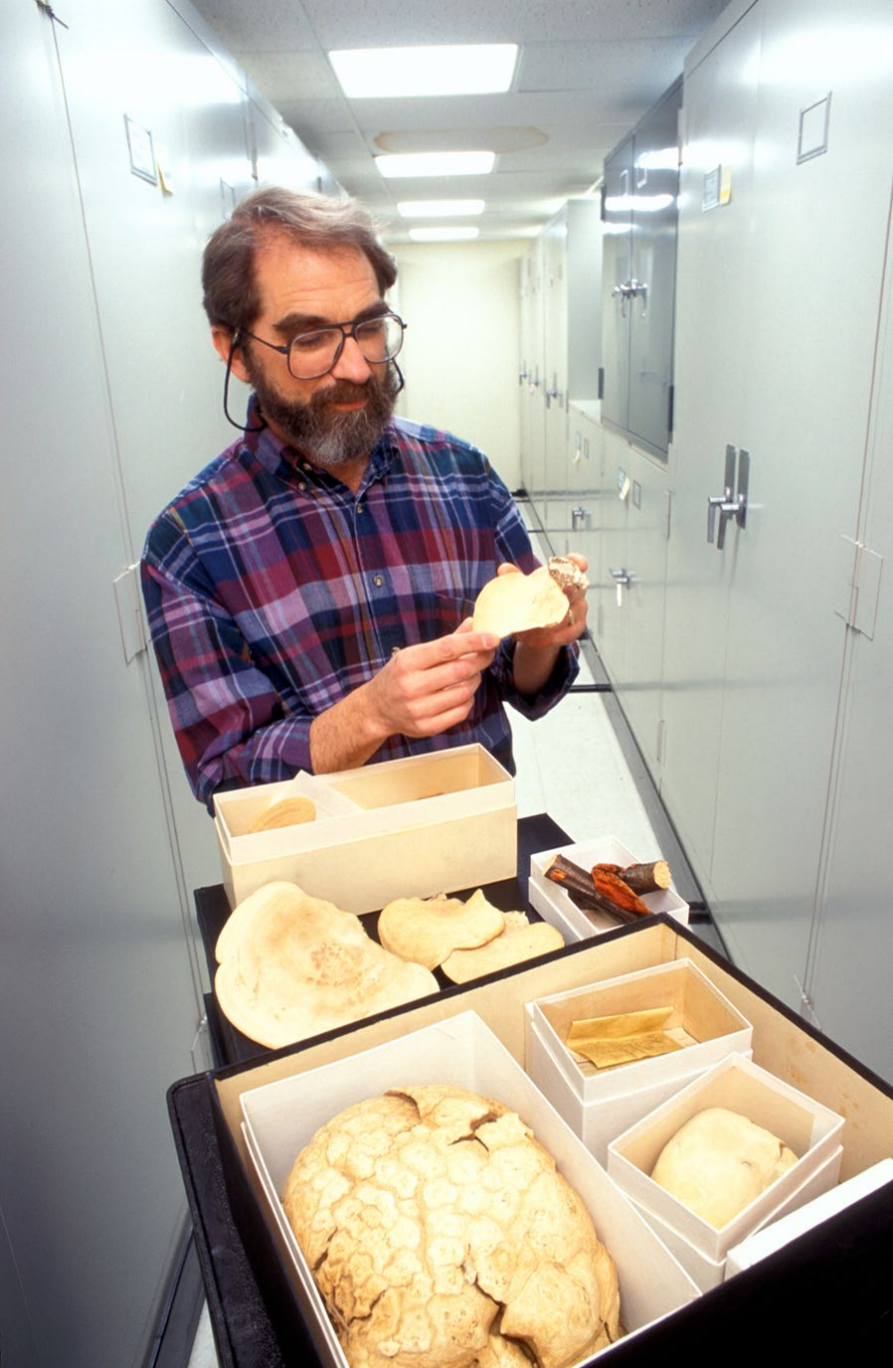
David F. Farr examining specimens in the US National Fungus Collection (BPI)

For most of his career from 1975 until his retirement in 2010, David, who turned 80 this year, worked for the USDA ARS Mycology Laboratory (and under its various names) in Beltsville, Maryland. There he contributed to systematic knowledge of plant-associated coelomycetes, especially *Septoria, Stagonospora,* and related genera publishing papers with exquisite microphotographs of their conidiomata and conidiogenous structures. For many years he also taught a course on mushrooms at the USDA Graduate School and served as an expert for local hospitals when mushroom poisonings were suspected.

David had the amazing foresight to develop several major database projects that have served as the foundation for incredible resources now available to mycologists worldwide. He initiated the first efforts in computerizing data associated with fungal specimens at the US National Fungus Collections in the 1970s using optical computer recognition sheets. Three decades later, nearly all 1,100,000 specimens had been entered into a database and are now combined with data from millions of other fungal specimens available at MycoPortal. He also created additional databases at the US National Fungus Collections, specifically accurate scientific names of plant-associated fungi, their reports on hosts throughout the world, and associated literature. These databases were initially developed for the production of the best-selling *Fungi on Plants and Plant Products in the United States* (Farr et al. 1989). After that project was completed, the USDA plant quarantine personnel asked if we might do this for the entire world; after we finished laughing and with funding, we embarked on such a project.

Dave masterminded the *Index to Saccardo’s* 26 volume *Sylloge Fungorum* (Reed and Farr 1993). He saw an unparalleled opportunity when Clyde Reed, a retired botanist-mycologist, walked into the Mycology Laboratory with tens of boxes of 3 × 5 ins index cards, which represented this Index. Having three cracker-jack data entry persons as a resource, Dave developed the program and directed them in entering the data for the 220,000 cards. Subsequently these records were given to the then International Mycological Institute (Kew) incorporated into *Index Fungorum,* now also used in *MycoBank,* and resulting in the comprehensive databases of all published scientific names of fungi so useful to everyone today.

Dave loves music, perhaps because his father was a music teacher, and for many years he and his wife Ellen have held season tickets to both the Baltimore Symphony Orchestra and the National Symphony in Washington DC. Conversations at the Laboratory often centred on which composers provided the most delight—Bach, Mozart or Mahler. After retirement, Dave and Ellen took up traveling the world and gourmet cooking, producing the most delicious and healthy dinners at their house—the best restaurant in town!

**Amy Y. Rossman and Lisa A. Castlebury**

(amydianer@yahoo.com)

### Marie-Agnès Letrouit-Galinou – Resolver of the position of lichen fungi in the *Ascomycota*

(Fig. 16)

**Fig. 16:**
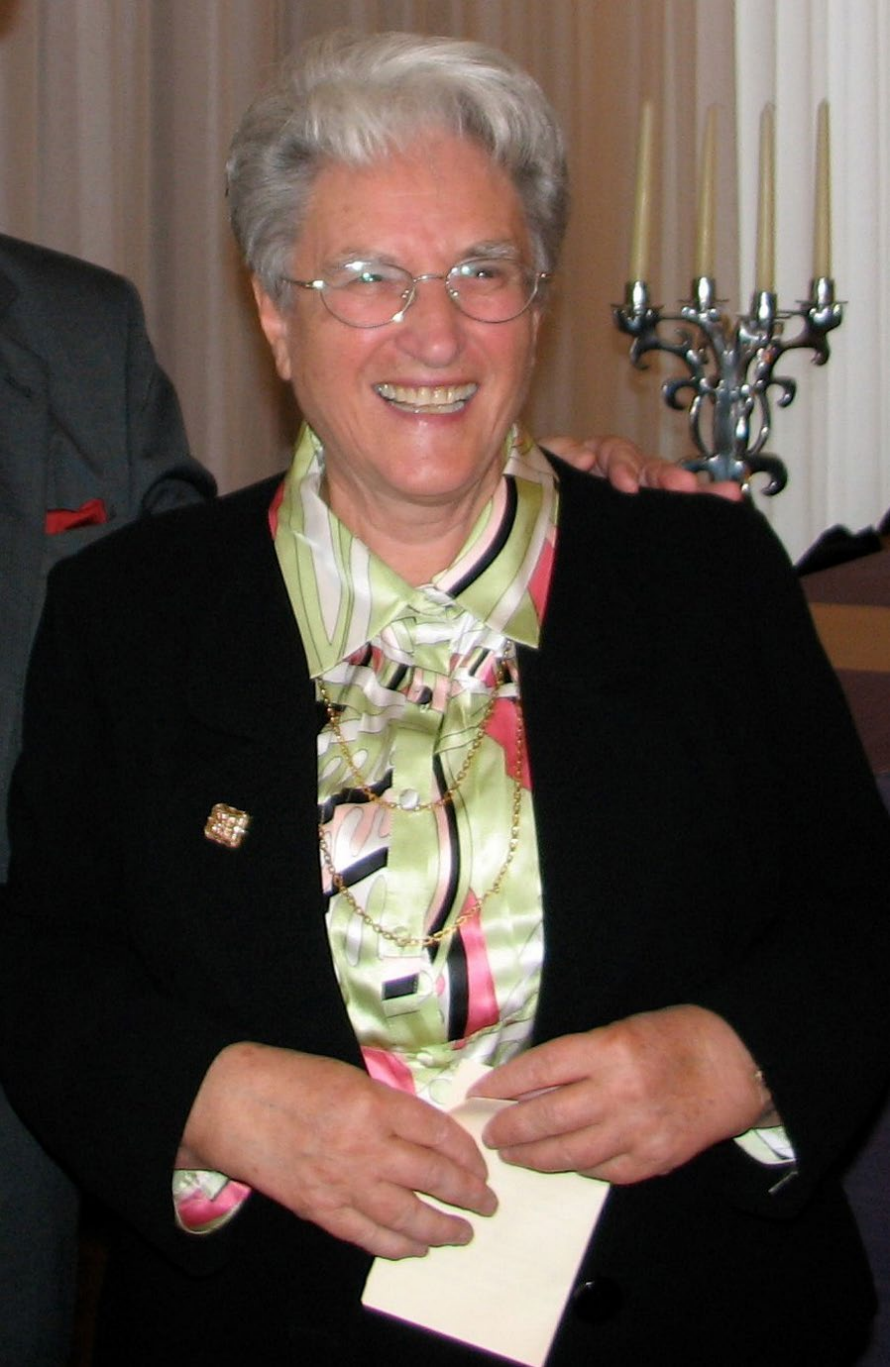
Marie-Agnès Letrouit-Galinou when awarded the Légion d'Honneur on 30 September 2008. Photo: J.Asta

Marie-Agnès Letrouit (born Galinou) was born in Mayenne (France) on 26 December 1931 and we join in sending her this tribute on the occasion of her 90th birthday.

After secondary studies at the boarding school Notre Dame of Mayenne and the high school of Rennes, she obtained her baccalaureate in 1948 and a degree in Natural Sciences from Faculty of Sciences of Rennes in 1951. Her scientific career at the CNRS (Centre National de la Recherche Scientifique) on lichenology, which spanned fifty years, began in the Botany laboratory of the Faculty of Sciences of Rennes under the direction of Professor Henry Des Abbayes. She was first appointed intern (1953) and then research associate (1956). She presented a Diploma of Higher Studies on *"The flora and vegetation of epiphytic lichens in the forest of Mayenne, followed by ecological, anatomical, cytological and physiological remarks on some species"* (1955). In 1958 she joined the laboratory of Marius Chadefaud of the Pierre and Marie Curie University of Paris and defended a doctoral thesis on: *"Research on anatomy and comparative ontogeny of the apothecia of some discolichens"* (1966).

**Fig. 17:**
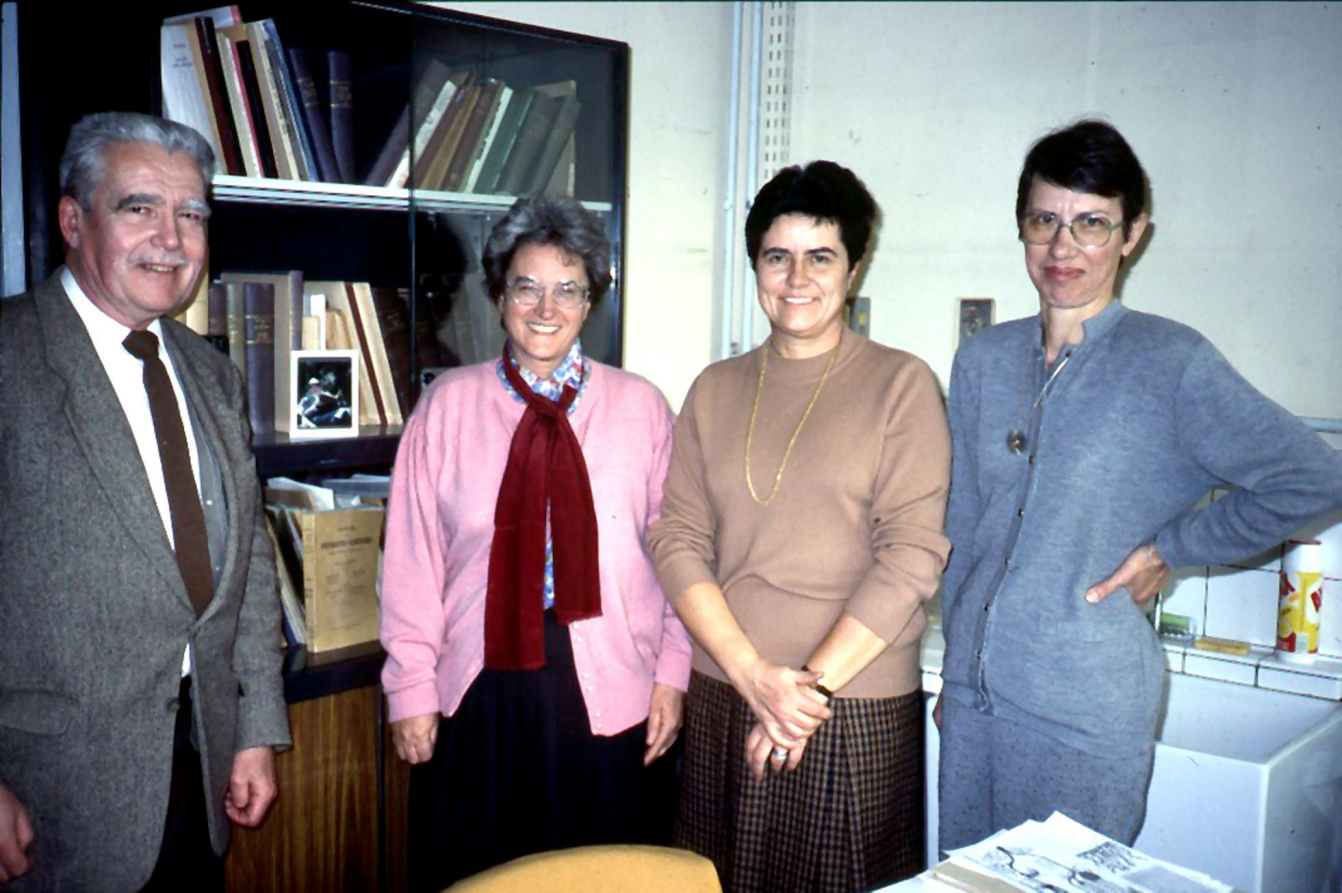
Chadefaud’s four PhD students (*left to right*): André Bellèmere, Marie-Agnès Letrouit-Galinou, Marie-Claude Janex-Favre, and Agnès Parguey-Leduc; Pierre and Marie Curie University, Paris 1989. Photo: D L Hawksworth

Marie-Agnès is recognized in the scientific world as a true pioneer**.** She has contributed to improving knowledge about asci, ascomata, thallus, and pycnidia of ascolichens and, consequently, *Ascomycota*. This first work, then conducted in collaboration with André Bellèmere, Marie-Claude Janex-Favre, and Agnès Parguey-Leduc (Fig. 17), showed that the previous views promulgated by J A Nannfeldt and E S Luttrell on *Ascomycota* ontogeny and ascus structure were erroneous. Subsequently, new hypotheses and a new classification, validated by electron microscopy studies and then by molecular data emerged. Her results proved to be largely authoritative.

She became Director of Research in 1982 and led the cryptogam team, which then included ten researchers in lichenology. During her career, she made several stays abroad that gave her the opportunity to create contacts with many lichenologists. She participated and/or successfully organized numerous lichenology congresses.

Marie-Agnès contributed greatly to the development of lichenology in France as a founding member of the *Association Française de Lichénologie* in 1976, which now has more than 300 members and promotes the training of young researchers in lichenology. Towards the end of her career, she became interested in air pollution and its impact on lichens. She helped to highlight the return of epiphytic lichens to the Jardin du Luxembourg in Paris following the drop in the level of SO_2_ in the air.

Her scientific influence was acknowledged by the presentation of the Gandoger Prize (Cryptogamy) of the Botanical Society of France in 1968, the Prize Allorge of the Academy of Sciences in 1993, the Medal of l’Association Française de Lichénologie in 2001, and the International Association of Lichenology Acharius Medal in 2004. The genus *Letrouitia,* which now contains about 15 species and the family *Letrouitiaceae* were circumscribed by André Bellemère and Josef Hafellner in 1982 and named in her honour.

Marie-Agnès is also a pioneer in the associative field related to public health issues. A difficult personal life led her to found, in 1986, the association *“Atharep",* for the working conditions of people with disabilities in public research. She is at the origin of the integration of non-ambulant people within the CNRS. In 1998, she created the association *"Schizo ?… Yes! Dealing with schizophrenia"* in which she still continues to work voluntarily on different aspects (administrative, research, patients' rights, help for families, etc.). Through this commitment, Marie-Agnès was named Chevalier de la Légion d'Honneur by the Ministry of Health in 2008.

Despite here honours, Marie-Agnès remains simple and modest. She is a great lady whose name is already inscribed in the history of lichenology but also in that of humanity! Thank you Marie-Agnès for this beautiful testimony of your life! We are proud to be your friends! Happy birthday Marie-Agnès!

**Juliette Asta and Chantal Van Haluwyn**

(juliette.asta@orange.fr; chantal.vanhaluwyn@orange.fr)

### Maria Olech – pionering polar ecologist and lichenologist

(Fig. 18)

**Fig. 18:**
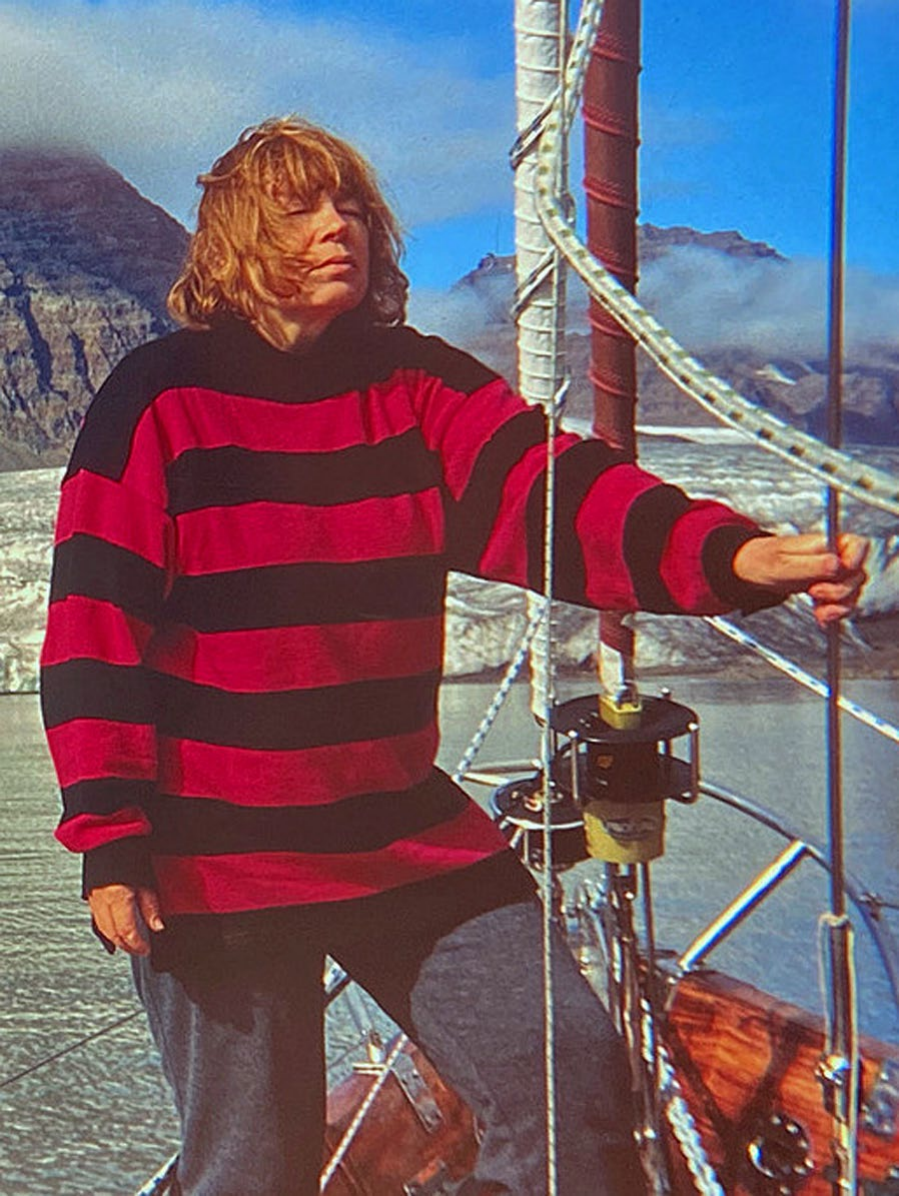
Maria Olech, off the east coast of Greenland. Photo: Marian Gorlikowski

Maria Olech celebrated her 80th birthday on 12 February 2021. She started her professional career at the Jagiellonian University in Kraków (Poland). She received a master's degree in 1963, in 1969 a doctorate in biological sciences, and in 1985 habilitation for research on “Lichen communities in high-mountain calcareous grasslands”. In 1992, she was conferred the title of professor. She worked continuously at the Jagiellonian University from 1968 to 2011, where she founded the Department of Polar Research and Documentation in the Institute of Botany in 1994 and remained its head until retirement. Her broad range of scientific activity was mostly devoted to polar issues, and she created the Polish school of polar botany. Maria participated in two expeditions to Spitsbergen (Svalbard) and a remarkable ten to the Antarctic. In 1997–1999, she circumnavigated all lands around the North Pole. She was the first woman to lead a year-long expedition to the Henryk Arctowski Polish Antarctic Station.

Her publications cover: functioning of tundra ecosystems, processes of spontaneous succession in the glacier forelands in the context of climate warming and deglaciation, synanthropization of the unique Antarctic biota, ecology of ornithocoprophilous lichens, adaptations of lichens to extreme environmental conditions. So far, she has published over 350 scientific articles and other works in which she described over 100 taxa of fungi (mostly lichenized and lichenicolous), mosses, and algae new to science. She was the co-author of the phytosociological map of Sørkapp Land (SW Spitsbergen) including descriptions of 28 tundra plant communities – the first study of this type for the Arctic region. Maria authored co-authored several books on polar lichenology, the most important being: *The lichen genus Caloplaca in polar regions* (1995), *Annotated checklist of Antarctic lichens and lichenicolous fungi* (2001), *Lichens of King George Island, Antarctica* (2004), and *Lichens and lichenicolous fungi of Schirmacher Oasis, Antarctica* (2010).

Maria actively participated in framework research programmes and often held key functions in organizations working in Antarctic environmental protection. She headed the team for biological monitoring of contamination of the Antarctic environment with heavy metals and radioactive elements, organized an international team for ultrastructural and molecular studies of Antarctic algae, and was involved in major international programmes such as BIOTAS, CS-EASIZ, and RSC. Further, she founded the Laboratory and Herbarium at the National Center for Antarctic and Ocean Research. Maria participated in the International Arctic Global Terrestrial Ecosystem group, was the SCAR Member of its Standing Scientific Group, a member of the Polar Research Committee of the Polish Academy of Sciences, the National Committee for the 4^th^ International Polar Year, etc.

In addition, Maria is a member of Editorial Advisory Board of the Polish Polar Research journal – and of the USA The Explorers Club – Word Center for Exploration.

*Ad multos annos!*

**Piotr Köhler and Piotr Osyczka**

(piotr.osyczka@uj.edu.pl)

### Angela Restrepo Moreno – A Life Devoted to Teaching Mycology

(Fig. 19)

**Fig. 19:**
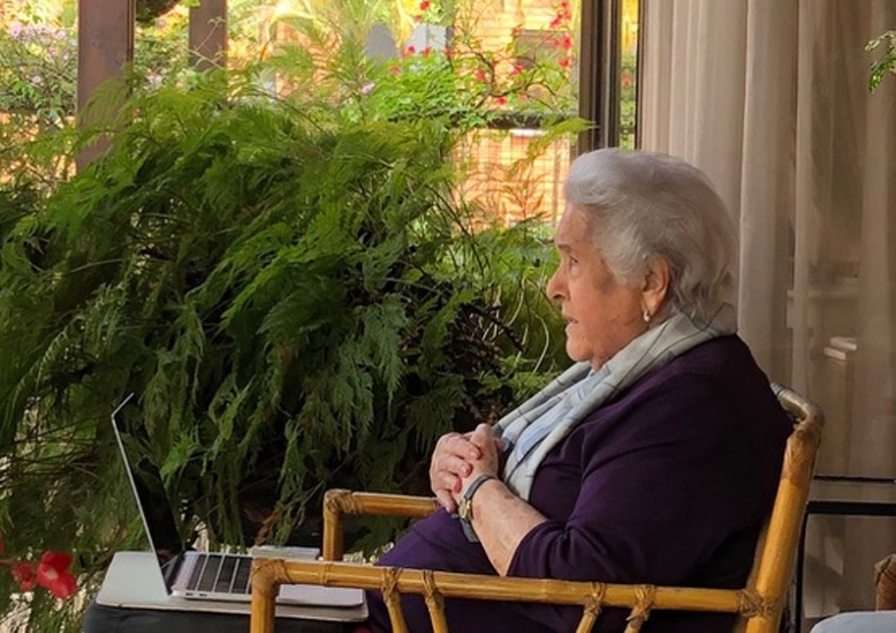
Angela Restrepo Moreno

Angela Restrepo Moreno has been a pioneer woman in Colombia. She decided to make science the axis of her life. Her contributions to knowledge, particularly in the field of mycology, and as a trainer of new scientists, has been a model for society and for the young minds that aspire to choose science as a life project.

Angela Restrepo was born in 1931, in Medellin. She graduated as a Clinical Laboratory Technician at the Colegio Mayor of Antioquia in 1955, and entered the bacteriology laboratory of the Faculty of Medicine of the University of Antioquia (UdeA) as a technician. Between 1958 and 1960, she did a Master of Science at Tulane University (New Orleans, USA). She returned to the UdeA to found the Mycology Laboratory, in the Department of Microbiology and Parasitology of the School of Medicine. Shortly after, she returned to Tulane University to obtain her PhD in 1965. She went back to the UdeA to consolidate the Mycology Laboratory as a national reference in diagnosis, research and teaching of diseases caused by fungi. Following her coerced retirement from the UdeA in 1976, she served as Scientific Director of the Corporation for Biological Investigation (CIB) from 1978 until her final retirement in 2015.

Her main contribution to the knowledge of fungal diseases, was Paracoccidiodomycosis and its causal agent *Paracoccidioides* spp., which is based on many decades of devoted study. She reached the summit of her scientific career during her years of work at the CIB. Leading the development of mycological, immunological, and molecular diagnostic methods, while being a pioneer in clinical trials with different medications for their treatment. Her research accomplishments are reflected in more than 250 publications, most of them in English, and over half of them as senior author. Additionally, she has been the author / co-author of over 40 book chapters. During her career, she had received multiple National and International awards (over 50), something she dislikes due to her unpretentious and humble personality. In spite of this for all of her disciples, she represents the model and standard of measurement to which all scientists should aspire to.

From the time she was a professor at the Faculty of Medicine of the UdeA, Angela Restrepo has trained young students of mycology and has supported other related disciplines. She gathered around her a large number of brilliant young pupils who were trained as scientists under her tutelage. Her students have spread to multiple institutions both in Colombia and around the world, always bearing the indelible mark of having been her disciples. In her honor, several of her students chose to name one of the new *Paracoccidioides* species using her last-name, *Paracoccidioides restrepiensis*. Angela Restrepo (la doctora, as we called her) chose science as the center of her life and did not form a classic family. However, she considers that she has hundreds of children thanks to SCIENCE.

**Juan G. Mc Ewen**

mcewen@une.net.co

### Carol Ann Shearer – a doyen of aquatic ascomycetes

(Fig. 20)

**Fig. 20:**
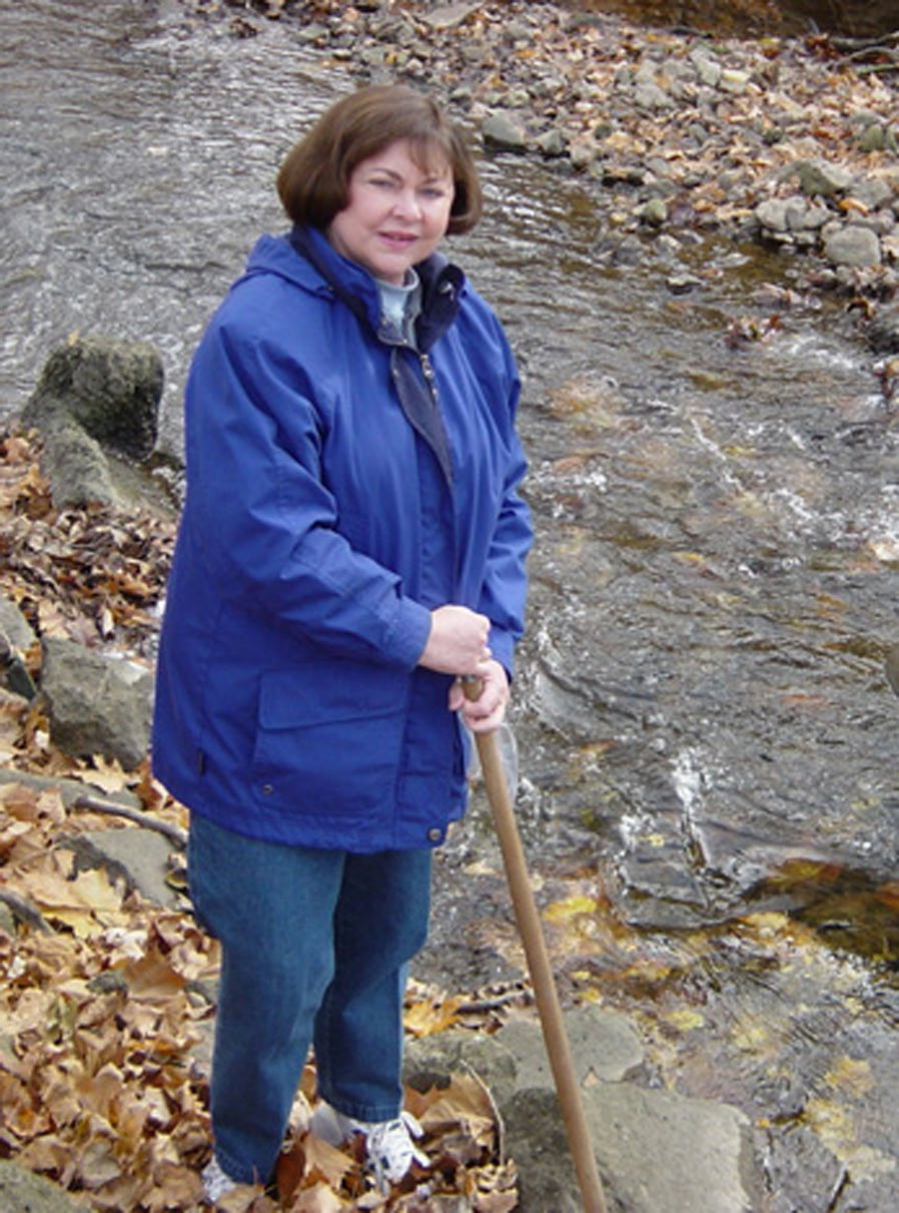
Carol A Shearer. Photo: Walter L. Hurley

Carol Shearer celebrated her 80^th^ birthday in March this year. Carol is a leading authority on aquatic ascomycetes whose academic contributions span more than 40 years. Carol earned her PhD in 1971 from the University of Maryland and joined the Department of Plant Biology at the University of Illinois at Urbana-Champaign (UIUC) in 1973. She was one of only five women hired as assistant professors at UIUC that year and was a pioneering woman in her department. Carol rose to the rank of full Professor in 1989 and was the head of the Department of Plant Biology from 1992 to 1996. She was also Curator of Mycological Herbarium at UIUC from 1986 until she officially retired as Professor Emerita in 2007. Carol remained active in research and mentorship following retirement, graduating her final PhD student in 2015. During her career, Carol published more than 150 papers on aquatic ascomycetes. Her work mainly focused on the ecology of freshwater saprophytic fungal communities, systematics, and biogeography of freshwater ascomycetes and and their asexual morphs.

Carol was committed to serving the mycological community. She was Vice-President of the Mycological Society of America (MSA) for 2001–2002, President-elect 2002–2003, and was one of the few women Presidents of the MSA from 2003 to 2004. Carol has also served on the editorial board of prestigious mycology journals, including *Mycologia* (1981–1989), *Mycological Research* (1998–2000), and *Mycotaxon* (1999–2007). Carol was awarded over US$ 2 million in funding from the National Science Foundation (NSF) and National Institutes of Health (NIH). In recognition of their contributions to the field, a special issue of *Mycological Progress* was recently dedicated to Carol, John Webster, and Terence Ingold (Baschien and Hyde 2018).

Carol has been a leading figure in the study of freshwater ascomycetes in the USA and globally. Her early research focused on the distribution and taxonomy of lignicolous ascomycetes of the Chesapeake Bay and its tributaries (Shearer 1972). Her seminal review on the freshwater ascomycetes (Shearer 1993) has been cited more than 200 times and has inspired numerous ecological and taxonomic studies of freshwater ascomycetes worldwide. Overall, she has described over 100 taxa of freshwater ascomycetes from the USA and the neotropics. In the mid-1990s, she was among the first to embrace the application of molecular sequence data to determine the phylogenetic relationships amongst freshwater ascomycetes. Her research on freshwater ascomycetes led to the separation of numerous taxa previously lumped under terrestrial taxa and led to discovering exclusively freshwater clades (order and families) of freshwater ascomycetes, such as *Minutisphaerales* and *Natipusillales*. It has also led to numerous new singleton genera, which are likely to represent new freshwater clades as new fungal species are discovered. Carol was also a leader in embracing online open databases for the dissemination of knowledge. She created a comprehensive online database for freshwater ascomycetes in the early 2000s, which continues to be a valuable resource t (http://fungi.life.illinois.edu); it has been used more than 4 600 times in the last year alone!

In addition to her interests in taxonomy and phylogenetics, Carol strove to identify patterns and factors underlying the spatio-temporal distributions of fungi in diverse aquatic habitats and across spatial scales. Carol recognized that micro-scale interactions between a fungus and its substrate and among fungi on a substrate, as well as macro-ecological patterns, must be understood to make progress in this area. Together with her students and colleagues, Carol worked to identify the enzymatic capacities of diverse aquatic fungi and to study their competitive interactions. From her work, we gained knowledge about aquatic fungal communities and biodiversity at scales ranging from individual leaves to rivers (e.g. Patuxent River, Sangamon River, and the River Teign), to regions (Chesapeake Bay and tributaries), to ecosystems (mangroves and temperate lakes) and at global scales.

For over three decades, Carol systematically collected and isolated freshwater ascomycetes and their sexual morphs by taking a latitudinal approach. Her work on species distribution patterns of freshwater ascomycetes suggests species composition varies along latitudinal gradients, with the most significant differences in species composition occurring between sites at opposite ends of the gradient. Her work in the neotropics in Peru along an altitudinal gradient revealed different structuring of freshwater fungal communities at low, middle, and high elevations, indicating a change in species composition along the Andes to Amazon elevational gradient (Shearer et al. 2015).

During this time, Carol also screened these cultures for secondary metabolite chemistry in collaboration with her colleague Jim Gloer at the University of Iowa. Together, this collaboration led to the discovery of numerous new compounds with interesting biological activities. One freshwater ascomycete strain also led to the isolation of 14 chemical compounds new to science. Her studies on examining the chemical diversity of freshwater ascomycetes has led to other groups becoming interested in screening freshwater fungi for novel chemistry. Today, aquatic fungi are specially targeted in global bioprospecting efforts and screened for biomedical and industrial potential. Carol and Jim's work was at the leading edge of this "hot" field.

Carol is a talented scientist who loves the beauty and diversity of freshwater ascomycetes and has instilled the same enthusiasm in her students. Since her retirement, she has spent her time with her husband in their Champaign, Illinois, home. Carol enjoys reading books and watching TV. She loves watching figure skating competitions and spending time at the beach during the summers.

Carol taught her students to write by encouraging us to "say what you mean and mean what you say". Here we do both. We thank Carol for sharing her knowledge and passion for aquatic fungi with us (her students), and the world. We wish Carol all the best in her 80th year and beyond.

**Huzefa A. Raja and Jennifer L. Anderson**

(haraja@ung.edu; jennifer.anderson@ebc.uu.se)

### James “Jim” M. Trappe –– placing truffles centre stage

(Fig. 21)

**Fig. 21:**
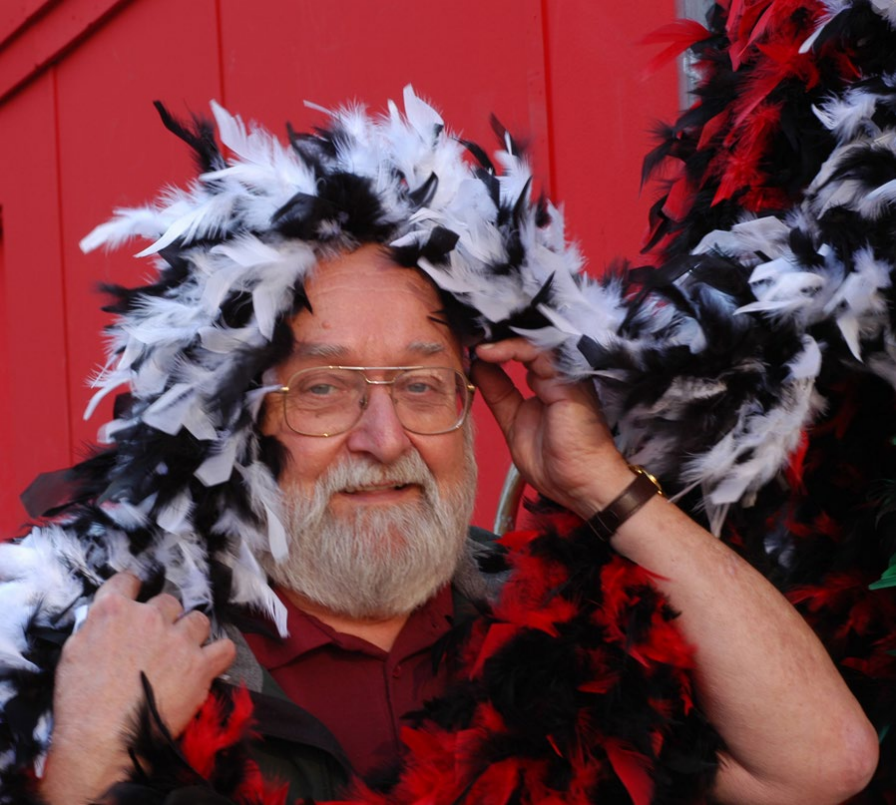
”Jim” and Trappe having fun, Plaza de Major, Madrid, 2007

In mid-August of this year, James “Jim” M. Trappe reached the grand age of 90 years – no mean feat for one that has maintained such a busy professional life over so many decades and travelled extensively (and almost constantly) around the world over that period. He is an exemplar of someone with a deep passion for their subject, always willing to go the extra yard in pursuit of excellence.

A native of Spokane in Washington State in the Pacific Northwest, Jim developed a keen interest for the outdoors and forests from a very young age. This was pursued with a BSc in Forest Management at the University of Washington, culminating in a PhD in the late 1950s on *Cenoccum graniforme,* mentored by Daniel Elliot Stuntz. While still conducting his PhD research Jim started work with the USDA Forest Service Pacific Northwest Research Station, originally at La Grande, Oregon, then later at Portland and finally Corvallis. He was appointed Project Leader in Forest Mycology at the Corvallis station in 1965 and remained there until retiring from the organisation in 1996. During his tenure he oversaw a large team of scientists working on various aspects of forest management, particularly as it related to the role of fungi in those ecosystems. In order to better understand that role, the taxonomy of those fungi became a key and lifelong focus for Jim, extending to the present day.

Commencing in the 1990s, Jim became fascinated with Australia, particularly its unique and incredibly diversity of native truffles. Together with colleagues from Corvallis, his students, and collaborators from around Australia, he was responsible for collecting many thousands of specimens from across that continent. From these collections, Jim and his collaborators went on to describe hundreds of new species, providing for the first time a taxonomic foundation for a hitherto extremely poorly understood group of organisms.

Associated with this primary research Jim, as always, keenly pursued related subject matter and extension activities, including but not limited to: (1) exploring interrelationships among truffles and mycophagous (fungus-feeding) mammals; (2) advising government land management agencies on forest and woodland restoration – as it related to mycorrhizal fungi and recovering threatened marsupials; and (3) training people in methods for surveying truffles in woodland landscapes. As a well-practised professional at the top of his game, Jim has always been in demand across such fora and is deeply respected.

His exploits in surveying and describing truffles are by no means confined to the Pacific Northwest and Australia, and he has documented similar fungi in forests from most parts of the world, including Asia, Europe, and South America. An extensive list of collaborators and students have been mentored along the way. He has authored or co-authored > 400 species of fungi, mostly truffles. Associated with his taxonomic work and related matters around the ecology and function of truffles, Jim has been involved in publishing > 450 scientific documents so far. Further, he has co-authored three books, including a *Field Guide to North American Truffles: hunting, identifying, and enjoying the World’s most prized fungi* (with his son Matt Trappe and Frank Evans; Trappe et al. 2007), and *Trees Truffles and Beasts: how forests function* (with Chris Maser and myself; Maser et al. 2008, reprinted three times).

Never one to stop, Jim has been publishing consistently and productively since the early 1960s – and shows no sign of stopping. Part of the key to this productivity undoubtedly relates to his love of life and people, including jokes to make you smile, and having found his passion early and working hard ever since. Along the way an equal love of good food, beer, wine and the occasional gin and tonic clearly hasn’t hurt either.

The IMA wishes him all the best, and trusts he will he continue to enjoy life and fight for an increased appreciation of the role of mycorrhizal fungi, especially truffles, in forest ecosystems.

**Andrew W. Claridge**

(andrew.claridge@dpi.nsw.gov.au)

### Shun-ichi Udagawa – pioneering explorer of ascomycetes in culture

(Fig. 22)

**Fig. 22:**
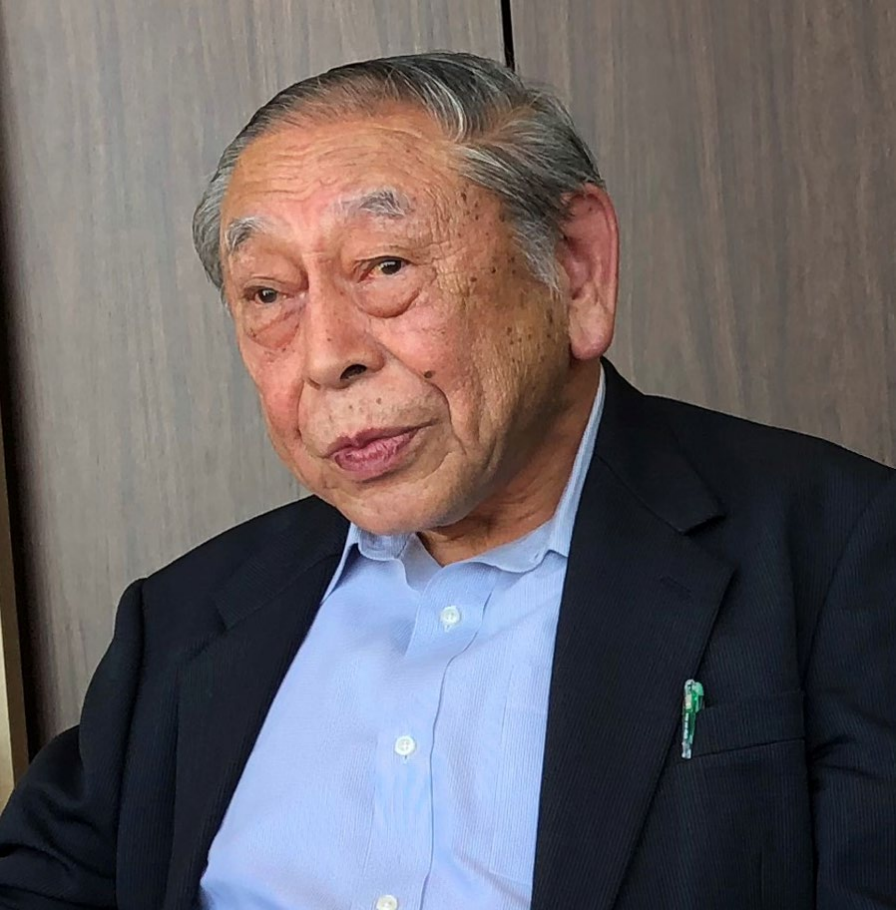
Shun-ichi Udagawa in a restaurant, Tokyo, Japan, November 2018. Photo: T. Yaguchi

Born and raised in Tokyo, Shun-ichi Udagawa celebrated his 90th birthday (“Sotsuju” in Japanese) on 31 March 2021. After graduating from the Faculty of Agriculture (Agricultural Chemistry), Tokyo University of Agriculture (TUA) in 1953, he worked as a mould researcher for 38 years at the National Institute of Hygienic Sciences (NIHS) in Tokyo (now the National Institute of Health Sciences in Kawasaki), of which 18 were as a section head. In the 1950s–1960s he was involved in detection and identification of “yellow rice”-related mycotoxigenic fungi from imported rice (Udagawa and Tatsuno 2004). During this period, in 1965–1966 he was an overseas researcher working on coprophilous fungi in Roy Cain’s laboratory at the University of Toronto, and in 1968 earned his doctoral degree from the TUA with a thesis entitled *Studies of* Eurotiales *and related fungi from Japan*. In the 1970s–1980s he was involved in a project on the making of GMP for pharmaceuticals and then the code of food hygiene practices. He was also committed to nurturing younger researchers in ascomycete taxonomy. After retirement from the NIHS in 1991, while continuing his research mainly at the Japan Food Research Laboratories (until 2011), he served as an adjunct professor/lecturer at several universities.

His research focuses on not only taxonomy on the cleistothecial and perithecial ascomycetes, but also food mycology and medical mycology. For fungal isolates obtained widely from nature (soil, animal dung, etc.) to foodstuffs and living environment, he has undertaken diverse research themes with accurate methodologies, precise light microscopic and SEM investigations, insightful analysis, and continuous passion. His research achievements have been reflected in 365 scientific papers, including 280 original articles in English with roughly 120 as senior author, and about single-/co-edited 80 books (including single-/co-authored book chapters) in Japanese/English. Incidentally, his (with collaborators) six new genera (e.g. *Acanthogymnomyces*, *Calceomyces*, *Gymnostellatospora*) and 154 new species have been re-described and illustrated in the *Atlas of Soil Ascomycetes* (Guarro et al. 2012), and similarly many of his new species for *Aspergillus*, *Penicillium,* and *Talaromyces* have been accepted under the single name nomenclature (https://www.aspergilluspenicillium.org/species).

He served as Editor-in-Chief of the *Transactions* (1979–1981) and President (1989–1990) of the Mycological Society of Japan (MSJ), and a member of both the International Commission on the Taxonomy of Fungi (ICTF) and International Commission for Mycotoxicology (ICM). In 1997 he was honoured as the first recipient of the MSJ Award (Udagawa 1997) and in 2004 shared the Hiratsuka (Paper) Award with collaborator also from the MSJ. During 1998–2006, in light of his outstanding contributions to mycology and related disciplines, he was elected an honorary member by the MSJ, Japanese Society for Medical Mycology, Japanese Society for Food Microbiology, Japanese Society for Mycotoxicology, and Japanese Society for Food Chemistry.

The IMA wishes Udagawa-sensei a very Happy Birthday and all the best for the future.

**Junta Sugiyama and Takashi Yaguchi**

(jsugiyam@tecsrg.co.jp; yaguchi@chiba-u.jp)

## IN MEMORIAM

### Heinz Butin (1928–2021)

(Fig. 23).

**Fig. 23:**
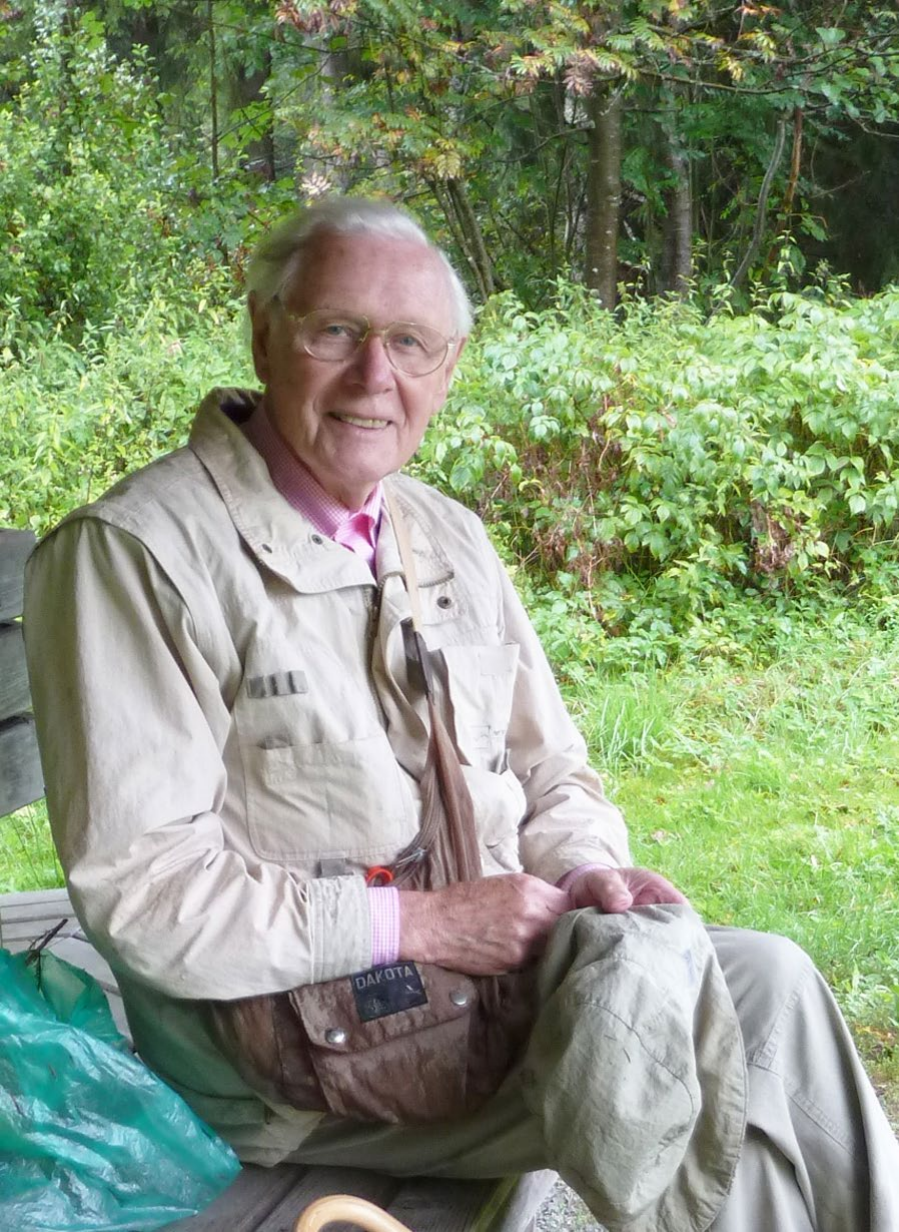
Heinz Butin. Photo: Bärbel Butin

Heinz Butin was born in Bad Godesberg on 13 April 1928, and passed away in Wolfenbüttel on 28 February 2021, aged 92 years. He completed his PhD in 1954, with a dissertation on the relation of water management and photosynthesis in lichens, at the Faculty of Mathematics and Natural Sciences at the University of Bonn. He then moved to the Institute for Forest Plant Diseases, led by Herbert Zycha, working on forest pathology, particularly forest mycology. In 1969, Heinz was appointed Scientific Director of that Institute, and the forestry faculty of the University of Göttingen made him an honorary professor. In 1976 he became Director and professor at the Institute for Agriculture and Forestry in Braunschweig.

Together with Zycha, Butin published the textbook *Forstpathologie* (Butin and Zycha 1973) which included many original observations, for example some previously unknown asexual morphs of known fungi. His most successful book, however, which made him internationally known, was on diseases of forest and park trees, first published in German in 1983 (Butin 1983), then translated into English (Butin 1995), and with five editions to 2019.

In the course of the debate on the so-called "forest deaths", together with Günter Hartmann and Franz Nienhaus, he wrote another book on the diagnosis of tree diseases (Hartmann et al. 1988). The book was not only widely used by forest students and practitioners in this field, but also by other nature enthusiasts concerned about forest health. The work has also been translated into several other languages, and in 1992 the *Colour Atlas of Wood Diseases: Ornamental Shrubs and Park Trees* was published (Niehnaus et al. 1992), which Butin co-authored with Franz Nienhaus and Bernd Böhmer. In addition, Butin has published over 200 papers in national and international phytopathological journals, and described 60 new species, mostly ascomycetes.

He is one of those too rare researchers that also has a passion for educating and assisting others by producing informative books on their identification. He was a most critical observer, something that was brought home to me when we corresponded after he discovered the sexual morph of *Polymorphum rugosum* (Butin 1977), and our thoughts are with his widow Bärbel Butin at this time.

I am indebted to Marco Thines for translating a German biography of Heinz on which this entry is partly based, and to his widow for the portrait included here.

### Karl Esser (1924–2019)

(Fig. 24)

**Fig. 24:**
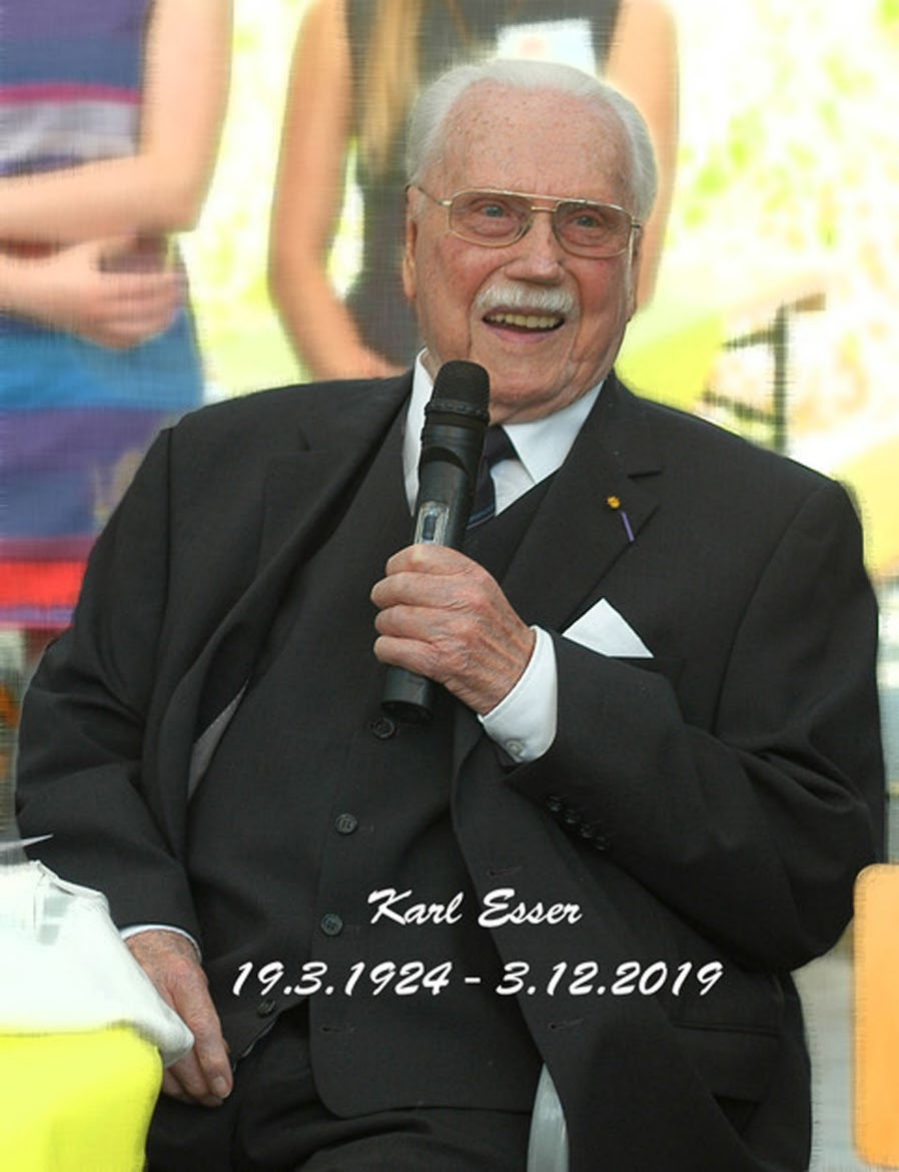
Karl Esser. Photo: Ulrich Kück

It is with deep regret that we record the death of Karl Esser, an Honorary President of the International Mycological Association (IMA), on 3 December 2019 at the age of 95 years. During the 22 years 1967–1989, he was head of the department of General Botany as well as director of the Botanical Garden at the Ruhr University Bochum, Germany. He was an internationally recognized and respected scientist working on many aspects of fungal biology and genetics.

Karl Esser studied at the Cologne University and graduated with a PhD in biology and biochemistry in 1952. He was research fellow at the Genetic Institute in Paris, at the Botanical Institute in Cologne, and at the Department of Microbiology at Yale University, New Haven, Connecticut, USA, before he became professor with the chair of General Botany in Bochum. He was a founding member of the newly established university and was nominated as first dean of the faculty of biology. In this function, he not only established one of the largest chairs focusing on fungal biology in Germany and founded one of the largest botanical gardens in Germany, but also developed a modern faculty of biology which became home to thousands of students for more than 50 years so far. He retired in 1989, but was actively working for mycology almost until his last days.

He dedicated his scientific career to fungal genetics, and not only established DNA transformation in filamentous fungi as one of the first groups, but also was highly influential in two basic aspects of biology. The studies of perithecium development in the ascomycete *Sordaria macorspora* are contributing not only to our understanding of sexual development in fungi, but also to our understanding of genetic principals in all eukaryotes. The research on senescence in *Podospora anserine* involved many general aspects of aging for which he was awarded the SANDOZ Prize for Gerontological Research, due to its relevance for aging in humans. Beside many other distinctions and prizes, he became docteur *honoris causa* from the Université d’Orleans, Université de Toulouse, and Université de Lille, all in France.

Beside his scientific impact he was highly influential in international biology and especially mycology. He was involved with the International Union of Biological Sciences (IUBS) for many years, served as chairman of the International Union of Microbiological Societies (IUMS), president of the 14th International Botanical Congress in Berlin in 1987, and was a founding member of the IMA at the 1^st^ International Mycological Congress (IMC1) in Exeter in 1971. He worked continuously on exchanges between scientists and supported IMA from the early days of international mycology. He was member of the Executive Committee and vice-president of the IMA for many years, and was supportive for several IMCs worldwide. In 1990, on the occasion IMC4 in Regensburg, he was made an Honorary President.

Karl Esser was a great and enthusiastic teacher of mycology. Since I started in Bochum in 2007, long after his retirement, I remember several discussions with him on teaching mycology as an essential part of biology. He wrote several important textbooks, including *Genetics of Fungi* (Esser and Kuenen 1967) or the German textbook *Kryptogamen* (Esser 1976) which was later translated and published by Cambridge University Press in 1982 (also subsequently divided into two volumes). He was editor of the encyclopaedic multivolume series *Mycota,* which remains the most influential book series in mycology. I have no doubt that his students, including Frank Kempken, Ulrich Kück, Friedhelm Meinhardt, Heinz-Dieter Osiewacz or Paul Tudzynski and in turn their students, will carry on his research on fungal genetics to improve our understanding of key organisms in Earth’s ecology, which are highly relevant in applied sciences that can contribute to safeguarding the future of our planet.

I am highly grateful to Ulrich Kück for many details of the CV of Karl Esser and the courtesy to provide a photograph of him from his private collection.

**Dominik Begerow**

(dominik.begerow@rub.de)

### Grégoire Laurent Hennebert (1929–2021)

(Fig. 25)

**Fig. 25:**
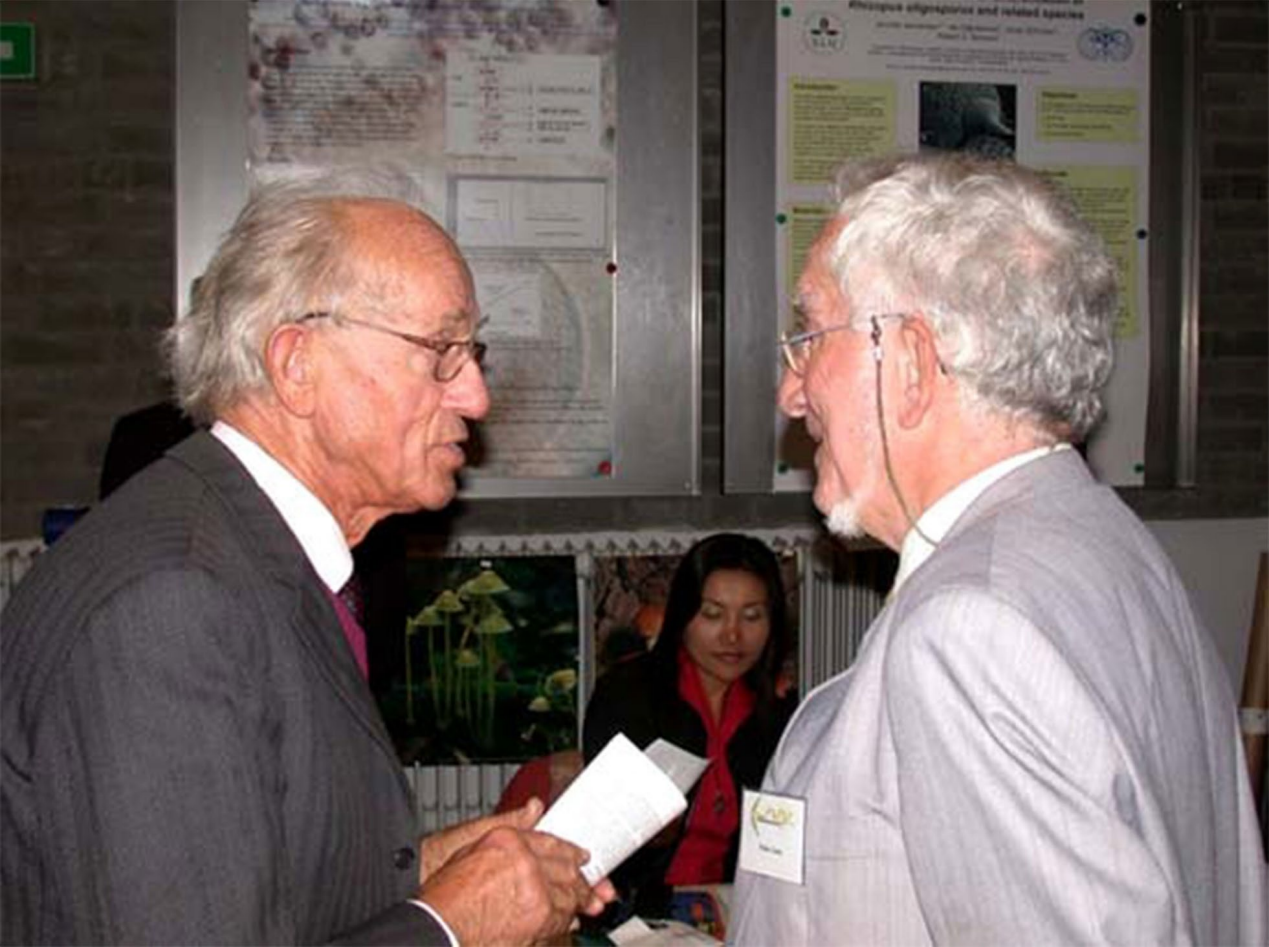
Grégoire Hennebert (*left*) and Walter Gams (*right*) chatting at the centenary symposium of the Centraalbureau voor Schimmelcultures (now Westerdijk Fungal Biodiversity Institute) in Amsterdam, 2004. Photo: Pedro Crous

It is with great regret that we must record Grégoire’s passing in Ottignies-Louvain-la-Neuve, Belgium, on 13 October 2021 at the age of 92. He was born in Mons, Belgium on 20 June 1929, and his career and key achievements were summarized in *IMA Fungus* on the occasion of his 90^th^ birthday (Hawksworth 2019) and so are not repeated again here. He has also provided what he termed a personal “life-path” of his formative development in mycology through his visits and correspondence with Stanley J. Hughes in Ottawa, initiated in 1959, and which continued almost to Stan’s death in 2019 (Hennebert 2020a).

He had a particularly philosophical and lively mind, and in addition to his scientific contributions, he is well-known for introducing, along with Luella K. Weresub (who he had also met in Ottawa) in 1977, the anamorph/teleomorph/holomorph terminology into mycology. He was always willing to challenge established ideas, often with a mischievous smile, and with reasons that made one think. It was, however, gratifying to see how he came to openly accept the demise of the use of the “-morph” terms when the separate naming of morphs of pleomorphic fungi was abandoned in 2011. This is evidenced in his last publication, which came out only last year, where he revisited and revised some of his previous taxonomic and nomenclatural conclusions (Hennebert 2020b).

It also important to stress his devotion to encouraging mycology in the French-speaking countries of Africa, especially Burundi and Rwanda, through training courses, publications, compilations of mycologists, and conferences—the report of the last issued online and published as a few hard copies at his personal expense (Hennebert 2010).

Grégoire also made a great contribution to mycology worldwide through establishing in 1974, along with Richard P. Korf, the journal *Mycotaxon* to provide an outlet for papers on the taxonomy of fungi, and originally produced from camera-ready copy prepared by the authors. *Mycotaxon* continues to thrive, and I understand that a detailed tribute is to be provided in that journal next year.

He always kept himself very fit with regular swims, and could be relied on participating in key mycological meetings when well into his 80s. Grégoire also confided he partook *Ophiocordyceps sinensis*.… In May of this year, however, he had to undergo complex heart surgery, after which he commented to me that he “overlived fortunately and [was] very happy to sheer [*sic*!] life and the family much longer”. Indeed, he was very much a family man, missed his wife Lidwina who pre-deceased him in 2009, but was fortunately supported by a loving extended family. Our thoughts are with them at this difficult time.

### Jack D. Rogers (1937–2021)

(Fig. 26)

**Fig. 26:**
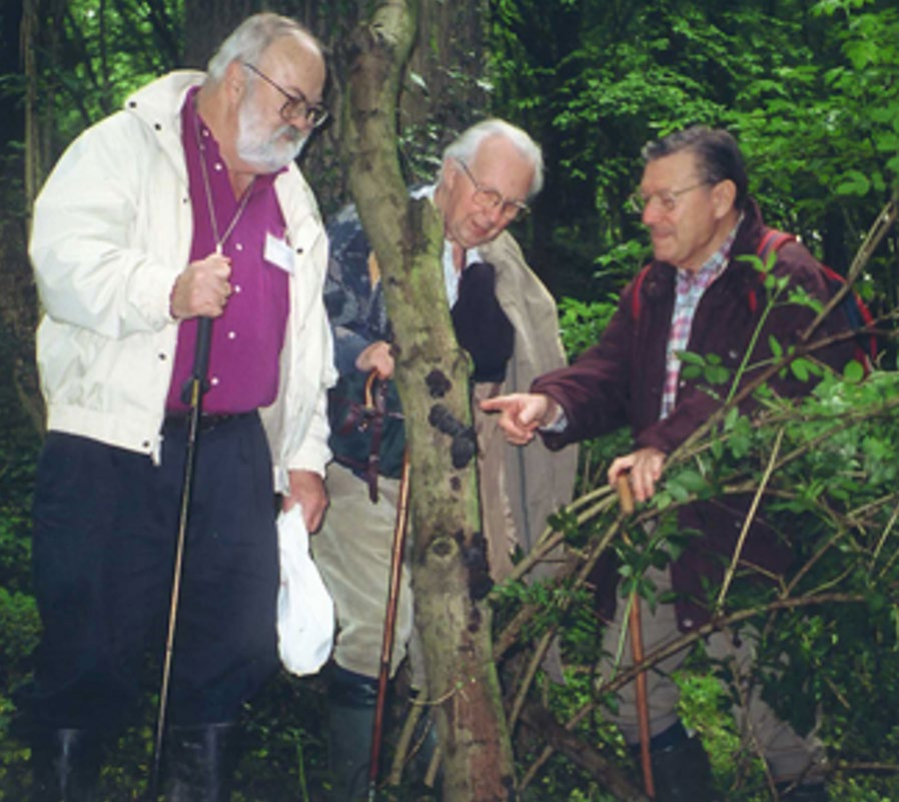
Jack D. Rogers (*left*) with the German amateur mycologists, Siegfried Woike (*centre*) and Hartmund Wollweber (*right*) in the Neandertal near Wuppertal, Germany in Summer of 2000, admiring a luxurious collection of *Daldinia concentrica*

Jack D. Rogers, an eminent scientist whose work has greatly influenced mycological taxonomy in the past decades, lamentably passed away on 14 June 2021. He retired in 2013 with great honors at Washington State University (Pullman, USA) after 50 years of successful teaching and research. Many of his accomplishments have already been mentioned by Glawe and Ammirati (2008) in a “Festschrift” honouring his 70th birthday. Hence, I will now focus here on Jack’s most important scientific results and their importance for the development of mycological taxonomy.

I first met Jack in 1994 at IMC4 in Vancouver, when we discussed my work on nematicidal activities of a *Daldinia* sp. that I presented in a poster from my PhD thesis. We subsequently started a collaboration that has laid the groundwork for my own taxonomic research. I will never forget when he visited us in the Neandertal valley in 2000 and we showed him our local collection sites where we picked *Daldinia concentrica* and other European *Xylariales.*

Jack was born on 3 September 1937, and started his mycological career working on aspects that only indirectly relate to taxonomy. His early papers dealt with cytology of selected species of stromatic *Xylariales*, including the genera *Hypoxylon*, *Xylaria,* and allies. These fungi became the main subject of his taxonomic studies, and he became one of the foremost experts in the field. He has co-authored several multi-authored papers on the higher-level phylogeny of fungi and summarized his taxonomic concepts in various important and timely review articles. However, in retrospect, his monographs on important *Xylariales* genera (in particular *Hypoxylon s. lat.*; Ju and Rogers 1996) appear even more valuable. They helped the gaps between classical “macromycete” taxonomy and the molecular era via the establishment of holomorphic species concepts. Jack dedicated several decades to the meticulous studies of both old type material and freshly collected specimens in order to relate the taxonomic concepts of the nineteenth century to the “modern” era. He and his students, above all Yu-Ming Ju and Felipe San Martín, have done outstanding work on the characterization and cultivation of *Xylariales* from around the world. They provided numerous valuable descriptions that now allow other researchers to establish asexual/sexual connections in this highly diverse and ecologically versatile group. Later comprehensive phylogenetic studies were also based on their materials (Hsieh et al. 2005, 2010). This work, and especially the availability of living cultures, paved the way towards our modern studies with multi-locus phylogenies and phylogenomics. We can now elucidate the life-cycle of the endophytic stages and other ecological phenomena, since the cultures can be subjected to genome sequencing, while little information can be obtained from the DNA sequencing of old stromata. Many of the phylogenetic relationships that Jack had predicted from careful morphological studies in the 1980s and 1990s have been confirmed in our recent work on polyphasic taxonomy, including multi-locus phylogeny, chemotaxonomy and phylogenomics (Wendt et al 2018; Wibberg et al. 2021).

Further reminiscences by another of Jack’s colleagues who also specialized in these same fungi were included in *IMA Fungus* on the occasion of his 80th birthday (Whalley 2017).

**Marc Stadler**

(marc.stadler@helmholtz-hzi.de)

### Kálmán Géza Vánky (1930–2021)

(Fig. 27)

**Fig. 27:**
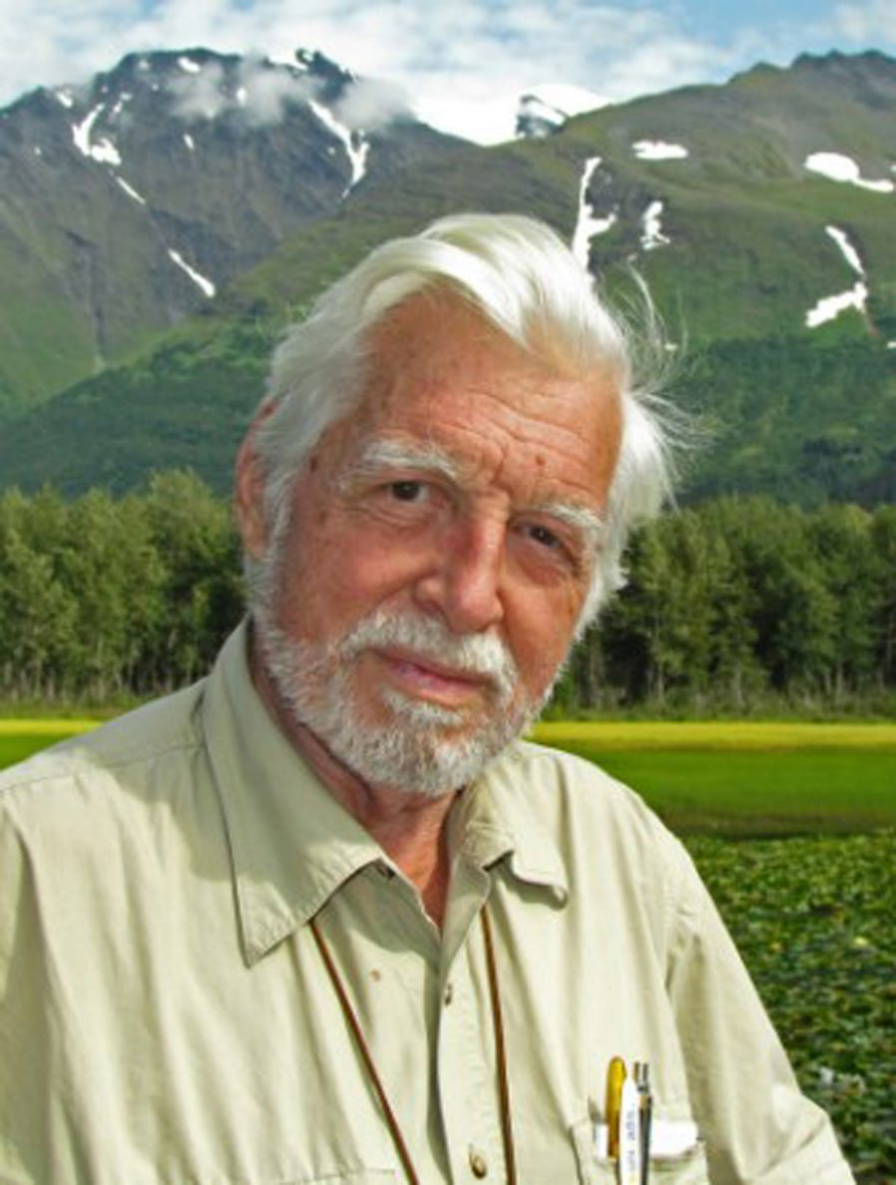
Kálmán Vánky

Kálmán Géza Vánky, born on 15 June 1930 in Székelyudvarhely (Odorheiul Secuiesc; Transylvania, Romania), passed away on 12 October 2021. Kálmán studied biology and medicine and practised medicine, mainly as a General Practitioner, but became interested in plant diseases as a hobby having been stimulated by lectures of Traian Săvulescu (1889–1963) in Bucharest. He focussed on the smut fungi (*Ustilaginomycotina*), a group on which he became the world’s leading authority, and with which his name is now almost synonymous, as he produced a succession of extraordinarily comprehensive detailed and well-illustrated major works.

In 1969 he moved to Sweden where he worked as a physician, but then, following nearly 30 years of “free-time activity”, he secured a three-year scholarship to Uppsala University in 1982–85 where he obtained a PhD for his studies on Carpathian *Ustilaginales* primarily under the guidance of J. A. Nannfeldt (Vánky 1985). He then moved to Germany in 1986 to devote himself exclusively to smut fungi from all over the world and was provided with facilities at the University of Tübingen by the late Franz Oberwinkler. He published a remarkable 234 papers and 10 books, in which some 46 new genera, 410 new species, and various higher taxa were introduced. His most important works were undoubtedly his extraordinarily thorough account of European smut fungi (Vánky 1994), and the monumental 1458-page monograph *Smut Fungi of the World* (Vánky 2012; see *IMA Fungus* **3**: (34), 2012)—with detailed nomenclators, descriptions drawings, photographs, SEM micrographs, and host lists for the world’s 1650 known smut species.

Fundamental to his work, was the establishment of a private fungarium, *Herbarium Ustilaginales Vánky* (HUV), which he started in Romania in 1954, continued to develop in Gagnef, Sweden (1970–1986) and then Tübingen (1986–2013); by 2013 it included 22 050 specimens, including many types (amongst them portions of types from other collections, an issue that occasioned considerable controversy). That year the collections were relocated to the Queensland Plant Pathology Herbarium, Indooroopilly, Qd, Australia (BRIP; *see IMA Fungus* **4**: (38), 2013). In 1975 he also started an exsiccate, *Ustilaginales exsiccata*, in which he eventually distributed 1350 numbers, in all some 76 000 specimens, to the world’s major mycological collections.

In recognition of this work, he was made an Honorary Member of the Hungarian Academy of Science, and awarded the Officers Cross of the Hungarian Order of Merit on 12 October 2020. Our thoughts are with his wife Christine, children, and grandchildren at this time.

### Bodo Wanke (1941–2021)

(Fig. 28)

**Fig. 28:**
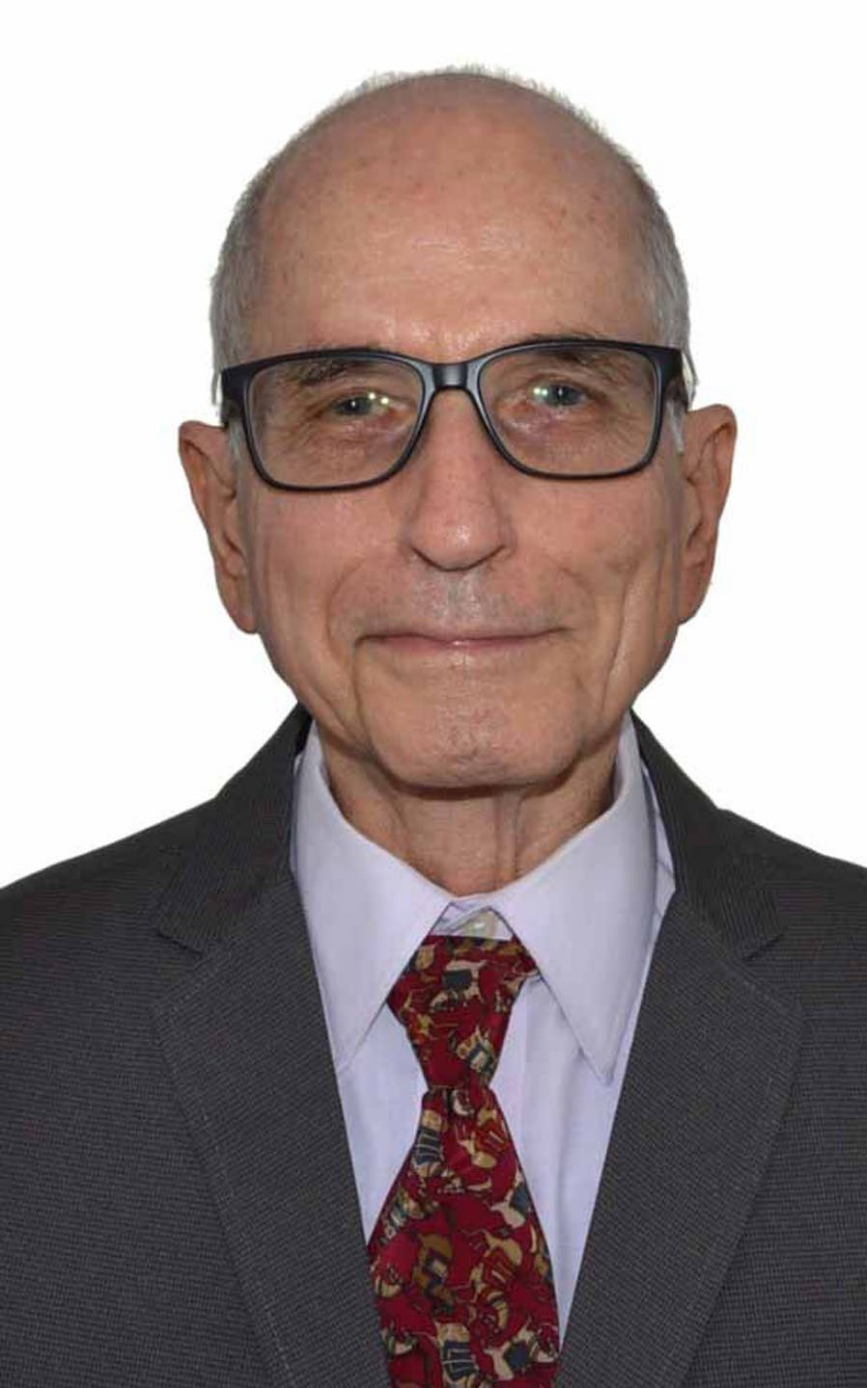
Bodo Wanke

Bodo Wanke, emeritus researcher at the Evandro Chagas National Institute of Infectious Diseases (INI), Oswaldo Cruz Foundation (FIOCRUZ), Laboratory of Mycology, and co-ordinator of the National Reference Laboratory for Systemic Mycoses (CGLAB/SVS/MS), passed away from complications of COVID-19 on 22 July 2021. Born in 1941 in the Brazilian State of Espirito Santo, he graduated in Medicine at the Federal University of Rio de Janeiro (UFRJ) where he also obtained his master and doctorate degrees in Infectious and Parasitic Diseases. In 1980 he founded the Laboratory of Mycology at INI, Rio de Janeiro, where he also was professor in Clinical Research in Infectious Diseases.

Bodo Wanke’s research focused on understanding the interactions between host and pathogens and the environment, encompassing the One Health approach. As an expert in the diagnosis of mycoses, he devoted more than 40 years to the study of endemic mycoses in Brazil, mainly coccidioidomycosis, paracoccidioidomycosis, histoplasmosis, cryptococcosis, and sporotrichosis. He was the first to detect coccidioidomycosis in Brazil. He lead a research programme aimed at detecting and monitoring coccidioidomycosis microepidemics in armadillo hunters in the State of Piauí (Brazil). Over his long research career, he broadened his researches into different areas through countless collaborations within Latin America, the USA, Europe, and Australia, together tackling a wide variety of mycological problems. As a result of his field and clinical studies, he was hugely instrumental in establishing a large mycology reference culture collection at FIOCRUZ. In order to provide mycology an official platform in Brazil, he cofounded the Brazilian Society of Mycology for which he also served as president. He published his research findings in medical mycology in over 160 peer-reviewed publications and 53 book chapters. He was an Honorary Member of the Brazilian National Academy of Medicine (ANM) and of the International Society for Human and Animal Mycology (ISHAM). His devotion to the training of young people and transmitting his vast knowledge of medical mycology made him an exemplary leader in this field. Foreseeing the huge possibilities offered by the advances in molecular biology to open up new ways for the faster and more accurate diagnoses of mycoses, in 2013 he established the “International Course on Molecular Methodologies for Epidemiology and Diagnosis of Invasive Fungal Infections”, which has since been taught yearly at FIOCRUZ, with an increasing number of national and international students. Proof of this are dozens of undergraduates, master, and doctoral students—an entire generation of medical mycologists in Brazil who give credit to Bodo Wanke’s outstanding and influential mentorship. For over 25 years he travelled intensively through several counties in the semi-arid and Cerrado ecoregions of the Brazilian State of Piauí, following the track of endemic mycoses in that region. He also was tireless in working to bring early diagnosis of cryptococcosis to remote areas, undertaking several trips with his team to isolated communities in the Amazon, offering free medical advice and organizing essential treatment whenever it was needed. A serene, generous, and humble character, always open to listen and offer advice to anybody who needed it, Bodo will be sorely missed by the mycological community of Brazil and globally.

**Wieland Meyer**

(wieland.meyer@sydney.edu.au)

## BOOK NEWS

### Genetics and Biotechnology

***Edited by J. Philipp Benz and Kerstin Schipper. 2020. Third edition. [The Mycota. vol. 2.] Cham: Springer Nature. Pp. xxii + 452, illustr. 59 (42 col.). ISBN 978-3-030-49923-5 (hbk), 978-3-030-49924-2 (ebk). Price: £ 139.99 (hbk), £ 111.50 (ebk).***

(Fig. 29)

**Fig. 29:**
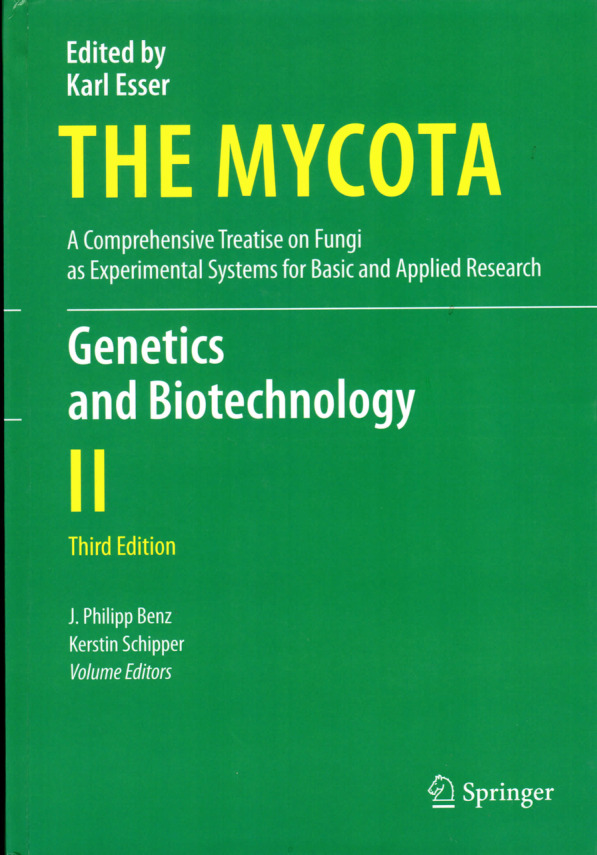
*Genetics and Biotechnology* (2020)

This is the fifth of the 15 volumes of *The Mycota* to be issued as a third edition, and brings the total number of volumes in the series to 36. The first appeared in 1995 and the second in 2004. In such a rapidly moving area of mycology, it is not surprising that there is much new to report 16 years on. While badged as a third edition, it is more a collection of in-depth review articles, many on topics which have largely emerged in the years since the second. Of the 17 chapters presented here, none precisely cover what was included in the second. Indeed, only one of the 73 contributors to the third, Gerhard Braus, was also involved in the second.

The chapters are organized into two sections: Molecular Genetics and Biotechnology. In the first these cover an array of diverse topics including chromatin structure and function, accessory chromosomes, the circadian clock, small RNAs, self/non-self recognition systems, genomics of partner biology in AM fungi, secondary metabolism and development, and a particularly valuable synopsis of the current state of fungal genomics. The Biotechnology section reflects how advances in our understanding of fungal genetics are being exploited, or have the potential to be so: as hosts for heterologous production of proteins and secondary compounds, in developing new avenues for drug discovery and bio-blocks for ontogenetic systems, the use of yeasts to produce a staggering array of substances (primary and secondary compounds, recombinant proteins, and fatty acids), biodeterioration and bioconversion of lignocellulose substrates, biotechnological possibilities of marine fungi, and an especially exciting overview of the potential of anaerobic gut fungi.

As in previous releases of new editions of volumes in the series, while the text is presented to the highest standards, many of the illustrations leave something to be desired—especially some coloured figures which are excessively reduced and include text on coloured blocks.

The volume also includes an obituary of Karl Esser, who passed away in December 2019 at the age of 95 years (see also pp. 30–31), prepared by Ulrich Kück and seven other of his former students and colleagues. He launched *The Mycota* along with the late Paul Lemke in 1994, and was still working, and managing the present volume, just a few weeks before his death. The series has come to be an extraordinary resource for mycology as a whole with the diverse authoritative reviews included, but the issue of its future is not touched on here. As I have indicated before, my personal view is that the chapters would have been better presented in a special journal that would both make them more accessible, less expensive, and able to be issued in a timely manner than have to wait until a particular volume was being compiled. It will be interesting to see what happens to the series in the future....

### Fungi and Trees: their complex relationships

***By Lynne Boddy. 2021. Stonehouse, Gloucs: Arboricultural Association. Pp. xiv + 306, illustr. (mainly col.). ISBN 978-0-900978-70-8. Price: £ 45.***

### Fungi on Trees: a photographic reference

***By David Humphries and Christopher Wright. 2021. Stonehouse, Gloucs: Arboricultural Association. Pp. xiii + 338, illustr. (full col.). ISBN 978-0-900978-71-5. Price: £ 45. Price (both volumes): £90.***

(Fig. 30)

**Fig. 30:**
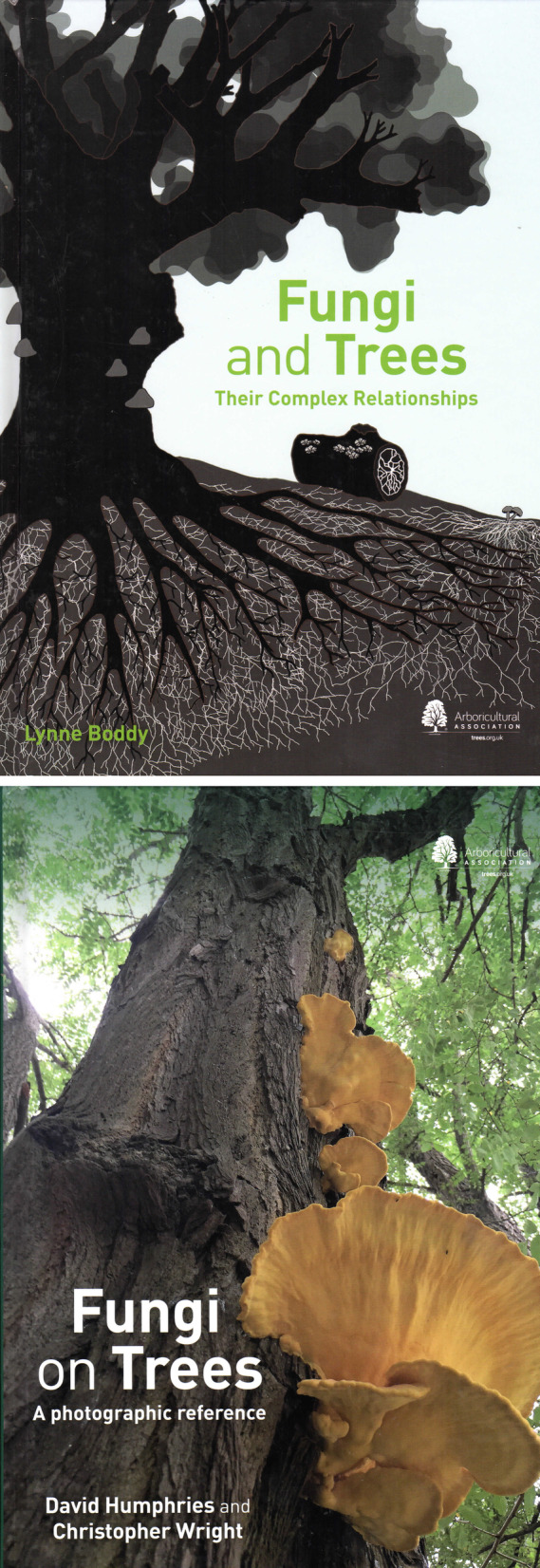
*Fungi and Trees* and *Fungi on Trees* (2021)

This splendidly produced large-format pair of complementary volumes has been published by the UK Arboricultural Association. They have been prepared to increase the knowledge of those working with trees about their importance to Earth surface processes and other organisms, and also to facilitate the correct identification of fungi, especially macromycetes growing directly on trees.

The first volume, by Lynne Boddy, whose career has been devoted to the study of fungi in and on trees, earned her an award from the Association this year. Following a basic introduction to fungi and how they feed, reproduce, and disperse, she then goes on to explain how leaves, roots and wood provide homes for fungi. The diagrams and photographs make this the most comprehensive treatment of fungal habitats in trees I have seen, and it also covers wood anatomy and explains how trees age and the fungi associate with different stages of their lives. A treatment of the beneficial relationships between fungi and trees follows, which covers the full range of mycorrhizal types, not only ectomycorrhizas, how to manage them, lichens, mycoheterotrophic plants, and endophytes. The major types of tree diseases are explained, leading to a superb overview of emerging diseases and how pathogens have invaded Europe—and flagging up the issue of biosecurity and the difficulty of ever being able to have meaningful checks on imported stock. I did not, however, note any mention of the carriage of spores into the country on birds, clothing, vehicles, or packaging; our trees will always be at risk from invasives, which may not even be known to science and so have no names. There is then a series of chapters focussing on interactions amongst tree-associated fungi and other organisms, including outcomes of inter-fungal wars (one of Lynne’s specialities), wood decay and fungal communities, heart-rot and hollowing, and sapwood decay in living trees. I was especially pleased to note the heads-up: “people often make the mistake of assuming that a fungus fruiting on a living tree is a pathogen, but if the fungus is feeding on dead central tissue it is acting saprotrophically not pathogenically” (p. 190). Tree-surgeons sadly are often too ready to recommend felling a tree just because some bracket fungus is present.

The final two sections deal in turn with environmental change and issues of conservation and management. She covers the different situations in which trees occur, traditional management practices, changes in basidome production patterns in relation to climate change, host switches, range changes, effects of nitrogen deposition and other pollutants, and introductions around the world of mycorrhizal fungi in particular. While I was pleased to see the effects of sulphur dioxide air pollution on lichens and how they could be used to estimate its severity covered, it would have been good to have drawn attention to how an upsurge of nitrogen and ammonia-loving lichens indicates when levels of those pollutants are a concern; abundant yellow-orange lichens on bark do not indicate the air is clean! The risk categories used in conservation status assessments for fungi are explained, and case studies provided of threatened fungi and of threatened habitats—not only dead wood and veteran trees, but further mycophagous and saproxylic organisms of all kinds. Finally, good practices are discussed, not only for forest management, with a flow chart for options of how best to deal with standing or fallen trees in urban settings (p. 278), veteranisation treatments (p. 280), and even translocation of fungi for conservation purposes. The work closes with a glossary, and I was pleased to see also a series of comprehensive indices. The layout, and standard of the colour photographs is truly superb although the quality of some of the drawings might have been improved, and each chapter has a summary of key points at the start and a carefully chosen further reading list at the end.

The whole is a real *tour-de-force*, with Lynne’s passion shining through every page, and I cannot recommend it too highly. One topic that she does not cover is identification, which is the province of the second volume of this pair, *Fungi* ***on*** *Trees* as opposed to *Fungi* ***and*** *Trees*. The authors’ names may not be familiar to many mycologists, but this has been prepared by two people intimately involved, and with a huge experience of, the management of trees: David Humphries as Trees Management Officer for the City of London Open Spaces and Christopher Wright, a Senior Arboricultural Consultant. They start by cautioning that this is primarily a work concerned with the British Isles, and that while much will apply to other northern temperate habitats in Europe, that will not be the case for the more southern Mediterranean regions. The first two rather brief chapters provide a short overview of tree-associated fungi and their determination; the section on microscopic methods is provided by Andy Overall, author of *Fungi: mushrooms & toadstools of parks, gardens, heaths and woodlands* (Overall 2017) and which would serve as a useful adjunct to this new volume.

The bulk of the book, roughly 80%, is devoted to what are delightfully termed “species biographies” of 100 selected species. These are divided into two categories, annuals and perennials, and within each subdivided pragmatically by coloured marginal tabs into brackets, crusts, cushions, clubs and balls, polypores, mushrooms, jellies, toothed, and slime moulds. Each species is allotted two, or in some cases three or even four, pages which include a dash-board like series of notes at the start with the scientific and common names, classification, seasonality, main host, decay type, frequency, and an innovative thumb-nail showing where they occur on a tree. Each has a box including a description, area affected, significance, additional hosts, similar species, and key synonyms. The descriptions are oriented to the field observer, and unfortunately the only actual measurements given are those of the sizes of basidiomes and there is no information on the spores apart from the colour. Many mycologists will find this frustrating, especially when microscopic techniques have been explained in the second part of the book, and these can be critical for the confirmation of the identity of some of the selected species. I appreciate, however, that some practising arboriculturalists might have found too much microscopical information off-putting. What is really valuable here, is that the bulk of the species pages are occupied by superb colour photographs selected from the staggering 25,000 David indicates he has so far (p. vii). These show different stages of development and variations in habit, undersides as well as views from above, and they are a real delight to see. At the end of the volume, in addition to an index, there is a tabulation of common fungi associated with particular trees that will be a helpful pointer to users. This volume is an incredible achievement and one which all tree surgeons should have to hand before pronouncing a tree as “diseased” and in need of felling. I do hope that it will get the circulation it really merits.

The Arboricultural Association are to be congratulated on publishing these two volumes which will be of great benefit to mycologists as well as arboriculturalists, and at a reasonable price considering the lavish production.

### Humongous Fungus

***By Lynne Boddy. 2021. London: Dorling Kindersely. Pp. 64, illustr. ISBN 978-0-2414-6040-5. Price £ 12.00.***

(Fig. 31)

**Fig. 31:**
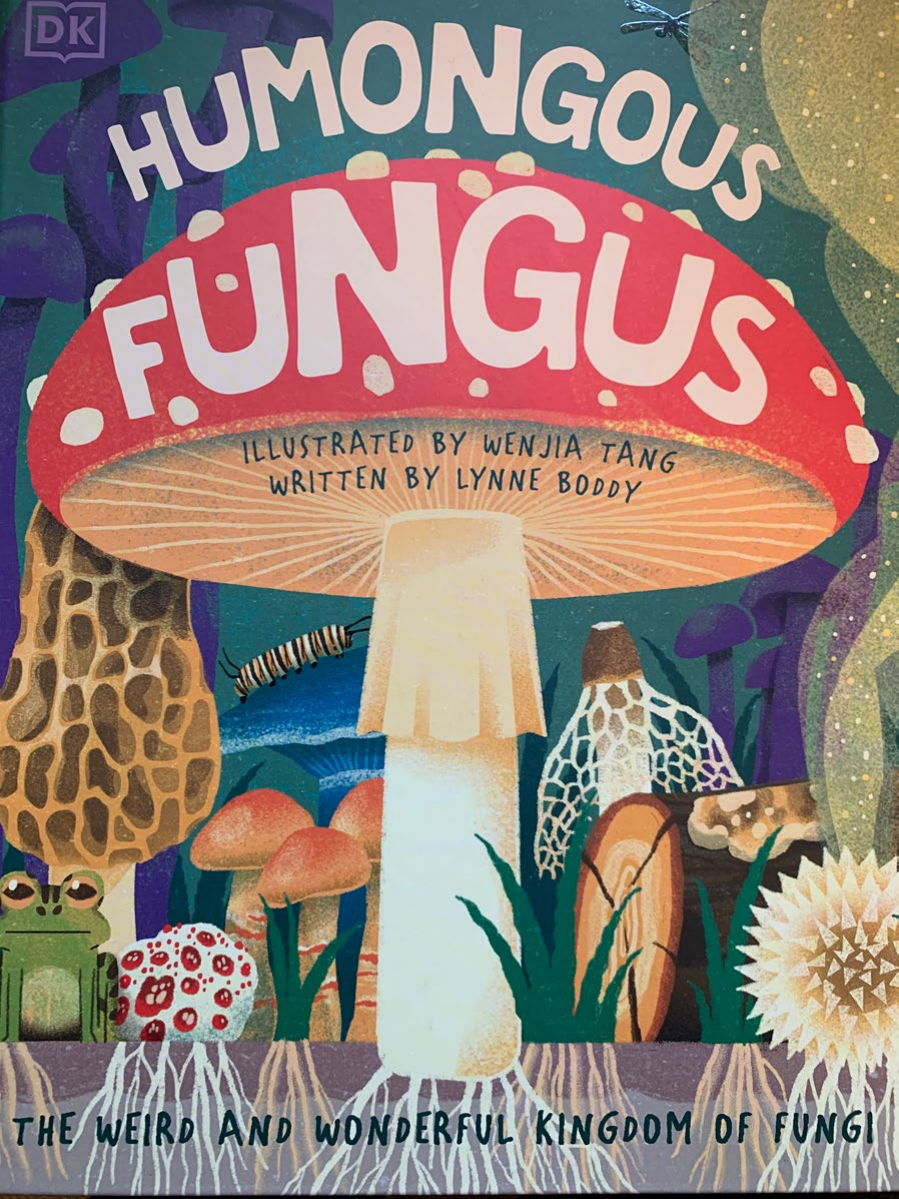
*Humongous Fungus* (2021)

This delightful little book is aimed at 7–9 year-olds and aims to introduce them to fungi and to appreciate their importance, from their role in the emergence of plants on land to the destruction of our crops (and the perhaps impending “bananageddon”) and applications such as plastic-eating “ecowarriors”. As one would expect from Lynne, there is much on their relationship with trees, but also their value in anaerobic digestion in animal guts. It includes pull-out tabs with fun fungal facts and is illustrated by photographs and also art work by experienced illustrator Wenjia Tang who now lives in New York. The book’s publicity claims this will “intrigue and amaze young readers, and open their eyes to the fungi thriving all around them” including a “magical tour of the forest floor”. I am sure that it will, and also enable them to surprise their teachers with fun “fungal facts”.

### Healing Mushrooms: a practical guide to medicinal mushrooms

***By Richard Bray. 2020. ISBN 9-798670-261661. Pp. x + 213, illustr. Hamburg: Monkey Publishing. Price: £ 10.74.***

(Fig. 32)

**Fig. 32:**
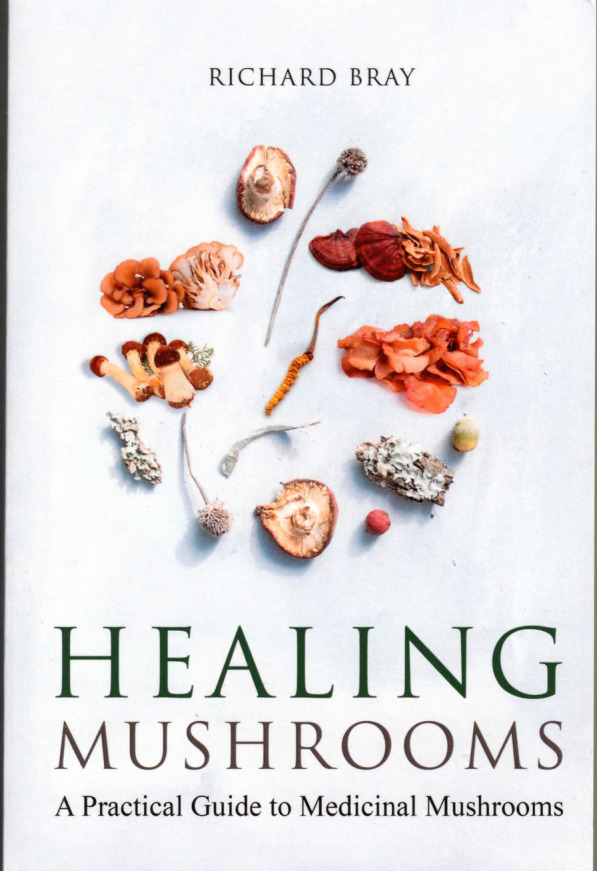
*Healing Mushrooms* (2020)

This book, described on the back cover as by a "herbalist and bestselling author", aims to introduce the "28 most powerful mushrooms you can add to your diet to maximise your health gains". The two core sections of the book are an overview of these species and then a section organized by "ailments".

In the overview, each is introduced with the scientific and selected common names, in most cases with half-tone illustrations and notes on reported main applications. The ailments section forms the largest part of the book, and 65 are considered from ageing to wounds and include numerous serious medical conditions. Information on dosage, the type of mushroom product, and published reports of their efficacy are compiled. The list of references is quite impressive with 337 entries, but the reason why some are duplicated is obscure; one appears in no less than 11 times.

There are cautionary notes on collecting and sourcing mushrooms, and a separate section addresses preparation methods including extracts and teas. I would, however, have wished to see more attention paid to the issue of misidentifications, which can be a particular hazard. Even commercial products may not be prepared with the species producers think they are using. Also, when considering preparations, the amounts of material required may vary depending on whether they come from mycelium, ground or sliced basidiomes, dry spores, or cracked spores.

In summary, an informative compilation, but one to be used only with considerable caution.

### Life at rock surfaces: challenged by extreme light, temperature and hydration fluctuations

***Edited by Burkhard Büdel and Thomas Friedl. 2021. Walter de Gruyter, Berlin.[Life in Extreme Environments vol. 9.] Pp. xiv + 244, illustr. (mostly col.). ISBN 978-3-11-064261-2 (hbk), 987-3-11-064646-7 (PDF), 987-3-11-0646264-3 (ebk). Price: 121.45 € (hbk).***

(Fig. 33)

**Fig. 33:**
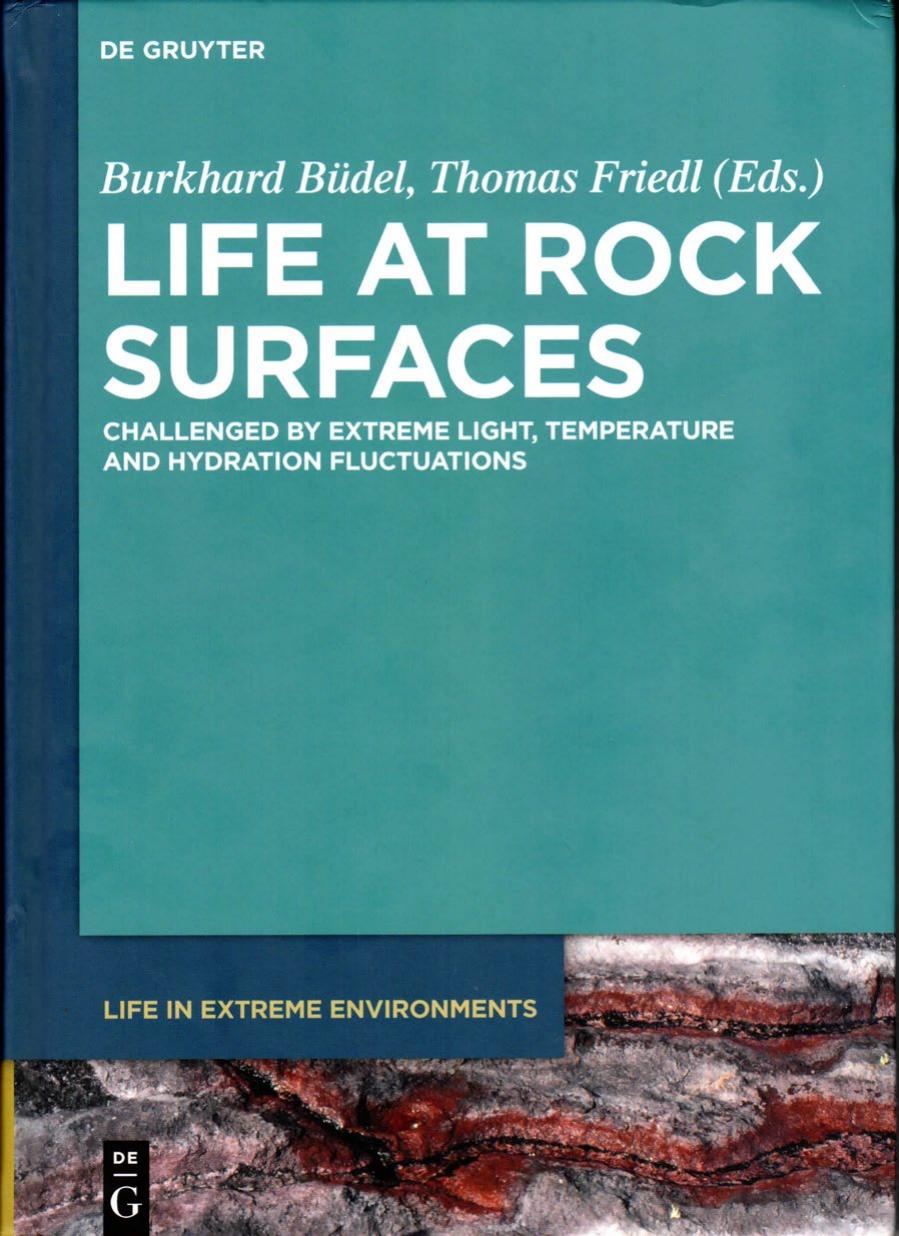
*Life at Rock Surfaces* (2021)

The complexity of the microbial communities inhabiting rock surfaces ("lithobionts") has been increasingly recognized over recent decades, and so an overview by some of the foremost contributors to this explosion of knowledge is most welcome; 18 authors from various countries in Europe and South Africa have combined to produce nine chapters to produce this synthesis.

The niche is more complicated than might at first be presumed, involving organisms that can be categorized by their positions: on the surface (epilithic), just below the surface under translucent crystals or pebbles (hypolithic), or inside rocks and soil crusts (endolithic). The endolithic organisms may be in fissures or cracks (chasmoendolithic), pre-existing cavities or pores (cryptoendolithic), actively penetrate into hard substrates (euendolithic), or on the underside of soil crusts (hypoendolithic). These categories are explained in the first chapter, with most helpful photomicrographs, and the wide range of evolving and increasingly sophisticated methods now used in their exploration are described. This first chapter also covers mineral interactions, especially bioweathering, while the second is devoted to the hypolithic habitat which is so important in desert soils and primarily composed of various bacterial organisms (including cyanobacteria).

A series of chapters then address the situation in different groups of organisms. Phylogenetically diverse black fungi, many described in recent years, melanins giving some protection from harmful sunlight, and which have been well-studied in Antarctica but are also of concern for their biodeterioration of stonework (especially of monuments of heritage importance. Cyanobacteria, including a novel and valuable key and notes on the characters of the 53 genera involved, which will also be of value to lichenologists. Lichens, self-sustaining ecosystems that are often the first colonizers of rock surfaces, either epilithic or endolithic depending on whether the rock is siliceous or calcareous. High alpine lichens where freezing and water availability are key issues. Complex lichen communities, on different rock types and interactions between them. Eukaryotic algae, including red algae and diatoms, with lists of genera. And finally desiccation-tolerant vascular plants.

The chapters are extensive referenced, well-edited, and many have great colour photographs or microphotographs. As stated on the back cover, this is a indeed a “unique overview of various organismal groups interacting with the rock surface”. This will now be THE go-to work for anyone contemplating research on these ecologicaly very specialized and so fascinating organisms. The editors are to be congratulated on pulling such a fascinating and informative synthesis together.

#### Atlas of Clinical Fungi: the ultimate benchtool for diagnostics

***By G Sybren de Hoog, Josep Guarro, Josepa Gené, Sarah A Ahmed, Abdullah M S Al-Hatmi, Maria J Figueras and Roxana G Vitale. 2020. 4*** ^***th***^ ***edition. Hilversum: Foundation Atlas of Clinical Fungi. 2 vols. Pp. 1598, illustr. (many col.). ISBN 978-94-93226-12-8. Price: 275 €.***

(Fig. 34)

**Fig. 34:**
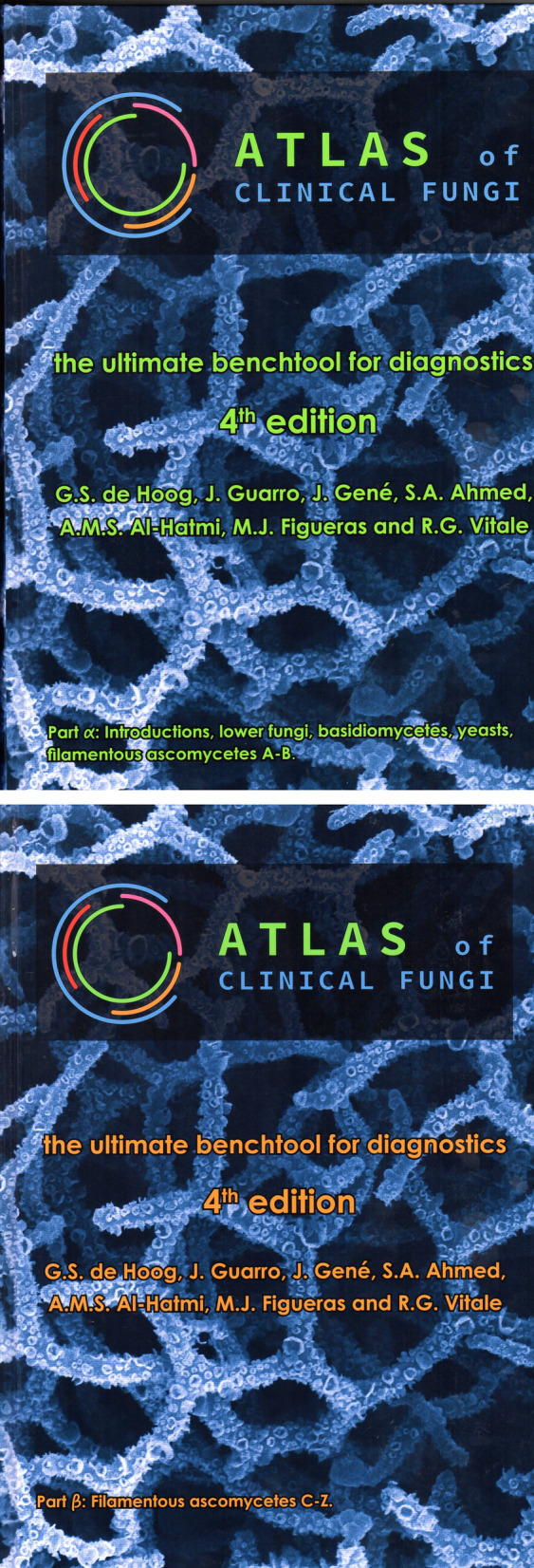
*Atlas of Clinical Fungi* (2020)

This *Atlas* is now well-established as the keystone reference work, the *vademecum,* and “go to” source of clinical mycology, since it first appeared in 1995. The third edition of this was issued as a CD-ROM back in 2009, while the second edition was issued in hardback in 2000. The second edition was a comparatively modest single volume of 1126 pages, which has morphed here into two massive volumes, with 42% more pages in the fourth, and which together weigh 7 kg.

The first sections together amount to a textbook covering the range of diseases and kinds of mycoses, their histology, laboratory techniques, phenotypic and molecular methods of examination, media recipes, available antifungals (including side effects and interactions), and recommendations for treatment. There are keys to facilitate morphological identifications in tissue sections, the genera treated, a systematic arrangement, and a tabular comparison of names adopted in the first and second (but not the third) editions. Some 700 species are treated in depth, the “lower fungi”, basidiomycetes, yeasts, and filamentous ascomycetes A-B in the first volume (Part α), and filamentous ascomycetes C-Z in the second (Part β). The genera are arranged according to the systematic hierarchy, and then alphabetically within each family; personally, I would have preferred all to be in an alphabetical series, but arrangement in this way does have the advantage in enabling keys to genera and notes applicable at the family level to be brought together. In the case of larger genera, such as *Aspergillus,* keys to species are also provided. The individual species entries have information on colony characteristics, microscopic features, pathogenicity, growth characteristics, antifungal susceptibility, key references, nomenclature, line drawings, and generally first-rate colour photographs of both colonies on pertinent media and microscopic features. The second volume ends with a particularly comprehensive glossary of terms, an amazing 36-page compilation of doubtful names and unconfirmed clinical cases, and an index to the included species that will greatly facilitate users unfamiliar with systematic placements locating entries.

This is the first edition to be issue since the ending of the separate naming of different morphs of the same species in 2011, and it is pleasing to see this implemented throughout. As a result, this work will help ensure the adoption of single-name nomenclature in the medical mycology community.

The establishment of a not-for-profit Foundation to enable this *Atlas* to be published and have a continuing future is a wonderful and magnanimous initiative of the authors. This has meant that the hardback volumes, of which I understand only a rather limited number were printed, could be offered at a very reasonable cost for such a complex and well-produced work. Furthermore, this was made available at a modest online-only cost during 2021, and from 1 January 2022 it is scheduled to become available online (www.atlasclinicalfungi.org) *entirely free of charge* – an immense service to medical mycology which will surely aid the diagnosis and treatment of numerous cases to an extent not previously possible. The authors deserve our thanks for not only producing such a scholarly fundamental reference work, but for their generosity.

### Emerging Plant Diseases and Global Food Security

***Edited by Jean B. Ristaino and Angela Records. 2020. ISBN 978-0-89054-637-6 (hbk), 978-0-89054-639-0 (ebk). St Paul, MN: American Phytopathological Society Press. Pp. vi + 305, illustr. (most col.). Price: US$ 249 (hbk).***

(Fig. 35)

**Fig. 35:**
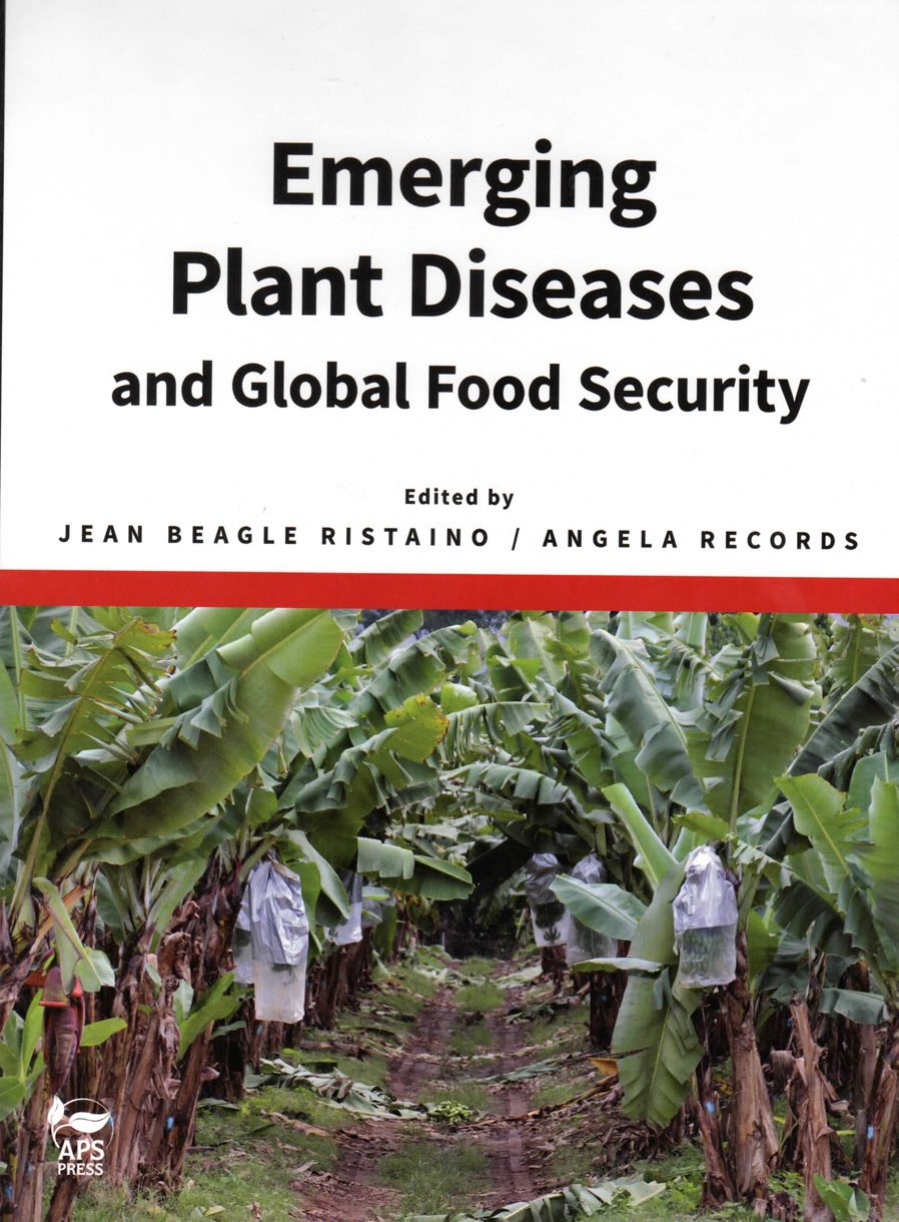
*Emerging Plant Diseases and Global Food Security* (2020)

It is not always appreciated how vulnerable some of the key crops we depend on for food are to fungal pathogens. In the first section of this book, chapters focus on the vulnerability of supplies and the farmers in the poorest regions of the world, the current and worrying extent of global losses from pests and diseases, and the difficulty of adapting disease management systems in a period of global climate change—the challenge of developing climate-smart agriculture integrating disease resistance, control, and forecasting.

In-depth explorations of the situation in selected species are provided for the cases of wheat rust, maize leaf necrosis, late blight, cassava viruses, Panama disease of bananas, and coffee rust. The spread of the Ug99 race of wheat stem rust (*Puccinia graminis* f.sp. *tritici*) is a particular and increasing concern with a race between evolving fungal genotypes and resistance genes in the host. The situation with late blight (*Phytophthora infestans*) is complex with the genotype frequencies changing year by year on potatoes (and to a lesser extent on tomatoes) and even in different parts of even a small country such as the UK. Panama disease (*Fusarium oxysporum* f.sp. *cubense*) is now threatening the long-resistant Cavandish banana cultivar due to a newly emerged race TR4; breeding for resistance is a particular challenge as this not always correlates with taste and resistance to bruising during transport. Resistance has also broken down against coffee rust (*Hemileia vastastrix*) in Central America, with the climate favouring particular races of the rust and also adversely affecting the most commonly grown Lempira cultivar; technical solutions may be the way ahead but may mean production becomes unprofitable.

The final group of chapters focuses on approaches to addressing and limiting the prospect of major disease episodes. These include "Plantwise", an international initiative led by CAB International to promote "plant health systems" and plant clinics, collecting data and providing an advisory service to help monitor outbreaks. An overview of geospatial analysis to monitor disease spread, a review of models to predict epidemics and optimize detection and management. And finally an examination of the situation in blast diseases caused by *Magnaporthe oryzae* pathotypes on diverse grass species, including rice, wheat, millet, perennial rye-grass, and tall fescue; this masterly overview shows the host specificity of the different pathotypes and discusses how resistance may be managed and outbreaks contained, but also highlights the prospect of spread to crops such as barley and oats should it become adapted to cooler climates.

All the chapters are by teams of specialists, well-edited, fully referenced, and superbly laid-out and illustrated to the high standards we have come to expect from APS Press. I cannot commend this work too highly, and trust that it helps increase political awareness of just how vulnerable are some of our staple foods, and how fragile is this element of the global food supply. Funding for basic research and field experiments should surely be increased to a level commensurate with the risk these diseases pose to global food security.

### Hidden Kingdom: the surprising story of fungi in our forests, homes, and bodies

***By Keith A. Seifert. 2022. Vancouver: Greystone Books. Pp. xiv + 288. ISBN 978-1-77164-662-8 (hbk), 978-1-77100-663-5 (ebk). Price: CAN $ 34.95 (hbk).***

(Fig. 36)

**Fig. 36:**
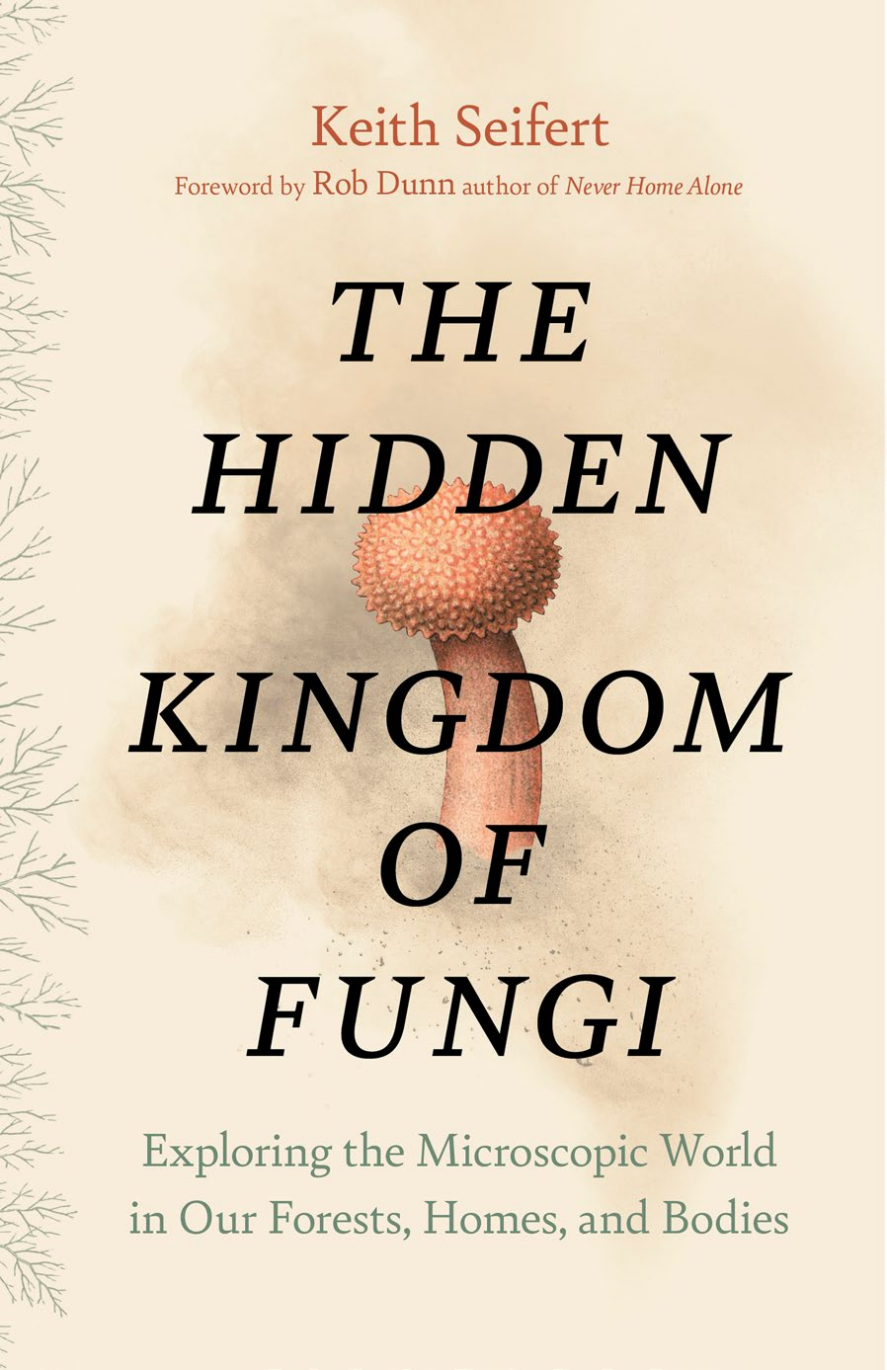
*The Hidden Kingdom of Fungi* (2022)

This new public-oriented book is due to be published on 22 May 2022, but I have been privileged to see an uncorrected proof copy so can draw it your attention now. It is a roller-coaster authoritative and gripping exposé of fungi, and how we and our environments interact with them daily in multifarious, but often unseen and unappreciated, ways. It reflects Keith’s wide interests in aspects of pure and applied mycology in its breadth, and this sets it apart from other texts of the genre. The style is engaging, with personal insights, and his enthusiasm and fascination for fungi emerges from every page. Topically, he also provides an exciting vision of how fungi might contribute to a more sustainable Earth. There are inevitably a few quibbles other mycologists will have as he strays out of his comfort zone, but it would be invidious to highlight any here as those I found were all rather minor. This book has the potential to heighten public awareness and respect for fungi, and merits a wide circulation in bookstores around the world.

### Trends in the Systematics of Bacteria and Fungi

***Edited by Paul Bridge, David Smith and Erko Stackebrandt. 2021. Wallingford: CAB International. Pp. xviii + 346. ISBN 978-1-789-24498-4 (hbk), 978-1-77100-663-5 (ebk). Price: £ 115 (hbk).***

(Fig. 37)

**Fig. 37:**
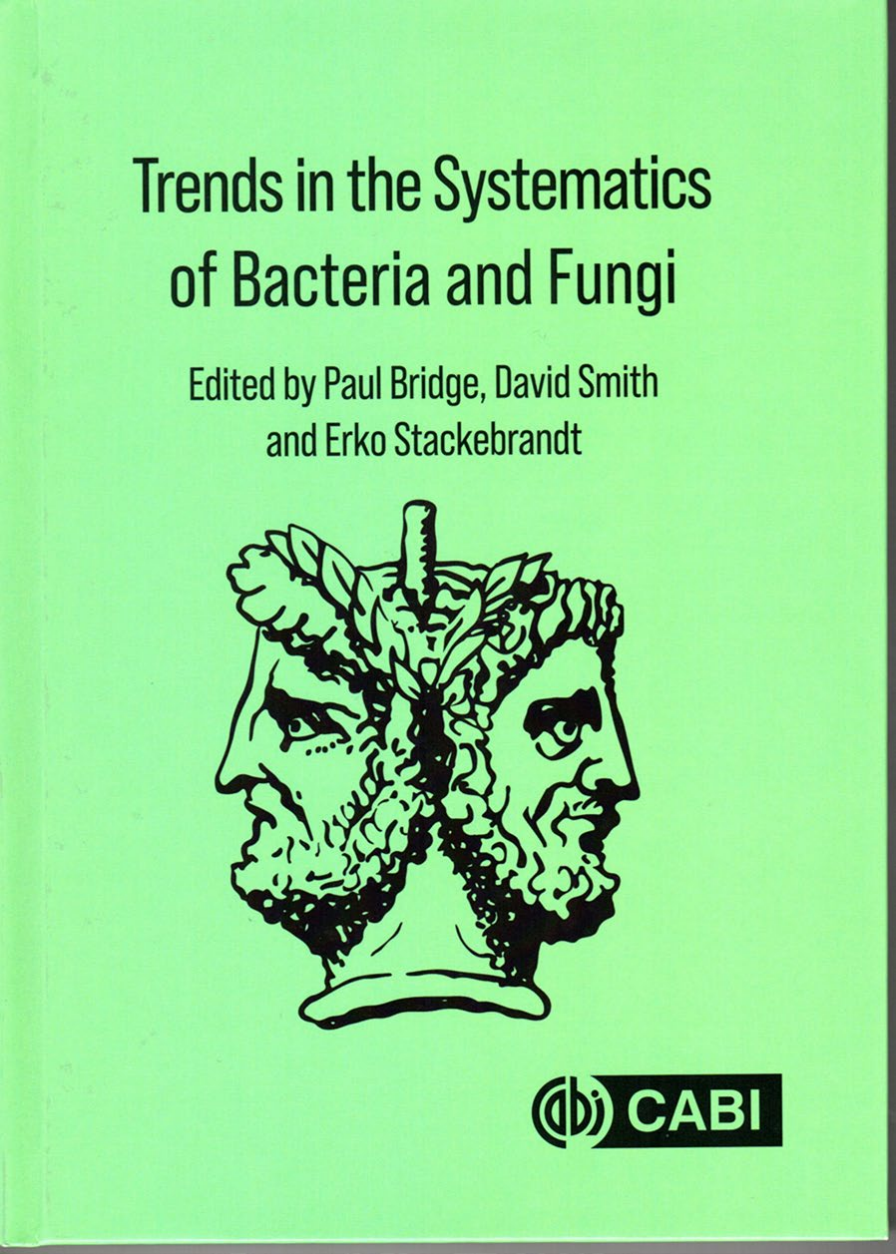
Trends in the systematics of Bacteria and Fungi (2021)

I have always been keen to learn from approaches and methodologies being used by systematists working on organisms other than fungi, something that goes back to my student days at Leicester University in the 1960s. There I learnt of novel methods being used by several of the leading systematists of the day working on bacteria, fossils, and plants—and adopted several in my PhD on a lichen-forming genus. The first editor started his reach on bacteria and yeasts before turning to filamentous fungi, so was well-placed to take the lead on such a volume; the second is one of the foremost specialists on culture preservation procedures; and the last, one of the best-known names in prokaryote nomenclature and systematics.

The editors have marshalled a further 49 contributors from diverse countries to put together a volume of 18 chapters in what they refer to as “a pivotal time for microbial systematics” (p. xvii). The reliance of prokaryote nomenclature on type cultures is flagged up in the first chapter as a key problem due to the demand for names from those working with environmental samples which are not known in culture; the issue is coming to a head as the International Commission on Systematics of Prokaryotes (ICSP) voted against making any much provision last year—it is now a case of “reconciliation or divorce” (p. 13), with a separate “UnCode” for unculturable organisms now being debated. This same topic is highlighted at the end of an overview of fungal identification by Tom May, where he notes that discussion is currently polarized (p. 27) and the issue of these so-called “dark taxa” is returned to again in Chapter 12 (pp. 204–205). The following chapters deal with data sources on names; the issue of preservation of reference strains, their management, and quality control procedures; the value of older molecular sequences; and the role and services of culture collections. Two chapters examine the use of MALDI-TOF and similar approaches in bacteria and fungi, respectively, which remain constrained in fungi by issues surrounding standardization and a lack of reference databases. A contribution on chemotaxonomy focusses on biomarkers in bacteria, especially fatty acids, lipids, polyamines and sugars, but surprisingly there is no equivalent contribution on fungi where compounds such as polyketides have proved especially valuable markers.

Whole-genome sequencing is becoming increasingly recognized as *the* way for the near future, and this is addressed in four contributions, but is clearly much more advanced in the case of bacteria than fungi; mycologists considering such approaches could benefit from contemplating the experience in bacteriology. The always thorny issue of what is a “species” is considered in separate chapters on bacteria and fungi, but while these are far too short to adequately address various definitions and approaches, extensive reference lists are provided. The “species question” is one of those returned to in the final reflections of the editors on the future direction of bacterial and fungal systematics, where they also discuss the problems in obtaining sequence data from historic collections, curation of names, global networking of strain information, and difficulties in working with and exchanging living material as a consequence of international treaties (especially the Nagoya Protocol)—and the work-arounds some countries have developed.

There are inevitably aspects which might have been addressed more fully, for example improvement in the stability of fungal names through protected lists, and initiatives being taken to promote good practice by the International Commission on the Taxonomy of Fungi (ICTF), a body that most surprisingly I could not see mentioned at all. The book does, however, provide much for mycologists to reflect on and learn from their bacteriologist counterparts, and in that it clearly succeeds here, and the editors are to be congratulated in pulling such a work together.

## NOTICES

1. *MycoNews* is compiled by David L. Hawksworth as Editor-in-Chief, and to whom all material for consideration for inclusion in *MycoNews* should be sent directly by e-mail.
2. Books for possible coverage in the Book News section should be mailed to David L. Hawksworth at Milford House, 10 The Mead, Ashtead, Surrey KT21 2LZ, UK; works issued only as e-books are not normally included, but reviews prepared by others will also be considered if sent to him.
3. Reports of new genome sequences intended for inclusion in the *Fungal Genomes* compilation should be sent directly to Senior Editor Brenda Wingfield as e-mail attachments and not submitted through Editorial Manager.
4. All unsigned items in *MycoNews* can be attributed to the compiler, David L. Hawksworth.
